# Developing ether and alcohol based extraction chromatography resins for purification of antimony-119 in nuclear medicine

**DOI:** 10.1186/s41181-025-00379-y

**Published:** 2025-08-13

**Authors:** Aivija Grundmane, Illarion Dovhyi, Lauren Aburto-Kung, Steffen Happel, Caterina F Ramogida, Valery Radchenko

**Affiliations:** 1 Department of Chemistry, Simon Fraser University, 8888 University Dr W, Burnaby, BC V5A 1S6 Canada; 2 Life Sciences Division, TRIUMF, 4004 Wesbrook Mall, Vancouver, BC V6T 2A3 Canada; 3 TrisKem International, 3 Rue des Champs Géons ZAC de, L’Éperon, Bruz, 35170 France; 4 Department of Chemistry, University of British Columbia, Vancouver, BC V6T 1Z4 Canada

**Keywords:** Antimony-119, Radiochemical separation, Extraction chromatography, Radiopharmaceutical therapy

## Abstract

**Background:**

Antimony-119 (^119^Sb, t_1/2_ = 38.19 h) is an Auger electron emitting radionuclide of interest for radiopharmaceutical therapy. It can be directly produced by proton bombardment of tin-119 using low energy cyclotrons. The radiochemical separation methods available for recovering ^119^Sb from the bulk Sn target material are lacking, particularly with respect to matrix suitability for further applications.

**Results:**

Eight new resins were successfully synthesized, evaluating combinations of two different resin support materials with three different chain lengths of ethers (dibutyl, dipentyl, dioctyl) as well as fluorinated alcohol as the impregnated extractant. All resins showed good stability, losing less than 1% of functional groups in HCl and water. Seven out of eight synthesized resins showed excellent capacity, retaining tens to hundreds of milligrams of Sb per gram of resin. Seven out of eight synthesized resins, as well as one of the resin supports showed good separation between Sb and Sn based on the distribution coefficient studies as well as dynamic elution studies.

**Conclusions:**

Our tested resins may be applied for the separation of radiotracer amounts of Sb from bulk Sn target material. We propose the dibutyl ether functionalized divinylbenzene copolymer resin support (DBE-300 resin) as the best candidate, based on the following characteristics: (1) quantitative, concentrated elution of Sn offering compatibility with recycling of enriched ^119^Sn material; (2) near-quantitative (98%), concentrated recovery of ^119^Sb in ethanol, providing a matrix resistant to hydrolysis, easy to convert by evaporation, as well as toxicologically insignificant pre-clinically and even clinically.

**Supplementary Information:**

The online version contains supplementary material available at 10.1186/s41181-025-00379-y.

## Background

Since the discovery of radioactivity in the early 20th century scientists have always been trying to harness the destructive power of radioactivity and channel it into something useful. The medicinal effects of radioactivity were quickly hypothesized, and attempts were made to utilize radiation to treat different diseases a century ago (MacKee 1921). Building upon these early concepts, radiopharmaceutical therapy (RPT) has emerged, focusing on destroying diseased tissue by localized delivery of a radionuclide of choice to the diseased site. It has gained much popularity in recent years, expanding the pharmaceutical toolbox in the fight against cancer. There are two types of radioactive decay currently utilized for RPT clinically – beta (β^−^) and alpha (α) particles. The characteristics of these two types of decay are very distinct and can best be described by the different energy ranges and the metric of linear energy transfer (LET), which expresses the energy deposited per unit of distance (Ku et al. 2019). Energy of β^−^ particles is varied, ranging from a few keV all the way up to a few MeV depending on the radionuclide. LET of β^−^ particles is quite low, depositing only 0.1–1 keV per µm traveled. However, β^−^ particles can travel relatively longer distances in the body (2000–10000 μm or 200–1000 cells) requiring less localized delivery systems to still be effective. α particles have higher energy (several MeV) and exhibit a much more localized dose, traveling distances of 50–100 μm or 5–10 cells, requiring more sophisticated delivery systems, but pack a much stronger punch (LET = 50–240 keV/µm) allowing for a more localized dose. For this reason, α particle emitting radionuclides like actinium-225 (^225^Ac, t_1/2_ = 9.92 d, E_α_ = 5830 keV (I = 51%), 5793 keV (I = 18%) have gained much popularity in recent years (Kratochwil et al. 2016). This has prompted many scientists to look for approaches to deposit even more localized doses in the fight against metastatic tumor growth. Auger electrons (AEs) are a type of decay often observed in radionuclides that undergo radioactive decay by electron capture (EC) and typically occur as cascades of many AEs emitted per one radioactive decay. AEs have a considerable LET at 4–26 kev/µm and travel only < 0.5 μm in the human body, which is equivalent to less than a cell diameter, offering the capability of RPT on a cellular basis.

Antimony-119 (^119^Sb, t_1/2_ = 38.19 h) is an AE emitting radionuclide of interest for RPT (Grundmane et al. 2024; Thisgaard and Jensen 2008; Randhawa et al. 2021; Bernhardt and Forssell-Arons 2001; Carbo-Bague and Ramogida 2021; Filosofov et al. 2021). It decays by EC and is accompanied only by one gamma ray at 23.870 keV (I = 16.50%) and numerous low energy (< 30 keV) conversion electrons (CEs) and X-rays. The low energy of these emissions presents a very localized dose profile, well suited for RPT (Thisgaard and Jensen 2008; Bastami et al. 2022). For theranostic applications, ^117^Sb (t_1/2_ = 2.8 h, E_γ_ = 158.6 keV (I = 85.9%), E_β+_ = 261.9 keV (I = 1.81%) has been proposed as the most suitable radioantimony imaging analogue for both single photon emission computed tomography (SPECT) and positron emission tomography (PET), although ^118m^Sb (t_1/2_ = 5.0 h, E_β+_ = 146.8 (I = 0.160%) may also hold potential for PET.

**Fig. 1:**
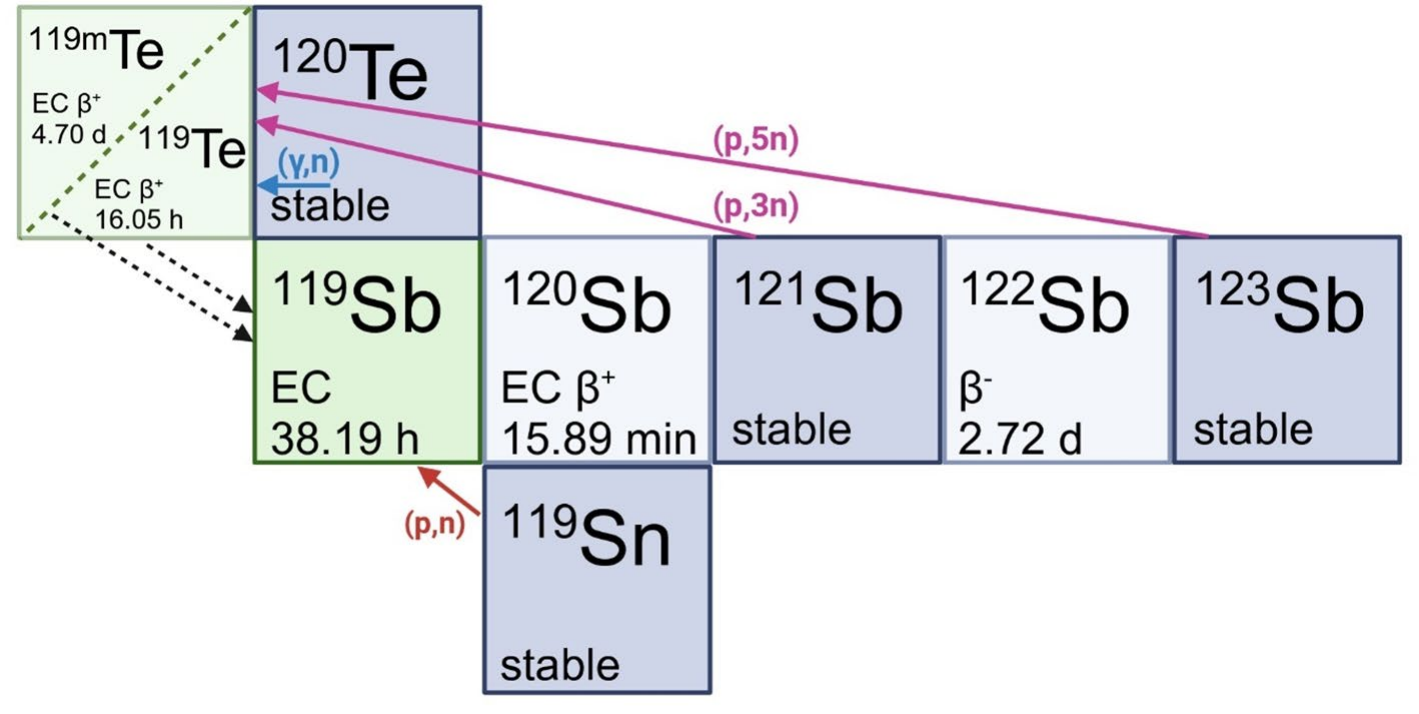
Possible production routes of antimony-119 from different target materials (dark blue), including the direct production route (red arrow) and the production of tellurium-119 m generator (magenta arrows). Adapted from Ref (Grundmane et al. 2024). Copyright (2024) with permission from Wiley-VCH GmbH

There are two main routes of producing ^119^Sb (Fig. 1). The direct production route from tin-119 (^119^Sn, stable) is more accessible, requiring low energy cyclotrons capable of producing 10–12 MeV protons, corresponding to the peak of the ^119^Sn(p, n)^119^Sb cross Sects. (Thisgaard and Jensen 2009; Thisgaard et al. 2011). Production of a tellurium-119m (^119m^Te, t_1/2_ = 4.7 d) generator is also possible through the ^121^Sb(p,3n)^119m^Te and ^123^Sb(p,5n)^119m^Te reactions, but requires access to cyclotrons capable of producing protons of 30 MeV or higher and will not be discussed further in this publication (Bennett et al. 2019; Yi and Miller 1992; Mosby et al. 2017).

After production using the direct production route, the Sn targets are typically easily dissolved in hydrochloric acid, resulting in a solution containing milligrams to grams of ^119^Sn and nanograms to micrograms of ^119^Sb. This is not a matrix or purity that is acceptable for further steps of radiopharmaceutical development, hence requiring separation, concentration and matrix conversion of ^119^Sb. Several methods for the separation of radioantimony (^1xx^Sb) from Sn target material (^nat^Sn) have been reported, purifying and recovering the ^1xx^Sb with varying degrees of success and complexity. These separations as well as an earlier prototype of the work in this publication have been evaluated elsewhere (Randhawa et al. 2021; Grundmane et al. 2025). Ultimately, for most of these separations, various challenges with large elution volumes, and unsuitable matrices containing chelating agents resulted in ^1xx^Sb poorly suited for pre-clinical and even chelation studies (Thisgaard and Jensen 2008; Baluev et al. 2003). Other separations were able to overcome these issues, but required complicated setups under gas flow, limiting its future use and making automation unlikely (Olson et al. 2024). Building on a previously existing liquid-liquid extraction separation methodology, a dibutyl ether functionalized slurry-type resin was developed as a prototype for column chromatography applications, requiring Sb in the Sb(V) oxidation state for the separation to occur (Grundmane et al. 2025; Kostelnik et al. 2023). In this work we expand on this concept, synthesizing commercial quality resins and evaluating the effect of fluorinated alcohol functional groups, different ether chain lengths, and difference in resin supports for the separation of trace quantities of ^119^Sb from bulk Sn targets.

## Materials and methods

### Materials

All experiments involving radioactive substances were conducted by appropriately trained personnel in shielded TRIUMF laboratories certified for radiochemical use by the Canadian Nuclear Safety Commission.

Ultrapure water purified using a Millipore Milli-Q system (18 MΩ·cm) or Sartorius Arium Comfort system was used for all experiments conducted at TRIUMF or TrisKem, respectively. Trace metal grade hydrochloric acid (37 wt% in H_2_O, 99.999% trace metals basis) was used at TRIUMF throughout and was sourced from Honeywell or MilliporeSigma. Analytical reagent grade hydrochloric acid was used at TrisKem.

The silver backing material for targets was 14 gauge 0.999 fine silver and was sourced from RioGrande (Albuquerque, United States). The reagents necessary for target production, tin(II) sulfate (≥ 95%), sulfuric acid (96%, Suprapur grade), phenolsulfonic acid (65 wt% in H_2_O), gelatin (from porcine skin, powder, microbiology grade) and 2-naphthol (99%) were sourced from MilliporeSigma.

AmberChrom CG71 (100–150 μm) and CG300 (50–100 μm) resin supports were acquired from DuPont. Dibutyl (99%), dipentyl (99%), and dioctyl ethers (99%), and methanol (99%, synthesis grade) for resin synthesis were acquired from MilliporeSigma.

Antimony(V) chloride (99.999%, Alfa Aesar), and other standard element solutions (Ag, Al, Ba, Bi, Ce, Co, Cs, Cu, Er, Eu, Ga, Hf, La, Lu, Mo, Nb, Nd, Ni, Pb, Rb, Re, Sc, Sn, Sr, Th, Tl, U, Y, Yb, Zn, Zr) at starting concentrations from 1000 to 10,000 mg/L were purchased from Fluka Analytical and Fisher Scientific.

Sodium thioglycolate (≥ 96.5%) and ethylenediaminetetraacetic acid for elution tests were acquired from MilliporeSigma. Anhydrous ethanol was sourced from Commercial Alcohols.

### Instrumental techniques

Gamma (*γ-)* spectroscopy analysis was conducted at TRIUMF using an N-type co-axial high purity germanium (HPGe) detector from Canberra calibrated using ^152^Eu and ^133^Ba sources. As ^119^Sb is difficult to detect using conventional *γ-*spectroscopy due to the low energy of gamma emission (E*γ* = 23.9 keV), natural tin (^nat^Sn) targets were irradiated and ^120m^Sb (E*γ* = 197 keV) were quantified to understand its radiochemical behaviour instead. Although this does not allow for the direct quantification of ^119^Sb, the identical radiochemical behaviour of the different Sb radionuclides provides reliable information on the chemistry of ^119^Sb and distribution of activity throughout the experiment (e.g., percentage of ^1xx^Sb retained on the column during a separation). It should be noted that the main limitation of using ^120m^Sb as a tracer for ^119^Sb is that less of ^120m^Sb is produced and consequently the signal to noise ratio is lower, meaning that in cases where ^120m^Sb is measured at 0, there may still be minute amounts of ^119^Sb present that were not measured. Another benefit of using ^nat^Sn targets is the ^117m^Sn (E*γ* = 158 keV) co-produced, which was used as a radiotracer to quantify the separation efficiency of the ^nat^Sn targets. All samples were measured using the same time duration (10 min), and distance from the detector (5 cm), were diluted to the same volume (1.5 mL), and measured in the same type of vial to keep the sample geometry constant. The detection limit for each radionuclide was taken as the lowest reading experimentally measured at these conditions.

Inductively coupled plasma mass spectrometry (ICP-MS) analysis was conducted at TrisKem International using a PerkinElmer NexION™ 350X ICP-MS. All samples were prepared in 2% HNO_3_ containing 50 ppm of indium as an internal standard prior to analysis. Detection limit was found to be 0.03 ng/L for Sb and 2.8 ng/L for Sn.

Total Organic Carbon (TOC) analysis was conducted at TrisKem International using a Shimadzu TOC-L analyzer in the non-purgeable organic carbon (NPOC) mode. The detection limit and reproducibility of the instrument were 50 ppb and 1.5% or ± 50 ppb, respectively.

### Radioantimony production

^nat^Sn was electroplated on silver (Ag) backings using a conventional Sn(II) sulfate bath (Olson et al. 2021). These ^nat^Sn targets (~ 50–100 mg/cm^2^) were irradiated for 1 h using the 13 MeV cyclotron (TR13) at TRIUMF (Vancouver, Canada). Depending on the mass of the target used, up to 0.3 GBq of ^119^Sb were produced at end of bombardment, alongside other co-produced radionuclides such as ^117m^Sn and ^120m^Sb useful for separation monitoring (see Supplementary Information Table S1 for more details). The irradiated targets were left in the cyclotron for at least 18 h to allow for the decay of short-lived isotopes to minimize the dose for the cyclotron operators and chemists. After transport to a shielded fume hood, they were dissolved in 12 M HCl. The Ag backings are insoluble in HCl and were removed from the dissolved target solution. This results in a solution containing Sb(III) and Sn(II) chlorides in 12 M HCl. To achieve Sb(V) and Sn(IV) required for the separation chemistry, hydrogen peroxide was added to the target solution in 1:10 parts (v/v), resulting in Sb(V) and Sn(IV) chlorides in ~ 9 M HCl.

### Extraction chromatography resin synthesis and characterization

#### Synthesis

Two different resin supports and four different extractants were used to develop eight novel resins (Table 1). The extractant of choice was added to the resin support in a suitable ratio (w/w) in the presence of excess methanol. The resin was dried using a rotary evaporator while decreasing the pressure until the methanol and excess extractant were removed, and a dry, powdered resin was acquired.

**Table 1:** Composition of the eight extraction chromatography resins prepared and studied

Resin Support	Extractant	Resin Name
AmberChromⓇ CG71 100–150 μm (cross-linked aliphatic acrylic ester)	Dibutyl ether	DBE-71
	Dipentyl ether	DPE-71
	Dioctyl ether	DOE-71
	Fluorinated alcohol	TK401-71
AmberChromⓇ CG300 50–100 μm (divinylbenzene co-polymer)	Dibutyl ether	DBE-300
	Dipentyl ether	DPE-300
	Dioctyl ether	DOE-300
	Fluorinated alcohol	TK401-300

#### Characterization

The stability of all resins was evaluated by TOC analysis of the eluted fractions. To simulate a typical separation, as described in Sect. 2.4.4., the resins were packed in a 1 mL chromatography reservoir (see Supplementary Information Table S2 for the mass of each resin) and pre-conditioned with three column volumes (3 mL) of 12 M HCl. Without the addition of any Sb or Sn, 12 M HCl (10 mL) was passed through the column to emulate the HCl wash steps and collected. To test resin stability when eluting Sb in aqueous media, such as dilute acid or an aqueous chelating agent eluant like sodium thioglycolate, the HCl wash step was followed by elution in deionized water (10 mL), which was also collected. 1 mL of the HCl and deionized water elutes were diluted to 10 mL with deionized water and analyzed using TOC. To test resin stability when eluting Sb in organic media like ethanol, the experiment was conducted for one of the resins (DBE-300), with the HCl wash step followed by elution in deuterated ethanol (1 mL), which was collected and measured by ^1^H NMR.

The resin capacity for ^nat^Sb was determined using a dynamic column setup and calculated as mass of Sb retained per mass of resin (mg Sb/g of resin) using three different approaches. In all approaches, the resins were packed in 2 mL chromatography reservoirs (Table S2) and were pre-conditioned by passing through three column volumes (6 mL) of 12 M HCl. Breakthrough capacity refers to the mass of Sb retained on the resin before any breakthrough of Sb occurs after the addition of Sb in a stepwise fashion, i.e., it is the amount of Sb the resin can retain before any Sb is observed in the elute. After breakthrough capacity is achieved, the resin may still be able to retain more Sb, despite some Sb being not retained and found in the elute. This is referred to as full resin capacity - the total mass of Sb retained on the resin, even after partial breakthrough of Sb occurs after addition of Sb in stepwise fashion. Total resin capacity is similar to full resin capacity, but instead of being conducted in a stepwise fashion, a large amount of Sb is passed through the column all at once in a single load step. Breakthrough and full resin capacities were determined simultaneously, by stepwise addition of ^nat^Sb(V) in 12 M HCl solution (~ 20 mg/step, ~ 200 mg total, except for DBE-71, which was ~ 30 mg/step, ~ 300 mg total). In contrast, total resin capacity was determined by the addition of ^nat^Sb(V) in 12 M HCl solution in one step (~ 200 mg). Each elute step was slowly passed through the column and collected using a vacuum box. The elute fractions were then appropriately diluted for ICP-MS analysis, with the initial dilution steps using deionized water, and the final dilution step using 2% nitric acid containing an indium (In) internal standard. Theoretical resin capacity was calculated from the known weight of the functional group incorporated into the resin, assuming each molecule of the corresponding functional group can retain one Sb atom (Eq. 1).

1 $$\begin{array}{l}\:Theoretical\:resin\:capacity\:\left(g\:Sb\:per\:gram\:of\:resin\right)\\=\frac{Mass\:of\:extractant\:in\:a\:gram\:of\:resin\:\left(g/1\:g\:of\:resin\right)}{Molar\:mass\:of\:extractant\:\left(\frac{g}{mol}\right)}\times\:Molar\:mass\:of\:Sb\:\left(\frac{g}{mol}\right)\end{array}$$

#### Determination of distribution coefficients (K_D_) and rate of Sb uptake

Distribution coefficients (K_D_) were determined using three approaches: (a) for stable ^nat^Sb(V)/^nat^Sn and other elements using a multi-element standard containing 32 different elements; (b) for stable ^nat^Sb(V) using a single-element standard; and (c) for ^120m^Sb(V)/^117m^Sn(IV) using irradiated ^nat^Sn targets. Resin (50 mg ± 1 mg) was weighed and combined with a range of concentrations of 1 mL of either (a) ~ 1 mg/L stock of multi-element standards (0.01, 0.1, 1, 2, 3, 6, 8, 10 M), (b) ~ 1 mg/L stock of single-element Sb standard (6, 8, 10, 12 M), or (c) oxidized irradiated target solution containing nanograms of ^1xx^Sb and milligrams of Sn (0.1, 1, 2, 3, 6, 8, 10, 12 M). Multi-element standard solution was made by adding standard element solutions to 10 M hydrochloric acid. Single element ^nat^Sb(V) standard solution was made by adding Sb(V) chloride to 12 M hydrochloric acid and diluted appropriately to reach a ~ 1 mg/L stock. For K_D_ measurements using stable elements, samples were vortexed and shaken for 1 h, then centrifuged at 5000 rpm for 15 min. For K_D_ measurements using radiotracers, the samples were vortexed every 20 min during a period of 1 h due to the absence of a designated shaker. The layering of the resin and aqueous phase after extraction (i.e., whether the resin floated, sank, or was dispersed in the aqueous phase) was noted for each resin and concentration (Supplementary Information Table S3). The aqueous layer was then removed and filtered. For the stable element K_D,_ the filtrate was diluted with 2% nitric acid for ICP-MS analysis, while for the K_D_ with radiotracers, the filtrate was measured using γ-spectrometry. K_D_ values were calculated using Eq. 2.

2 $$\begin{array}{l}\:Distribution\:coefficient\:\left({K}_{D}\right)\\=\:\frac{({Concentration}_{initial\:solution}-{Concentration}_{aqueous\:phase)}}{{Concentration}_{aqueous\:phase}}\:\times\:\:\frac{{Volume}_{aqueous\:phase}}{{mass}_{resin}}\end{array}$$

Rate of Sb uptake for three of the resins (DBE-300, DOE-300, and TK401-71) was determined for stable ^nat^Sb(V) as well as ^120m^Sb(V) as per the K_D_ methodology, except taking care to separate the aqueous phase after extraction from the resin at discrete time points (1, 5, 10, 15, 30, 60 min) to assess how much time is needed to achieve maximum uptake of Sb on the resin. The rate of uptake was expressed as the percent recovery of Sb as a function of time.

#### Extraction chromatography

**Fig. 2:**
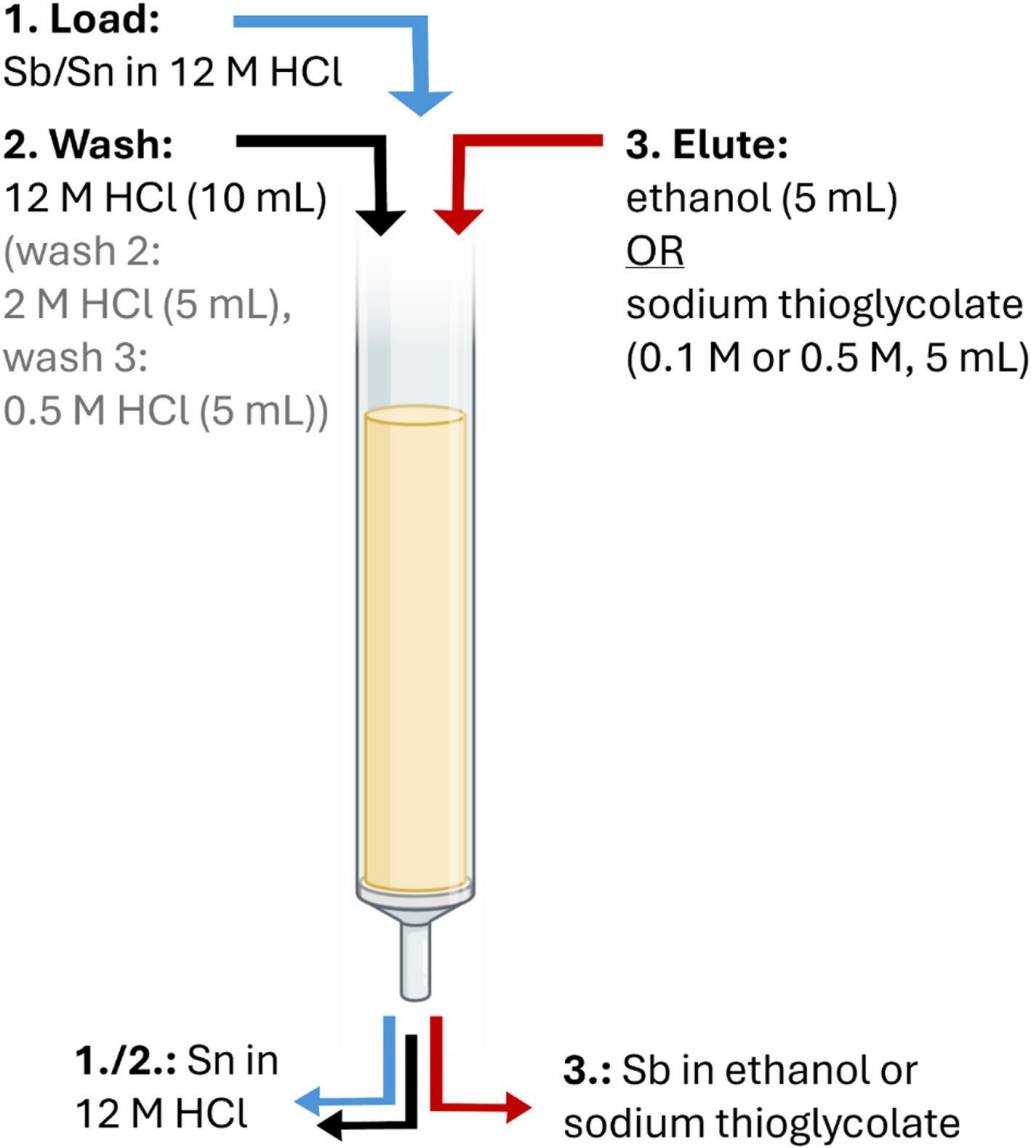
Summary of the elution conditions typically utilized throughout the study, showing the load matrix, wash step(s) to remove bulk Sn and eluants used for the recovery of Sb

Elution profiles were determined using two different approaches: using a stable multi-element standard solution containing ~ 1 mg/L of Sb, Sn, Fe, Nb, Mo, Zr, Cs, Sr, Ni, Co, Zn, Ga, La, Eu, Yb, U, Th and Ba in 12 M HCl or by conducting a radiochemical separation using an irradiated ^nat^Sn target. Resins were packed in 1 mL chromatography cartridges (Table S2) and were pre-conditioned by passing through three column volumes (6 mL) of 12 M HCl. The load step consisted of 1 mL of the multi-element standard or an aliquot of the oxidized target solution (10^2^ pg ^120m^Sb(V), 10^1^ pg ^117m^Sn(IV), 10^0^ mg ^nat^Sn(IV)), and using a vacuum box slowly passed through the column, collecting the eluted fractions. For elution profiles using stable multi-element standard solution, this was followed by 10 wash steps (12 M HCl), 5 elution steps with 2 M HCl, 5 elution steps with 0.05 M HCl, and 5 elution steps with 0.1 M sodium thioglycolate. Note that sodium thioglycolate acts as a reducing and chelating agent, recovering Sb as a Sb(III) thioglycolate complex. For the radiochemical separation, only the DBE-300 resin was investigated, and the 12 M HCl wash steps were followed by elution in either sodium thioglycolate (0.1 M or 0.5 M), or ethanol. For stable multi-element elution profiles, the eluted fractions were collected and diluted in 2% nitric acid for ICP-MS analysis, while for radiochemical separation they were collected and measured using γ-spectrometry. Improvements in Sb breakthrough upon loading were sought by using a syringe pump and constant (1 mL/min) flow rate for the load step. Elution optimization for whole target scale separations (~ 50 mg Sn) was conducted using DBE-300 resin and the success of the established separation chemistry using different size and geometry columns (1 mL, 2 mL, 4 mL), and different resin mass was evaluated. A scheme summarizing the elution profiles tested here can be seen in Fig. 2.

## Results

### Extraction chromatography resin synthesis and characterization

All eight resins (DBE-71, DBE-300, DPE-71, DPE-300, DOE-71, DOE-300, TK401-71, and TK401-300, Table 1) were successfully synthesized using the reported methodology. The degree of impregnation varied between the extractants; 23% or 26% of dibutyl ether used in the synthesis was retained on the DBE-71 and DBE-300 resins, respectively. Meanwhile, 82% or 75% of dipentyl ether was retained on the DPE-71 and DPE-300 resins, and 95% or 99% of dioctyl ether was retained on the DOE-71 and DOE-300 resins, respectively.

**Fig. 3:**
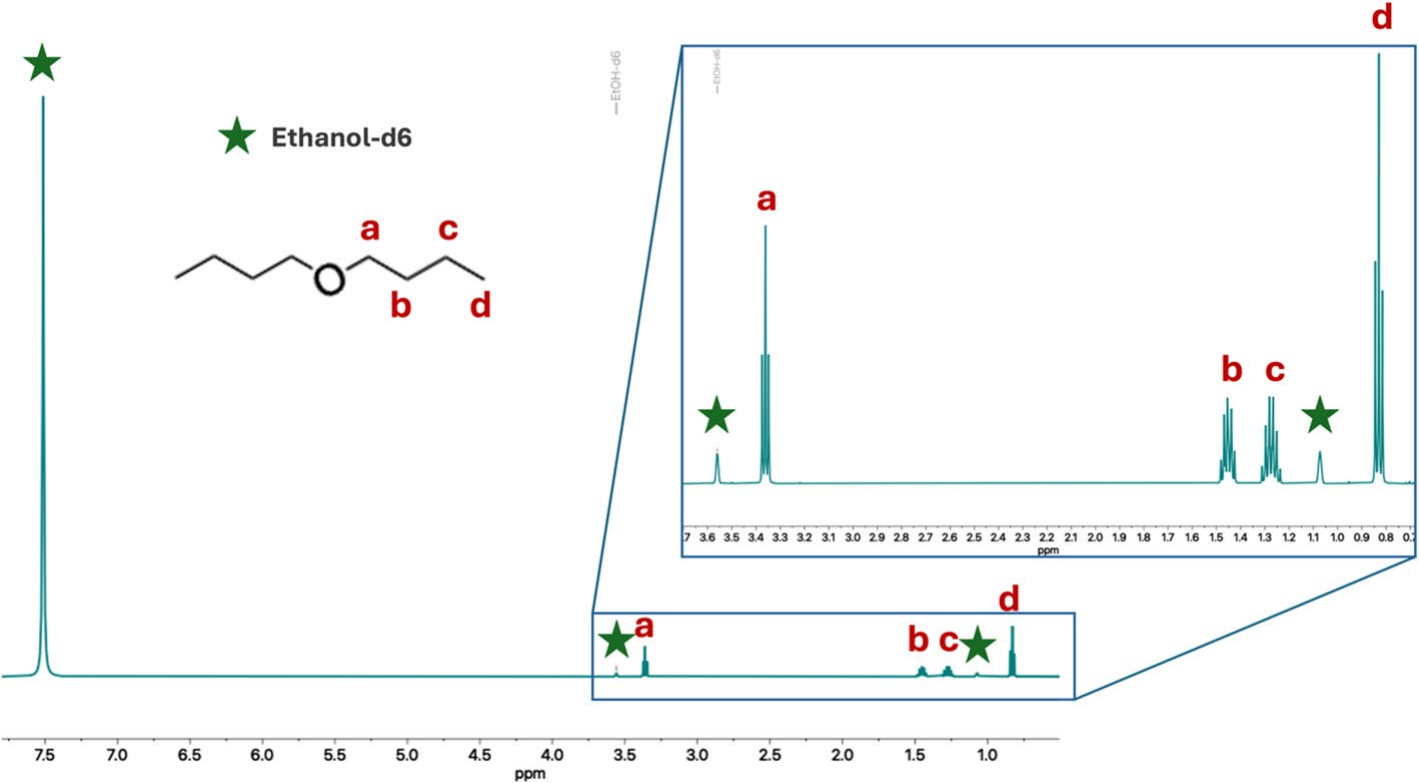
^1^H NMR spectrum (500 MHz, ethanol-d_6_) of the deuterated ethanol elute, showing trace amounts of dibutyl ether present

The stability of the resins was tested using TOC. To be considered of commercial quality, the resin must lose less than 1% of its extractant upon use. All resins met this requirement in aqueous media, exhibiting < 0.3% loss upon passing through 10 column volumes of concentrated HCl, and even less so in the following aqueous elution step (Table 2). When using ethanol as the eluent, trace amounts of dibutyl ether were present (Fig. 3), indicating partial loss of the ether functional group upon elution.

**Table 2:** Total organic carbon (TOC) analysis of the elute fractions passed through 1 mL resin columns (Fig. 2, see supplementary information Table S2 for corresponding resin masses), showing the amount of TOC in the elute fractions in ppm, as well as the calculated percentage of the corresponding extractant found in the elute, assuming all carbon in the elute as measured by TOC is attributed to the extractant

Resin Name	TOC in 10 mL 12 M HCl wash (ppm)	Percent of extractant in HCl wash (%)	TOC in 10 mL deionized water elute (ppm)	Percent of extractant in elute (%)
DBE-71	8.3	0.25	4.7	0.14
DPE-71	29	0.23	2.5	0.020
DOE-71	6.5	0.056	2.1	0.018
TK401-71	34	0.25	6.4	0.071
CG71 support only	10	-	0.78	-
DBE-300	8.4	0.28	1.9	0.064
DPE-300	13	0.15	1.7	0.020
DOE-300	6.3	0.063	1.0	0.010
TK401-300	29	0.25	8.2	0.046
CG300 support only	5.3	-	1.0	-

Another important measurement of resin viability for practical application is its capacity for retaining the elements of interest. All resins showed great capacity for Sb, retaining tens to hundreds of milligrams of Sb per gram of resin used (Table 3). These values are better or comparable to those of common commercial resins currently found on the market (e.g., Pb resin – 29 mg Pb/g resin) (Horwitz et al. 1994). Although equivalent amounts of Sb were added for full and total capacity experiments, the stepwise vs. 1 step addition may show difference in the resin capacity based on the physical kinetics of this experiment (e.g., full capacity for DOE-300 is almost twice that of its total capacity indicating that the resin was not saturated in the latter experiment). Regardless, at this stage these values are rough estimates of the potential resin capacity, meant to illustrate the magnitude of resin capacity rather than exact values. More replicates of these experiments would be needed to establish exact values and to make any meaningful observations on the difference between stepwise addition (full capacity) and 1 step addition (total capacity). Overall, based on the resin capacity and stability data all of these resins possess the integral qualities expected from a commercial product. Furthermore, the CG71 resin support itself also is able to retain Sb on the resin, resulting in larger capacity values for the CG71 supported resins.

**Table 3:** Resin capacity for retaining ^nat^Sb, expressed as milligrams of Sb per gram of resin (*n* = 1). Where the maximum capacity was not determined due to not reaching saturation of the resin, the values are listed as “>n”

Resin Name	Theoretical capacity (mg Sb/g resin)	Breakthrough capacity (mg Sb/g resin)	Full capacity (mg Sb/g resin)	Total capacity (mg Sb/g resin)
DBE-71	125	136	178	271
DPE-71	272	151	217	244
DOE-71	195	150	177	204
TK401-71	147	87	118	160
CG71 support only	0	70	> 202	> 276
DBE-300	138	117	163	140
DPE-300	259	139	150	159
DOE-300	200	83	108	61
TK401-300	134	0	52	59

### Determination of distribution coefficients (K_D_) and rate of Sb uptake

The K_D_ values were used as a screening tool for the understanding of elemental affinity towards the resins. While the purpose of developing these resins was to separate Sb from Sn, the behaviour of other elements was also evaluated to assess other potential applications. Table 4 shows a compiled overview of the suitability of each resin for the separation of different elements when using HCl as the mobile phase (see Supplementary Information Tables S4 – S13 for the corresponding K_D_ values).

**Table 4:** Summary of the separation capabilities of each resin based on the distribution coefficient (K_D_) data (see supplementary information Tables S4 – S13), identifying the effective concentration range of hydrochloric acid (HCl) for the separation to occur, listing elements that are sufficiently retained on the column for a separation to be developed, as well as the elements that can be separated from them at the given concentration of hcl. For elements where more than one experimental method was used to determine the K_D_ values (see Sect. 2.4.3.), the methods are denoted as (1) ^nat^Sb(m) and ^nat^Sn(m) for stable antimony and tin measurements from a multi-element standard denoted as (m), (2) ^nat^Sb(s) for stable antimony measurements from a single-element standard denoted as (s), and (3) ^120m^Sb and ^117m^Sn for radiochemical measurements. K_D_ values for all other elements were acquired using stable elements from a multi-element standard

Resin Name	Elements sufficiently retained on the column (K_D_ >500) at the given [HCl (M)]	Elements with no affinity for the resin (K_D_ < 50) at the given [HCl (M)]
DBE-71	[6–12]: ^120m^Sb [8–12]: ^nat^Sb(s) [6–10]: ^nat^Sb(m), Ga [1–10]: Tl	[0.1–12]: ^117m^Sn [0.01–10]: Bi, Ce, Eu, Hf, La, Lu, Nd, Th [0.01–8]: Ba, Cs, Co, Cu, Er, Pb, Ni, Re, Rb, Sc, Ag, Sr, U, Yb, Y, Zn, Zr [0.01–1; 3–8]: Al [0.01–3; 8]: ^nat^Sn(m) [0.01–6]: Nb [0.01–3]: ^nat^Sb(m), Ga, Mo [0.01–0.1]: Tl [0.1]: ^120m^Sb
DPE-71	[8–12]: ^120m^Sb, ^nat^Sb(s) [8–10]: ^nat^Sb(m), Ga [10]: Tl	[0.1–12]: ^117m^Sn [0.01–10]: Al, Ba, Bi, Ce, Cs, Co, Cu, Er, Eu, Hf, La, Pb, Lu, Mo, Nd, Ni, Re, Rb, Sc, Ag, Sr, Th, ^nat^Sn(m), U, Yb, Y, Zn, Zr [0.01–8]: Nb [0.01–3]: ^nat^Sb(m), Ga [0.1–0.5; 3]: ^120m^Sb [0.01–0.1]: Tl
DOE-71	[8–12]: ^nat^Sb(s) [12]: ^120m^Sb [8–10]: ^nat^Sb(m) [10]: Ga	[0.1–12]: ^117m^Sn [0.01–10]: Al, Ba, Bi, Ce, Cs, Co, Cu, Er, Eu, Hf, La, Pb, Lu, Mo, Nd, Ni, Nb, Re, Rb, Sc, Ag, Sr, Th, ^nat^Sn(m), U, Yb, Y, Zn, Zr [0.01–0.5; 6–8]: Tl [0.01–6]: ^nat^Sb(m) [0.01–3]: Ga [0.1]: ^120m^Sb
TK401-71	[10–12]: ^nat^Sb(s), ^120m^Sb [10]: ^nat^Sb(m), Ga	[0.1–12]: ^117m^Sn [0.01–10]: Al, Ba, Bi, Ce, Cs, Co, Cu, Er, Eu, Hf, La, Pb, Lu, Mo, Nd, Ni, Nb, Re, Rb, Sc, Ag, Sr, Th, ^nat^Sn(m), U, Yb, Y, Zn, Zr [0.01–0.5; 6–8]: Tl [0.01–6]: ^nat^Sb(m) [6]: ^nat^Sb(s) [0.01–3]: Ga [0.1–3]: ^120m^Sb
CG71 support only	[6–10]: ^nat^Sb(m), ^nat^Sb(s), Ga [1–10]: Tl	[0.01–10]: Al, Ba, Bi, Ce, Cs, Co, Cu, Er, Eu, Hf, La, Pb, Lu, Nd, Ni, Rb, Sc, Ag, Sr, Th, U, Yb, Y, Zn, Zr [0.01–1; 8–10]: ^nat^Sn(m) [6–10]: Re [0.01–6]: Nb [0.01–3]: ^nat^Sb(m), Ga, Mo [0.01–0.5]: Tl
DBE-300	[12]: ^120m^Sb [10]: ^nat^Sb(m)	[0.1–12]: ^117m^Sn [0.01–10]: Al, Ba, Bi, Ce, Cs, Co, Cu, Er, Eu, Hf, La, Pb, Lu, Mo, Nd, Ni, Nb, Re, Rb, Sc, Ag, Sr, Tl, Th, ^nat^Sn(m), U, Yb, Y, Zn, Zr [0.01–8]: ^nat^Sb(m), ^120m^Sb, Ga
DPE-300	[12]: ^120m^Sb [10]: ^nat^Sb(m), ^nat^Sb(s)	[0.1–12]: ^117m^Sn [0.01–10]: Al, Ba, Bi, Ce, Cs, Co, Cu, Er, Eu, Hf, La, Pb, Lu, Mo, Nd, Ni, Nb, Re, Rb, Sc, Ag, Sr, Tl, Th, ^nat^Sn(m), U, Yb, Y, Zn, Zr [0.01–6; 10]: Ga [0.01–8]: ^nat^Sb(m) [0.1–6]: ^120m^Sb [6]: ^nat^Sb(s)
DOE-300	[10–12]: ^120m^Sb [10]: ^nat^Sb(m)	[0.1–12]: ^117m^Sn [0.01–10]: Al, Ba, Bi, Ce, Cs, Co, Cu, Er, Eu, Ga, Hf, La, Pb, Lu, Mo, Nd, Ni, Nb, Re, Rb, Sc, Ag, Sr, Tl, Th, ^nat^Sn(m), U, Yb, Y, Zn, Zr [0.01–8]: ^nat^Sb(m) [6]: ^nat^Sb(s) [0.1–0.5; 2–3]: ^120m^Sb
TK401-300	-	[0.1–12]: ^120m^Sb, ^117m^Sn [6–12]: ^nat^Sb(s) [0.01–10]: ^nat^Sb(m), Ba, Bi, Ce, Cs, Co, Cu, Er, Eu, Ga, Hf, La, Pb, Lu, Mo, Nd, Ni, Nb, Re, Rb, Sc, Ag, Sr, Tl, Th, ^nat^Sn(m), U, Yb, Y, Zn, Zr [0.01–0.5; 2–10]: Al
CG300 support only	-	[0.01–10]: Al, ^nat^Sb(m), Ba, Bi, Ce, Cs, Co, Cu, Er, Eu, Ga, Hf, La, Pb, Lu, Mo, Nd, Ni, Nb, Re, Rb, Sc, Ag, Sr, Tl, Th, ^nat^Sn(m), U, Yb, Y, Zn, Zr

**Fig. 4:**
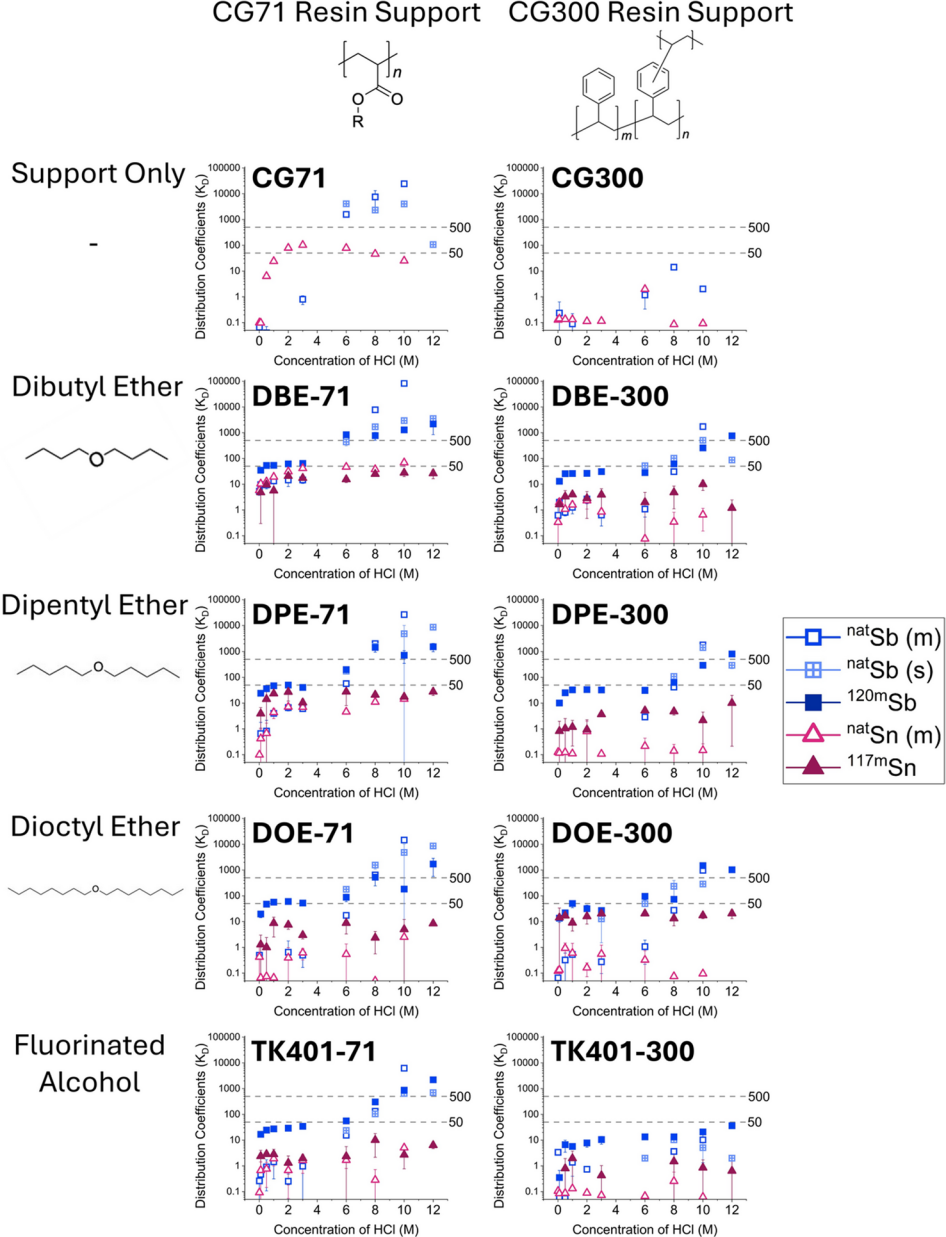
Distribution coefficients (K_D_) of antimony(V) and tin(IV) on the synthesized resins and their unfunctionalized supports (1 µg ^nat^Sb/^nat^Sn, ICP-MS, *n* = 3, OR 10^0^ pg ^120m^Sb, 10^2^ fg ^117m^Sn, 10^0^ mg ^nat^Sn, γ-spectrometry, *n* ≥ 3). Sufficient separation requires good retention of antimony (K_D_ >500) and no retention of tin (K_D_ < 50), denoted by the horizontal dotted lines in the graphs. Where K_D_ values were found to be < 0.1 they are displayed as 0.1 ± 0.0

Based on the obtained K_D_ values, Sb(V)/Sn(IV) separations can be achieved using 7 of the synthesized resins (DBE-71, DPE-71, DOE-71, TK401-71, DBE-300, DPE-300, DOE-300), as well as by using just the CG71 resin support (Table 4; Fig. 4). Four of the resins (DBE-71, DPE-71, DOE-71, TK401-71) and the CG71 resin support were also able to retain gallium and thallium to establish a suitable separation from the other elements listed.

**Fig. 5:**
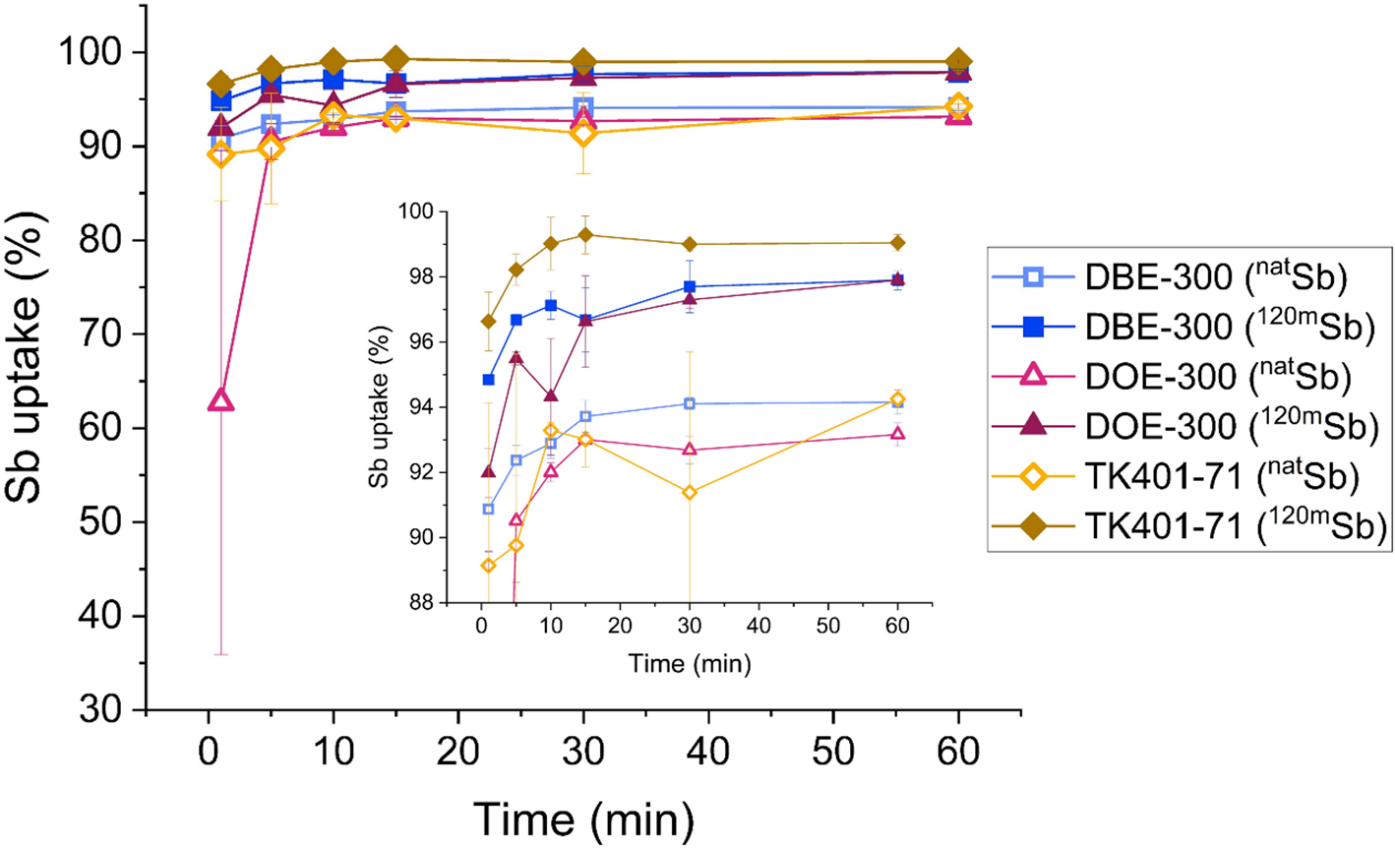
Rate of ^nat^Sb(V) and ^120m^Sb(V) uptake on DBE-300, DOE-300 and TK401-71 resins (1 µg ^nat^Sb, ICP-MS, *n* = 3, **OR** 10^0^ pg ^120m^Sb, 10^2^ fg ^117m^Sn, 10^0^ mg ^nat^Sn, γ-spectrometry, *n* = 3)

The rate of Sb uptake on some of the suitable resins was tested using stable (^nat^Sb, 1 µg) and radioactive (^120m^Sb, 1 pg) tracers to evaluate the duration of the load step necessary for a dynamic column chromatography separation (Fig. 5). For all three tested resins, 15 min of interaction with the resin were necessary to achieve maximum adherence to the resin. Radiochemical experiments with ^120m^Sb showed comparatively higher and faster uptake than ^nat^Sb, which may be attributed to the large differences in concentration of Sb used in each experiment or systematic errors arising from the dilution corrections required for ICP-MS measurements.

### Extraction chromatography

Following the encouraging results of the K_D_ experiments, the resins were put to the test in a practical setting, evaluating the elution profiles of Sb and Sn in a conventional dynamic column chromatography setup. A range of other elements of potential interest or potential contamination, such as iron, that may often be present in the acidic environment of radiochemical laboratories were also investigated and the elution profiles of these elements can be seen in Supplementary Information Figures S1 – S9.

**Fig. 6:**
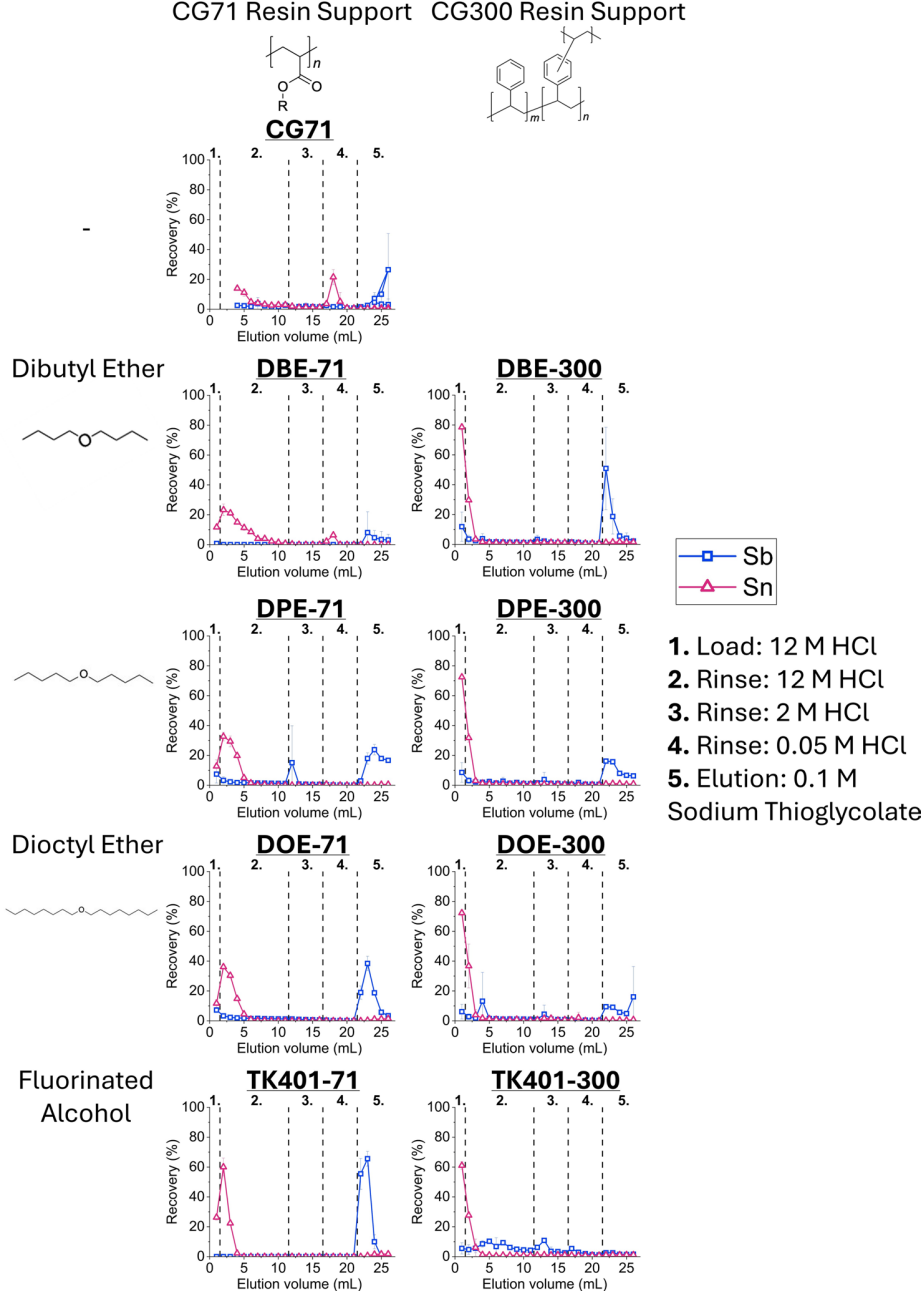
Elution curves of stable antimony and tin on the synthesized resins and the unfunctionalized CG71 support (1 µg ^nat^Sb/^nat^Sn, multi-element standard, 1 mL column volume, ICP-MS, *n* = 3). Note that the species in HCl are Sb(V) and Sn(IV) chlorides, but sodium thioglycolate is a good reducing and chelating agent for Sb, recovering it as Sb(III) thioglycolate complex. See Supplementary Information Figures S1-S9 for the elution profiles of other elements under the same elution conditions

Based on the results of the K_D_ experiments, a load matrix and wash steps using 12 M HCl were chosen to ensure the retention of Sb(V). Since Sb showed no adherence to the resin at low HCl molarities in the K_D_ experiments, the elution steps of in 2 M and 0.05 M were tested, followed by third elution step in a known chelating and reducing agent of Sb – 0.1 M sodium thioglycolate – complexing thiophilic Sb(III). Figure 6 shows a summary of the Sb and Sn elution profiles under these conditions. Compared to the resins utilizing the CG71 resin support material, which resulted in more wash steps required to elute Sn(IV), the resins utilizing the CG300 support showed a much sharper, consistent Sn elution curve. Retention of Sb for all the resins expected to retain Sb was near-quantitative. However, once the Sb was retained on the column, the subsequent elution was challenging. Despite having low K_D_ values at low acid concentrations, Sb would not elute in neither 2 M, nor 0.05 M HCl, requiring the chelating agent sodium thioglycolate to be stripped from the column. Furthermore, despite the use of thioglycolate as a chelating and reducing agent, the subsequent recovery of Sb was subpar for most resins, with the worst recovery being just 19% Sb from DBE-71 and middling recoveries between 45 and 85% for DPE and DOE resins warranting further investigation in elution conditions.

**Fig. 7:**
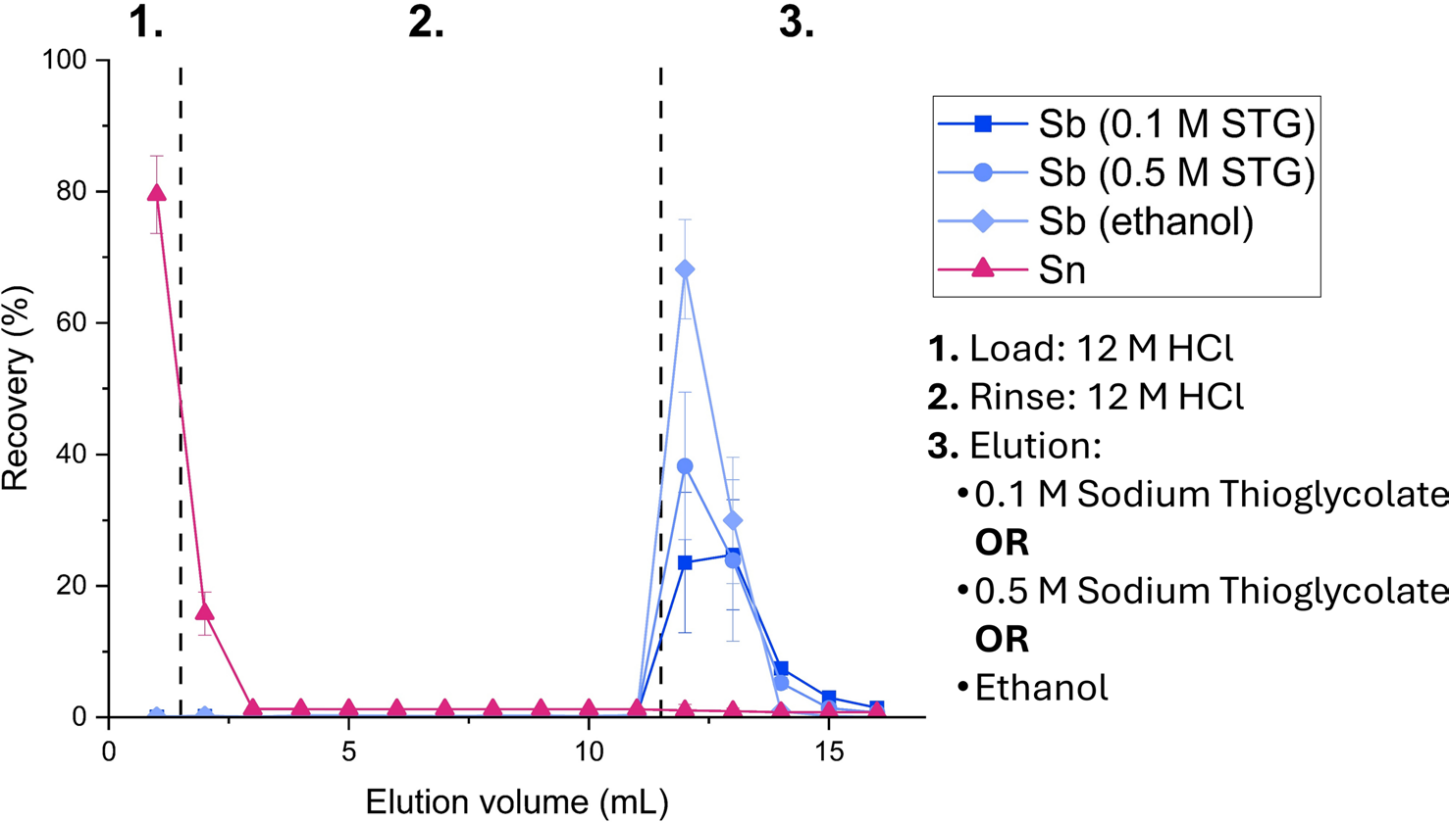
Elution curves of radioantimony and radiotin on the DBE-300 resin using sodium thioglycolate (STG) and ethanol as eluants (1 MBq or 10^2^ pg ^120m^Sb, 30 kBq or 10^1^ pg ^117m^Sn, 10^0^ mg ^nat^Sn, 1 mL column volume, γ-spectrometry, *n* = 3). Note that the species in HCl are Sb(V) and Sn(IV) chlorides, while in sodium thioglycolate a Sb(III) thioglycolate complex is formed. The Sb species in ethanol is not conclusive but is expected to be Sb(V) chloride in the absence of Sn

Based on the stable elution curves (Fig. 6) DBE-300 exhibited the best Sb(III) recovery (82% in 5 mL 0.1 M sodium thioglycolate) out of all CG300 functionalized resins while showing an ideal Sn elution profile (100% in 2 mL 12 M HCl). Therefore, this resin was chosen to be investigated further for radiochemical applications (Fig. 7). Since the recovery of Sb was not quantitative, the intermediate elution steps in dilute HCl (2 M HCl and 0.05 M HCl) were omitted out of worry for Sb hydrolysis (Neumann 1954). To potentially improve the elution of Sb, a higher concentration of sodium thioglycolate (0.5 M) as well as ethanol were investigated. The ^117m^Sn elution profile was found to be comparable to the ^nat^Sn, recovering > 95% Sn in just 2 mL of 12 M HCl. In contrast, the elution profile of ^120m^Sb at 60% Sb recovered in 5 mL 0.1 M sodium thioglycolate was found to be much lower than for ^nat^Sb (82%). Increasing the concentration of sodium thioglycolate to 0.5 M slightly improved the recovery of Sb(III) to 69%. Ultimately, the best eluant was found to be ethanol, recovering 99% of Sb in 5 mL of ethanol, or 98% in just 2 mL of ethanol, likely as Sb(V) chloride.

## Discussion

### Synthesis and characterization

The resins evaluated in this work are simple to synthesize and abide by commercial standards according to the TOC stability for aqueous media and resin capacity tests. The high resin stability is especially beneficial for radiochemical separations, where the presence of competing functional groups can potentially interfere with chelation and re-use of enriched target material. The presence of trace dibutyl ether in the ethanol elute fraction indicates that the resins are not stable in ethanol, which is to be expected as any extractant soluble in ethanol will be removed from the resin at least partially. However, due to the volatility of dibutyl ether (and ethanol), this contamination may be easily minimized by evaporating the solvents. Further work quantifying any residual solvents after such evaporation must be carried out to confirm the viability of these resins for preclinical or clinical use. Similar techniques have been successfully used for other applications, e.g., actinide removal from actinide resin (AC resin) using isopropanol (Barrett et al. 2021). Furthermore, the resin capacity is very high. For radiochemical applications, the tens to hundreds of milligrams capacity per gram of resin is many magnitudes higher than required for nanogram to microgram levels of radionuclides produced. This means that only a small mass of resin is required for an efficient separation, making it easier for recovery of the radionuclide in a small volume and potential future automation.

### Distribution coefficients

K_D_ experiments showed good viability for the separation of Sb(V)/Sn(IV) on many of the resins (DBE-71, DPE-71, DOE-71, TK401-71, DBE-300, DPE-300, DOE-300) and the CG71 resin support. For Sb to be retained on the resin, an oxidation state of Sb(V) and a high concentration of HCl is required. This is to be expected as Sb readily undergoes hydrolysis in HCl molarities up to as high as 9 M HCl (Neumann 1954).

Besides Sb, other elements like Ga and Tl are also very well retained on the CG71 support and its corresponding functionalized resins. This may be of potential interest to other radionuclides used in radiopharmaceuticals, such as ^67^Ga, ^68^Ga, and ^201^Tl. Most commonly ^68^Ga is recovered from ^68^Ge generators, although it may also be produced directly from ^68^Zn. Although the Ge K_D_ values were not determined in this study, the Ga and Zn K_D_ suggest suitability for separation between ^68^Ga/^68^Zn. In fact, this resin support has already found application for this purpose, showing retention of both ^68^Ga and ^68^Ge (Zhernosekov et al. 2018; Heerden et al. 2020; Naidoo and Walt 2001). Furthermore, ^201^Tl is typically acquired from ^201^Pb generators, which is another elemental separation that may be achieved on these resins. Although out of scope for this paper we encourage further investigation of these resins for other applications.

The mechanism behind the retention of Sb is not clear. In the case where CG71 resin support is used, a contribution from the support is evidenced by the retention of Sb on the resin support at high HCl concentrations when no functional group has been added. Moreover, other elements are also retained on the CG71 resin support at these conditions. We hypothesize that the CG71 - an aliphatic acrylic ester polymer is undergoing acid catalyzed hydrolysis of the ester groups and becoming protonated, functionally acting as a weak anion exchange resin. Although this provides another mechanism of retention, potentially increasing the capacity of the ether or alcohol functionalized CG71 resin, the number of functional sites would be very difficult, if not impossible to quantify, raising concerns about reproducibility. The retention of, and weak interactions, with elements other than Sb may also be troublesome for some applications, increasing competition for the functional sites available. However, for applications in nuclear medicine, we do not foresee any considerable presence of Ga or Tl that would decrease the efficacy of these resins. In the case of the inert CG300 divinylbenzene copolymer support, no elements were retained on the resin support itself as expected. For the ether functionalized resins with the CG300 support (DBE-300, DPE-300, DOE-300) only Sb is selectively retained. Since Sb forms SbCl_6_^−^ in highly acidic HCl media, we hypothesize that it may act as a weaker analogue to the strongest known superacid, fluoroantimonic acid. Where fluoroantimonic acid can protonate organic compounds, we suggest that the chloride analogue may be able to form weak interactions with the carbon chains in the ether groups that the other elements are not able to, creating a truly elementally selective resin.

### Extraction chromatography

Unexpected elution behaviour was observed when investigating the fluorinated alcohol (TK401) functionalized resins (TK401-71 and TK401-300). The CG71 resin support was found to act as a weak anion exchange resin at high HCl concentrations, so it is no surprise that the TK401-71 resin is able to retain Sb. What is interesting, however, is the unexpected elution behaviour of Sb on the TK401-300 resin. Based on the K_D_ values, Sb should not be retained on the resin in any capacity but was not so easily eluted from the column, indicating some form of weak interaction between the resin and the Sb. Furthermore, if the fluorinated alcohol functional groups had no effect on the elution profile, the one acquired for the CG71 resin support and one for the TK401-71 should look the same, but there is a clear improvement upon adding the fluorinated alcohol functional group.

The ether resins developed and characterized in this study offer new pathways for the acquisition of ^119^Sb in a matrix and form compatible with subsequent radiopharmaceutical development steps. Evaluating the ^nat^Sb elution profiles for all of the resins, the recovery of ^nat^Sb was overall middling, resulting in considerable amounts of the radionuclide being irreversibly bound onto the column. The necessity of the sodium thioglycolate chelating agent for elution also raises concerns, as this chemical form would require a very strong chelator that is able to successfully trans-chelate the Sb-thioglycolate complex. Furthermore, even if trans-chelation can be easily achieved, sodium thioglycolate remaining in solution would not be safe for clinical or even pre-clinical in vivo applications, requiring additional purification to remove any sodium thioglycolate from the solution. Although it is not clear if the poor recovery of Sb can be partially attributed to hydrolysis, due to the high sensitivity of Sb to hydrolysis intermediate wash/elution steps in dilute HCl should also be avoided. Elution in ethanol yielded near-quantitative results for recovery of ^1xx^Sb, likely as Sb(V) chloride. The high recovery discrepancy between ethanol and aqueous eluents such as sodium thioglycolate solutions may be attributed to the lack of resin stability in ethanol, eluting all of Sb alongside the dibutyl ether functional group. Recovery in anhydrous ethanol also eliminates the concern of Sb hydrolysis and provides a chelation matrix without any competitors. Furthermore, in low doses ethanol is safe for injection in humans and animals alike, and in cases where ethanol may not be appropriate for further radiopharmaceutical synthesis steps it can be easily removed by evaporation before or after radiolabeling and the Sb reconstituted in another matrix of choice. For the direct production route from enriched ^119^Sn to be economically viable, the enriched target material must be recovered after the separation step and re-used for several irradiations. For this reason, it is important to consider the Sn elution profile, in this regard the resins utilizing the CG300 support material are preferred. Their ability to quantitatively recover the Sn in a volume of just 2 mL is encouraging in the efforts to develop a compatible ^119^Sn recycling method by avoiding any intermediate concentration steps and loss of the expensive material during purification. Further experiments for scaling up the separation using larger masses of ^nat^Sn targets (10^1^ – 10^2^ mg) and subsequently higher amounts of ^1xx^Sb (up to 1 GBq) than reported here (10^0^ mg ^nat^Sn, 1 MBq ^120m^Sb) are currently underway to evaluate the optimal column geometry for larger target masses. At this time, we do not foresee issues with radiolysis of the resin, considering that for applications in nuclear medicine, selective production of ^119^Sb would ensure that only emissions < 30 keV (AEs, CEs, X-rays, and γ-rays) are present, which are unlikely to result in significant radiation damage to the resin.

## Conclusions

We report the synthesis and characterization of six new ether-based resins and two alcohol-based resins. All of the synthesized resins were shown to be stable in acidic and aqueous media. All ether-based resins and one of the alcohol-based resins (TK401-71) showed a high capacity for retaining Sb. Although the resin support material is intended to be inert with respect to retaining elements, one of the investigated resin support materials, CG71, was found to interact with several elements (Sb, Ga, Tl) at high concentrations of HCl. Fluorinated alcohol as the functional group exhibited weak interactions with Sb but was ultimately found to be less optimal than the ether resins (insufficient retention in the case of TK401-300, or broad Sn elution curves/retention of other elements on the TK401-71).

Ultimately, DBE-300 was found to be the most suitable resin for ^119^Sb/^119^Sn separations. Using this resin, we were able to quantitatively recover Sn(IV) in a small volume of HCl (2 mL of 12 M HCl) making it suitable for recycling of enriched target material. Most importantly, near-quantitative (98%) recovery of Sb was acquired in just 2 mL of ethanol. This ensures an anhydrous environment and therefore prevention of ^119^Sb hydrolysis and ease of concentration due to the volatility of ethanol. This resin could be well suited for applications pre-clinically or even clinically due to the low toxicological concern posed by ethanol, although further quantification of residual extractant must be conducted to validate this claim as additional solvent evaporation or additional purification steps may be needed.

## Supplementary Information

Below is the link to the electronic supplementary material.

Supplementary Material 1

## Data Availability

The datasets generated during and/or analysed during the current study are available from the corresponding author on reasonable request.

## Abbreviations

AEs: Auger electrons
CEs: Conversion electrons
EC: Electron capture
EDTA: Ethylenediaminetetraacetic acid
HPGe: High purity germanium
ICP-MS: Inductively coupled plasma–mass spectrometry
LET: Linear energy transfer
NMR: Nuclear magnetic resonance
NPOC: Non–purgeable organic carbon
PET: Positron emission tomography
RPT: Radiopharmaceutical therapy
STG: Sodium thioglycolate
SPECT: Single photon emission computed tomography
TOC: Total organic carbon
