# Supplementary material for: Developing ether and alcohol based extraction chromatography resins for purification of antimony-119 in nuclear medicine
Source: EJNMMI Radiopharm Chem. 2025 Aug 13;10:53. doi: 10.1186/s41181-025-00379-y (PMC12350969; doi:10.1186/s41181-025-00379-y)
Supplement: Supplementary file 1 — Supplementary Material 1 [file 41181_2025_379_MOESM1_ESM.docx]

Supplementary Information for:

**Developing ether and alcohol based extraction chromatography resins for purification of antimony-119 in nuclear medicine**

Aivija Grundmane^a,b^, Illarion Dovhyi^c^, Lauren Aburto-Kung^a^, Steffen Happel^c^, Caterina Ramogida^a,b^ and Valery Radchenko^b,d^

^a^ Simon Fraser University, Department of Chemistry, 8888 University Dr W, Burnaby, BC, V5A 1S6, Canada, aivija_grundmane@sfu.ca, caterina_ramogida@sfu.ca.

^b^ TRIUMF, Life Sciences Division, 4004 Wesbrook Mall, Vancouver, BC, V6T 2A3, Canada. vradchenko@triumf.ca

^b^ TrisKem International, 3 Rue des Champs Géons ZAC de, L'Éperon, 35170, Bruz, France.

^d^ University of British Columbia, Department of Chemistry, Vancouver, BC, V6T 1Z4, Canada.

**Table S1.** Radionuclides produced from proton bombardment of ^nat^Sn and their predicted and measured activities decay corrected for the end of bombardment time.

| Radionuclide^[a]^ | Half-life (h) | Decay^[b]^ | Predicted Activity (Electroplated) (MBq)^[c]^ | Measured Activity (Electroplated)  (n = 3, MBq)^[d]^ |
| --- | --- | --- | --- | --- |
| ^115^Sb | 0.535 | 100% ε | 161 | ^[e]^ |
| ^116^Sb | 0.263 | 100% ε | 9250 | ^[e]^ |
| ^116m^Sb | 1.01 | 100% ε | 208 | ^[e]^ |
| ^117^Sb | 2.80 | 100% β^+^ | 1470 | 700 ± 200 |
| ^117m^Sn | 336 | 100% IT | 0.177 | ^[e]^ |
| ^118^Sb | 0.0600 | 100% ε | 17900 | ^[e]^ |
| ^118m^Sb | 5.00 | 100% ε | 87.6 | 70 ± 20 |
| ^119^Sb | 38.2 | 100% EC | 281 | 280 ± 60 |
| ^120^Sb | 0.265 | 100% β^+^ | 12300 | ^[e]^ |
| ^120m^Sb | 138 | 100% ε | 7.42 | 3 ± 1 |
| ^122^Sb | 65.4 | 97.6% β^-^  2.41% ε | 8.74 | 6 ± 2 |
| ^124^Sb | 1440 | 100% β^-^ | 0.142 | 0.18 ± 0.04 |
| ^124m^Sb | 0.337 | 100% IT | 136 | ^[e]^ |

[a] Only radioantimony and radiotin nuclides with a half-life longer than 10 min (or those generated from a meta-stable state with a half-life longer than 10 min) resulting in a predicted activity of at least 0.1 MBq at the highest irradiation conditions are reported here.

[b] β^-^ – beta decay, EC – electron capture, β^+^ – positron, ε – EC and β^+^, IT – isomeric transition.

[c] Calculated using IAEA Medical Isotope Browser, IAEA and TENDL cross sections. Irradiation of a 100 mg/cm^2^ ^nat^Sn electroplated on Ag backing using 13 MeV protons, 30 µA beam current for 1 hour.

[d] Irradiation of 100 ± 1 mg/cm^2^ (n = 3) ^nat^Sn electroplated on Ag backing using 13 MeV protons, 30 µA beam current for 1 hour.

[e] Radionuclide not measured experimentally.

**Table S2**. Mass of the resin used depending on the volume of the chromatography reservoir.

| Resin Name | Mass of resin in 1 mL reservoir (mg ± 5 mg) | Mass of resin in 2 mL reservoir (mg ± 5 mg) |
| --- | --- | --- |
| DBE-71 | 300 | 500 |
| DPE-71 | 300 | 600 |
| DOE-71 | 300 | 600 |
| TK401-71 | 350 | 700 |
| CG71 support only | 300 | 650 |
| DBE-300 | 200 | 400 |
| DPE-300 | 250 | 500 |
| DOE-300 | 250 | 550 |
| TK401-300 | 300 | 550 |

**Table S3**. Layering of aqueous phase and resin in a range of concentrations/densities of hydrochloric acid. ↑ – resin floated, ↓ – resin sank, ↕ – no clear layering.

| Resin name | Resin layering in different concentrations/densities of hydrochloric acid | | | | | | | | | |
| --- | --- | --- | --- | --- | --- | --- | --- | --- | --- | --- |
|  | 0.01 M  (1.00 g/cm^3^) | 0.1 M  (1.00 g/cm^3^) | 0.5 M  (1.01 g/cm^3^) | 1 M  (1.02 g/cm^3^) | 2 M  (1.03 g/cm^3^) | 3 M  (1.07 g/cm^3^) | 6 M  (1.10 g/cm^3^) | 8 M  (1.13 g/cm^3^) | 10 M  (1.16 g/cm^3^) | 12 M  (1.18 g/cm^3^) |
| DBE-71 | ↑ | ↑ | ↕ | ↕ | ↕ | ↕ | ↓ | ↓ | ↓ | ↓ |
| DPE-71 | ↑ | ↑ | ↑ | ↑ | ↑ | ↑ | ↑ | ↑ | ↑ | ↑ |
| DOE-71 | ↑ | ↑ | ↑ | ↑ | ↑ | ↑ | ↑ | ↑ | ↑ | ↑ |
| TK401-71 | ↑ | ↑ | ↑ | ↑ | ↑ | ↑ | ↕ | ↓ | ↓ | ↓ |
| CG71 support only | ↓ | ↓ | ↓ | ↓ | ↓ | ↓ | ↓ | ↓ | ↓ | ↓ |
| DBE-300 | ↑ | ↑ | ↑ | ↑ | ↑ | ↑ | ↑ | ↑ | ↑ | ↑ |
| DPE-300 | ↑ | ↑ | ↑ | ↑ | ↑ | ↑ | ↑ | ↑ | ↑ | ↑ |
| DOE-300 | ↑ | ↑ | ↑ | ↑ | ↑ | ↑ | ↑ | ↑ | ↑ | ↑ |
| TK401-300 | ↑ | ↑ | ↑ | ↑ | ↑ | ↑ | ↑ | ↑ | ↑ | ↑ |
| CG300 support only | ↑ | ↑ | ↑ | ↑ | ↑ | ↑ | ↑ | ↑ | ↑ |  |

**Table S4**. Distribution coefficient (K_D_) values for DBE-71 resin.

| Element^[a]^ | Distribution coefficients (K_D_) in hydrochloric acid (K_D_ ± SD, n = 3 unless otherwise noted) | | | | | | | | | |
| --- | --- | --- | --- | --- | --- | --- | --- | --- | --- | --- |
|  | 0.01 M | 0.1 M | 0.5 M | 1 M | 2 M | 3 M | 6 M | 8 M | 10 M | 12 M |
| Aluminium (Al) | 21 ± 6 | 12 ± 3 | 12 ± 4 | 31 ± 16 | 30 ± 30 | 18 ± 4 | 17 ± 13 | 22 ± 13 | 50 ± 40 |  |
| Antimony (Sb) | 5.3 ± 1.6 | 10 ± 2 | 9 ± 3 | 13 ± 3 | 15 ± 6 | 15 ± 4 | 540 ± 20 | 7700 ± 700 | 82000 ± 11000 |  |
| Antimony (Sb, single-element standard) |  |  |  |  |  |  | 440 ± 130 | 1700 ± 200 | 2950 ± 170 | 3600 ± 900 |
| Antimony (^120m^Sb) |  | 35 ± 4 | 54 ± 2 | 54 ± 7 | 62 ± 4 | 64 ± 4 | 840 ± 20 | 800 ± 300 (n = 8) | 1300 ± 200 | 2200 ± 1300 |
| Barium (Ba) | 5.1 ± 1.5 | 10 ± 2 | 10 ± 3 | 13 ± 3 | 14 ± 7 | 15 ± 4 | 18.8 ± 1.9 | 13 ± 2 | 37 ± 14 |  |
| Bismuth (Bi) | 1.9 ± 0.3 | 10 ± 3 | 11 ± 5 | 14.4 ± 0.3 | 15 ± 6 | 15.2 ± 1.7 | 19 ± 4 | 14 ± 3 | 37 ± 12 |  |
| Cerium (Ce) | 4 ± 3 | 14 ± 3 | 9.3 ± 1.3 | 9 ± 8 | 13 ± 7 | 13 ± 6 | 16 ± 4 | 15 ± 4 | 31 ± 11 |  |
| Cesium (Cs) | 6.3 ± 1.4 | 11 ± 2 | 11 ± 3 | 14 ± 2 | 15 ± 8 | 15 ± 4 | 19.1 ± 1.7 | 14 ± 3 | 38 ± 14 |  |
| Cobalt (Co) | 7.5 ± 1.9 | 11 ± 3 | 11 ± 3 | 17 ± 3 | 16 ± 8 | 15 ± 4 | 20.9 ± 1.8 | 18 ± 3 | 45 ± 17 |  |
| Copper (Cu) | 7 ± 2 | 11 ± 2 | 12 ± 3 | 16 ± 3 | 17 ± 7 | 15 ± 5 | 21 ± 3 | 16 ± 2 | 44 ± 18 |  |
| Erbium (Er) | 6.0 ± 1.4 | 9 ± 2 | 9 ± 3 | 13 ± 3 | 15 ± 7 | 16 ± 4 | 19 ± 2 | 14 ± 3 | 37 ± 14 |  |
| Europium (Eu) | 5.6 ± 1.4 | 8 ± 2 | 8.0 ± 0.8 | 13 ± 3 | 15 ± 7 | 16.7 ± 0.3 | 17.9 ± 1.9 | 13 ± 2 | 35 ± 13 |  |
| Gallium (Ga) | 7.3 ± 1.7 | 11 ± 2 | 10.3 ± 1.0 | 16 ± 4 | 17 ± 7 | 22 ± 6 | 1430 ± 110 | 1290 ± 120 | 2600 ± 800 |  |
| Hafnium (Hf) | 4.4 ± 1.6 | 9 ± 2 | 9.9 ± 1.9 | 14 ± 3 | 14 ± 6 | 14 ± 4 | 16 ± 2 | 12 ± 2 | 33 ± 12 |  |
| Lanthanum (La) | 6.0 ± 1.8 | 9 ± 2 | 10 ± 3 | 13 ± 3 | 14 ± 6 | 14 ± 4 | 15.8 ± 1.7 | 13 ± 2 | 33 ± 13 |  |
| Lead (Pb) | 2.6 ± 1.9 | 8 ± 3 | 9 ± 5 | 14.6 ± 1.6 | 14 ± 4 | 14.7 ± 1.7 | 20 ± 6 | 14 ± 8 | 43 ± 11 |  |
| Lutetium (Lu) | 5.1 ± 1.7 | 10 ± 2 | 10 ± 3 | 14 ± 3 | 14 ± 7 | 14 ± 4 | 16.9 ± 1.7 | 13 ± 2 | 34 ± 14 |  |
| Molybdenum (Mo) | 5.6 ± 1.7 | 10.9 ± 1.7 | 10 ± 3 | 16 ± 4 | 20 ± 9 | 27 ± 5 | 90 ± 6 | 91 ± 14 | 180 ± 50 |  |
| Neodymium (Nd) | 4 ± 3 | 10 ± 2 | 10 ± 3 | 14 ± 3 | 13 ± 7 | 13 ± 5 | 15 ± 7 | 12 ± 2 | 32 ± 11 |  |
| Nickel (Ni) | 8 ± 2 | 12 ± 3 | 13 ± 4 | 17 ± 3 | 18 ± 7 | 20 ± 6 | 22 ± 2 | 19 ± 3 | 46 ± 19 |  |
| Niobium (Nb) | 7.6 ± 1.5 | 13 ± 2 | 10 ± 3 | 17 ± 4 | 18 ± 8 | 15 ± 4 | 26 ± 3 | 69 ± 11 | 390 ± 110 |  |
| Rhenium (Re) | 13 ± 2 | 19 ± 3 | 22 ± 3 | 28 ± 5 | 30 ± 9 | 31 ± 5 | 26 ± 3 | 21 ± 3 | 41 ± 15 |  |
| Rubidium (Rb) | 6 ± 2 | 11 ± 2 | 13 ± 3 | 15 ± 3 | 16 ± 7 | 15 ± 4 | 21 ± 2 | 15 ± 3 | 42 ± 16 |  |
| Scandium (Sc) | 6.7 ± 1.8 | 10.6 ± 1.9 | 11 ± 3 | 17 ± 2 | 16 ± 7 | 16 ± 4 | 21 ± 3 | 16 ± 2 | 42 ± 17 |  |
| Silver (Ag) | 4 ± 3 | 21.0 ± 1.6 | 13 ± 4 | 17 ± 3 | 18 ± 8 | 17 ± 4 | 20.7 ± 1.3 | 15 ± 3 | 40 ± 15 |  |
| Strontium (Sr) | 6 ± 2 | 10 ± 3 | 12 ± 3 | 16 ± 3 | 17 ± 8 | 15 ± 5 | 20 ± 2 | 14 ± 3 | 41 ± 16 |  |
| Thallium (Tl) | 5.3 ± 1.8 | 13.7 ± 1.5 | 90 ± 10 | 7100 ± 500 | 10000 ± 2000 | 6600 ± 400 | 3000 ± 1000 | 2200 ± 600 | 2500 ± 500 |  |
| Thorium (Th) | 4.1 ± 1.7 | 7.6 ± 1.4 | 9 ± 2 | 12.8 ± 1.9 | 13 ± 5 | 12 ± 3 | 15 ± 3 | 12 ± 3 | 32 ± 10 |  |
| Tin (Sn) | 5.8 ± 1.8 | 10.5 ± 1.5 | 13 ± 3 | 19 ± 4 | 31 ± 10 | 42 ± 6 | 47 ± 5 | 38 ± 4 | 70 ± 20 |  |
| Tin (^117m^Sn) |  | 5 ± 5 | 10 ± 2 | 6 ± 7 | 21 ± 6 | 17 ± 5 | 15 ± 4 | 25 ± 3 | 28 ± 8 | 26 ± 10 |
| Uranium (U) | 4.8 ± 1.9 | 8 ± 2 | 10 ± 3 | 14 ± 3 | 14 ± 7 | 14 ± 4 | 17 ± 2 | 14.9 ± 1.6 | 42 ± 15 |  |
| Ytterbium (Yb) | 6.0 ± 1.8 | 9 ± 3 | 10 ± 3 | 14 ± 3 | 15 ± 7 | 15 ± 5 | 18 ± 2 | 14 ± 2 | 37 ± 14 |  |
| Yttrium (Y) | 6.7 ± 1.3 | 11 ± 2 | 11 ± 3 | 16 ± 3 | 17 ± 7 | 16 ± 5 | 20.7 ± 1.9 | 14 ± 3 | 40 ± 15 |  |
| Zinc (Zn) | 4.9 ± 1.9 | 9.1 ± 1.8 | 9.7 ± 1.9 | 15 ± 3 | 15 ± 7 | 15 ± 4 | 20.1 ± 1.9 | 15 ± 2 | 38 ± 14 |  |
| Zirconium (Zr) | 5.9 ± 1.5 | 10.0 ± 1.8 | 11 ± 3 | 14 ± 3 | 16 ± 7 | 13 ± 4 | 18.8 ± 1.8 | 13 ± 3 | 38 ± 13 |  |

^[a]^Unless noted otherwise, all K_D_ values were acquired using stable elements from a multi-element standard.

**Table S5**. Distribution coefficient (K_D_) values for DPE-71 resin.

| Element^[a]^ | Distribution coefficients (K_D_) in hydrochloric acid (K_D_ ± SD, n = 3) | | | | | | | | | |
| --- | --- | --- | --- | --- | --- | --- | --- | --- | --- | --- |
|  | 0.01 M | 0.1 M | 0.5 M | 1 M | 2 M | 3 M | 6 M | 8 M | 10 M | 12 M |
| Aluminium (Al) | 2.7 ± 1.4 | 1.2 ± 0.4 | 1.8 ± 1.3 | 7.2 ± 0.7 | 12 ± 4 | 8 ± 6 | 4 ± 4 | 17.5 ± 1.2 | 13 ± 2 |  |
| Antimony (Sb) | 2.74×10^-3^ ± 9×10^-5^ | 0.7 ± 1.1 | 0.8 ± 1.3 | 4.0 ± 1.5 | 6.7 ± 1.8 | 6.1 ± 0.9 | 57 ± 3 | 2040 ± 150 | 26000 ± 2000 |  |
| Antimony (Sb, single-element standard) |  |  |  |  |  |  | 174 ± 14 | 1600 ± 500 | 5000 ± 5000 | 8600 ± 300 |
| Antimony (^120m^Sb) |  | 24.2 ± 1.5 | 36 ± 3 | 47 ± 5 | 50 ± 2 | 41 ± 5 | 200 ± 20 | 1500 ± 500 | 700 ± 400 | 1500 ± 500 |
| Barium (Ba) | 6.4×10^-3^ ± 2×10^-4^ | 0.4 ± 0.8 | 1.3 ± 1.2 | 3.6 ± 1.1 | 5.3 ± 1.3 | 6.9 ± 1.0 | 3.5 ± 0.5 | 8.2 ± 1.0 | 8 ± 2 |  |
| Bismuth (Bi) | 2.0 ± 0.6 | 0.50 ± 0.09 | 0.4 ± 0.4 | 1.3 ± 0.9 | 4 ± 4 | 3 ± 2 | 4.5 ± 1.3 | 6.9 ± 1.7 | 8.0 ± 1.6 |  |
| Cerium (Ce) | 1.05×10^-2^ ± 5×10^-4^ | 4 ± 2 | 1.4 ± 1.3 | 3.7 ± 1.4 | 5.6 ± 1.7 | 6.4 ± 0.5 | 4.5 ± 0.2 | 7.5 ± 1.7 | 7.7 ± 0.6 |  |
| Cesium (Cs) | 4.8×10^-3^ ± 2×10^-4^ | 0.5 ± 0.9 | 1.9 ± 1.4 | 4.2 ± 1.2 | 6.0 ± 1.3 | 6.4 ± 1.0 | 4.8 ± 1.3 | 8.3 ± 1.4 | 7.8 ± 1.0 |  |
| Cobalt (Co) | 4.86×10^-3^ ± 8×10^-5^ | 0.5 ± 0.9 | 2.0 ± 1.1 | 3.2 ± 1.8 | 8.5 ± 1.8 | 7.4 ± 0.9 | 5.8 ± 1.0 | 8.3 ± 0.8 | 9.2 ± 0.9 |  |
| Copper (Cu) | 4.9×10^-2^ ± 3×10^-3^ | 1.1 ± 1.7 | 2.0 ± 1.5 | 5.6 ± 1.4 | 8 ± 2 | 7 ± 3 | 5.2 ± 1.0 | 8.2 ± 1.3 | 8.5 ± 0.4 |  |
| Erbium (Er) | 3.8×10^-4^ ± 2×10^-5^ | 0.6 ± 1.1 | 1.5 ± 1.5 | 3.6 ± 1.4 | 6.5 ± 1.8 | 6.2 ± 0.2 | 4.2 ± 0.4 | 7.0 ± 1.8 | 7.9 ± 1.0 |  |
| Europium (Eu) | 1.45×10^-3^ ± 5×10^-5^ | 0.5 ± 0.9 | 2.4 ± 1.0 | 3.5 ± 1.0 | 5.6 ± 1.4 | 5.5 ± 0.9 | 4.3 ± 0.9 | 7.9 ± 1.4 | 7.2 ± 1.8 |  |
| Gallium (Ga) | 2.40×10^-3^ ± 1.1×10^-4^ | 0.6 ± 1.1 | 1.7 ± 1.4 | 4.2 ± 1.9 | 6 ± 2 | 8.0 ± 0.8 | 420 ± 20 | 1330 ± 30 | 1600 ± 90 |  |
| Hafnium (Hf) | 2.27×10^-2^ ± 1.1×10^-3^ | 0.9 ± 1.5 | 1.9 ± 1.2 | 2.6 ± 1.1 | 5 ± 2 | 6.9 ± 1.2 | 4.3 ± 1.2 | 6.6 ± 1.3 | 7.1 ± 1.0 |  |
| Lanthanum (La) | 4.2×10^-3^ ± 3×10^-4^ | 0.8 ± 1.4 | 1.4 ± 1.2 | 4.9 ± 1.4 | 5.6 ± 1.0 | 6.4 ± 0.8 | 4.7 ± 1.1 | 6.9 ± 1.8 | 7.2 ± 1.7 |  |
| Lead (Pb) | 0.31 ± 0.03 | 0.34 ± 0.05 | 1.1 ± 1.2 | 1.4 ± 1.8 | 4 ± 4 | 4 ± 4 | 3 ± 3 | 5 ± 3 | 7 ± 3 |  |
| Lutetium (Lu) | 1.15×10^-2^ ± 3×10^-4^ | 0.4 ± 0.8 | 1.4 ± 0.8 | 4.5 ± 1.1 | 5.8 ± 1.6 | 6.0 ± 0.6 | 4.6 ± 1.5 | 6.7 ± 1.8 | 6.8 ± 1.1 |  |
| Molybdenum (Mo) | 0.106 ± 0.005 | 0.7 ± 1.1 | 1.0 ± 1.4 | 3.6 ± 1.9 | 6 ± 2 | 7.0 ± 0.8 | 14.8 ± 1.9 | 36 ± 4 | 45.8 ± 1.1 |  |
| Neodymium (Nd) | 9.0×10^-3^ ± 4×10^-4^ | 1.3 ± 1.3 | 1.2 ± 1.0 | 3.4 ± 1.3 | 5.3 ± 1.5 | 5.3 ± 0.7 | 4.9 ± 0.9 | 7.2 ± 1.1 | 7.5 ± 0.6 |  |
| Nickel (Ni) | 3.88×10^-2^ ± 1.7×10^-3^ | 1.7 ± 1.8 | 2.3 ± 1.6 | 5.4 ± 1.8 | 9.9 ± 1.8 | 9.2 ± 1.5 | 7.0 ± 0.6 | 10.1 ± 1.4 | 9.9 ± 1.0 |  |
| Niobium (Nb) | 4.42×10^-2^ ± 1.5×10^-3^ | 1.0 ± 1.7 | 0.5 ± 0.8 | 3.7 ± 1.4 | 5.9 ± 1.7 | 4.8 ± 0.9 | 3.4 ± 0.5 | 20.4 ± 1.5 | 91.1 ± 1.4 |  |
| Rhenium (Re) | 3.38×10^-4^ ± 1.2×10^-5^ | 0.4 ± 0.7 | 2.5 ± 1.4 | 3.7 ± 1.2 | 6.6 ± 1.8 | 5.3 ± 0.5 | 5.6 ± 0.9 | 9.0 ± 1.3 | 8.5 ± 0.5 |  |
| Rubidium (Rb) | 2.01×10^-3^ ± 5×10^-5^ | 0.6 ± 1.1 | 1.5 ± 1.2 | 4 ± 2 | 7.2 ± 1.5 | 6.8 ± 1.3 | 4.8 ± 1.1 | 7.7 ± 1.2 | 9.4 ± 1.2 |  |
| Scandium (Sc) | 2.18×10^-2^ ± 8×10^-4^ | 0.6 ± 1.0 | 0.9 ± 1.0 | 4.0 ± 1.3 | 8 ± 2 | 6.1 ± 0.8 | 4.3 ± 0.6 | 7.6 ± 1.2 | 8.7 ± 0.6 |  |
| Silver (Ag) | 0.14 ± 0.03 | 5.6 ± 1.4 | 1.4 ± 1.6 | 3.9 ± 1.5 | 8 ± 2 | 6.9 ± 1.1 | 6.1 ± 0.2 | 9.2 ± 1.7 | 9.5 ± 1.3 |  |
| Strontium (Sr) | 1.54×10^-2^ ± 1.0×10^-3^ | 0.2 ± 0.3 | 2.7 ± 1.1 | 4.4 ± 1.2 | 8 ± 2 | 6.9 ± 0.8 | 4.4 ± 0.9 | 8.7 ± 1.4 | 9.6 ± 0.6 |  |
| Thallium (Tl) | 4.20×10^-2^ ± 1.3×10^-3^ | 2.5 ± 1.7 | 107 ± 13 | 148 ± 13 | 230 ± 60 | 219 ± 19 | 94 ± 4 | 287 ± 5 | 552 ± 12 |  |
| Thorium (Th) | 3.14×10^-2^ ± 1.3×10^-3^ | 0.4 ± 0.7 | 0.4 ± 0.7 | 3.3 ± 1.8 | 5 ± 2 | 4.3 ± 1.5 | 2.9 ± 1.0 | 4.8 ± 0.8 | 5.6 ± 0.7 |  |
| Tin (Sn) | 9.9×10^-2^ ± 3×10^-3^ | 0.4 ± 0.6 | 0.7 ± 1.0 | 4.4 ± 1.9 | 7.1 ± 1.9 | 7.0 ± 1.7 | 4.6 ± 0.6 | 11.1 ± 1.3 | 14 ± 2 |  |
| Tin (^117m^Sn) |  | 4 ± 3 | 14 ± 15 | 23 ± 2 | 27 ± 15 | 10 ± 4 | 30 ± 20 | 21 ± 4 | 18 ± 2 | 28 ± 8 |
| Uranium (U) | 1.260×10^-3^ ± 9×10^-6^ | 0.4 ± 0.8 | 0.9 ± 1.1 | 3.8 ± 1.8 | 6.1 ± 1.6 | 4.8 ± 0.7 | 2.9 ± 1.1 | 6.2 ± 1.0 | 7.7 ± 0.8 |  |
| Ytterbium (Yb) | 0.149 ± 0.006 | 0.9 ± 1.3 | 1.5 ± 1.0 | 4.1 ± 1.4 | 7.4 ± 1.9 | 6.9 ± 0.7 | 4.5 ± 0.7 | 6.1 ± 1.2 | 7.5 ± 1.1 |  |
| Yttrium (Y) | 1.37×10^-3^ ± 3×10^-5^ | 0.4 ± 0.8 | 1.3 ± 1.7 | 4.0 ± 1.6 | 6.2 ± 1.4 | 6.9 ± 0.9 | 6.1 ± 0.8 | 9.1 ± 0.8 | 9.1 ± 1.8 |  |
| Zinc (Zn) | 5.0×10^-2^ ± 3×10^-3^ | 1.1 ± 1.4 | 2.0 ± 1.1 | 3.5 ± 1.9 | 9 ± 2 | 7.7 ± 0.9 | 5.3 ± 1.1 | 8.7 ± 1.3 | 10.0 ± 0.5 |  |
| Zirconium (Zr) | 3.77×10^-2^ ± 1.1×10^-3^ | 0.5 ± 0.7 | 0.9 ± 1.4 | 3.4 ± 1.2 | 6 ± 2 | 5.3 ± 1.9 | 4.9 ± 1.0 | 6.5 ± 1.1 | 8 ± 2 |  |

^[a]^Unless noted otherwise, all K_D_ values were acquired using stable elements from a multi-element standard.

**Table S6**. Distribution coefficient (K_D_) values for DOE-71 resin.

| Element^[a]^ | Distribution coefficients (K_D_) in hydrochloric acid (K_D_ ± SD, n = 3 unless otherwise noted) | | | | | | | | | |
| --- | --- | --- | --- | --- | --- | --- | --- | --- | --- | --- |
|  | 0.01 M | 0.1 M | 0.5 M | 1 M | 2 M | 3 M | 6 M | 8 M | 10 M | 12 M |
| Aluminium (Al) | 6 ± 3 | 0.7 ± 0.6 | 4.4 ± 0.5 | 1.0 ± 0.5 | 3 ± 2 | 3.9 ± 0.7 | 2.5 ± 0.8 | 1.7 ± 0.4 | 0.4 ± 0.3 |  |
| Antimony (Sb) | 0.5 ± 0.8 | 2.59×10^-3^ ± 2×10^-5^ | 2.76×10^-3^ ± 2×10^-5^ | 2.81×10^-3^ ± 1.5×10^-4^ | 0.6 ± 1.1 | 0.5 ± 0.3 | 17.4 ± 1.0 | 640 ± 30 | 14600 ± 200 |  |
| Antimony (Sb, single-element standard) |  |  |  |  |  |  | 179 ± 15 | 1600 ± 500 | 5000 ± 5000 | 8570 ± 170 |
| Antimony (^120m^Sb) |  | 20 ± 5 | 48 ± 3 | 57 ± 7 | 60 ± 10 | 53 ± 2 | 90 ± 30 | 500 ± 300 (n = 6) | 180 ± 30 | 1700 ± 1100 |
| Barium (Ba) | 0.4 ± 0.7 | 3.20×10^-3^ ± 4×10^-5^ | 4.4×10^-3^ ± 1.4×10^-3^ | 3.05×10^-3^ ± 1.7×10^-4^ | 0.5 ± 0.9 | 0.2 ± 0.2 | 0.3 ± 0.3 | 3.70×10^-3^ ± 1.4×10^-4^ | 0.09 ± 0.12 |  |
| Bismuth (Bi) | 1.95 ± 0.07 | 0.38 ± 0.03 | 0.47 ± 0.04 | 0.46 ± 0.03 | 0.46 ± 0.02 | 0.34 ± 0.12 | 0.307 ± 0.003 | 0.230 ± 0.005 | 0.211 ± 0.008 |  |
| Cerium (Ce) | 0.9 ± 1.2 | 2.9 ± 0.4 | 3.42×10^-2^ ± 7×10^-4^ | 6.7×10^-3^ ± 3×10^-4^ | 0.6 ± 1.0 | 0.7 ± 0.3 | 0.8 ± 0.3 | 0.08 ± 0.12 | 0.2 ± 0.4 |  |
| Cesium (Cs) | 9×10^-2^ ± 4×10^-2^ | 1.9×10^-3^ ± 2×10^-4^ | 4×10^-2^ ± 3×10^-2^ | 2.24×10^-3^ ± 1.2×10^-4^ | 0.4 ± 0.7 | 0.6 ± 0.4 | 0.06 ± 0.11 | 2.57×10^-3^ ± 6×10^-5^ | 0.1 ± 0.2 |  |
| Cobalt (Co) | 0.8 ± 1.3 | 5.04×10^-3^ ± 8×10^-5^ | 0.15 ± 0.15 | 5.8×10^-3^ ± 3×10^-4^ | 0.5 ± 0.9 | 0.4 ± 0.4 | 5.70×10^-3^ ± 3×10^-5^ | 5.49×10^-3^ ± 1.2×10^-4^ | 6.3×10^-3^ ± 4×10^-4^ |  |
| Copper (Cu) | 0.8 ± 1.4 | 6×10^-2^ ± 3×10^-2^ | 0.7 ± 0.5 | 4.2×10^-2^ ± 2×10^-3^ | 1.0 ± 1.6 | 0.5 ± 0.6 | 0.16 ± 0.14 | 0.22637 ± 0.00010 | 0.3 ± 0.3 |  |
| Erbium (Er) | 0.5 ± 0.8 | 2.389×10^-4^ ± 1.9×10^-6^ | 5×10^-3^ ± 8×10^-3^ | 2.22×10^-4^ ± 1.2×10^-5^ | 1.1 ± 1.7 | 0.4 ± 0.5 | 0.2 ± 0.3 | 2.01×10^-4^ ± 3×10^-6^ | 2.28×10^-4^ ± 7×10^-6^ |  |
| Europium (Eu) | 0.3 ± 0.5 | 4.72×10^-4^ ± 8×10^-6^ | 0.10 ± 0.15 | 5.7×10^-4^ ± 2×10^-5^ | 0.7 ± 1.3 | 0.2 ± 0.2 | 0.3 ± 0.2 | 3.88×10^-4^ ± 9×10^-6^ | 7.3×10^-4^ ± 3×10^-5^ |  |
| Gallium (Ga) | 0.7 ± 1.1 | 1.064×10^-3^ ± 1.0×10^-5^ | 1.56×10^-3^ ± 5×10^-5^ | 2.08×10^-3^ ± 9×10^-5^ | 0.5 ± 0.9 | 0.2 ± 0.3 | 91 ± 3 | 453 ± 18 | 793 ± 17 |  |
| Hafnium (Hf) | 0.4 ± 0.6 | 0.11 ± 0.16 | 1.53×10^-2^ ± 3×10^-4^ | 1.56×10^-2^ ± 7×10^-4^ | 0.6 ± 1.0 | 0.4 ± 0.4 | 1.461×10^-2^ ± 1.1×10^-4^ | 1.36×10^-2^ ± 2×10^-4^ | 1.34×10^-2^ ± 4×10^-4^ |  |
| Lanthanum (La) | 0.6 ± 1.0 | 1.123×10^-3^ ± 6×10^-6^ | 1.276×10^-3^ ± 1.5×10^-5^ | 1.38×10^-3^ ± 5×10^-5^ | 0.8 ± 1.3 | 0.68 ± 0.16 | 1.71×10^-3^ ± 2×10^-5^ | 1.40×10^-3^ ± 3×10^-5^ | 1.69×10^-3^ ± 8×10^-5^ |  |
| Lead (Pb) | 0.122 ± 0.010 | 0.103 ± 0.003 | 0.118 ± 0.007 | 0.119 ± 0.008 | 0.124 ± 0.007 | 0.11 ± 0.04 | 0.128 ± 0.003 | 0.1068 ± 0.0014 | 0.111 ± 0.004 |  |
| Lutetium (Lu) | 0.5 ± 0.9 | 2.24×10^-4^ ± 3×10^-6^ | 0.2 ± 0.3 | 1.98×10^-4^ ± 8×10^-6^ | 0.8 ± 1.4 | 1.0 ± 0.7 | 0.2 ± 0.3 | 2.052×10^-4^ ± 1.4×10^-6^ | 0.04 ± 0.07 |  |
| Molybdenum (Mo) | 0.12 ± 0.07 | 0.767 ± 0.0005 | 0.3 ± 0.3 | 8.3×10^-2^ ± 5×10^-3^ | 0.7 ± 1.0 | 0.8 ± 0.7 | 2.0 ± 0.7 | 7.1 ± 0.5 | 24.6 ± 1.3 |  |
| Neodymium (Nd) | 0.2 ± 0.3 | 0.60 ± 0.02 | 8.58×10^-3^ ± 7×10^-5^ | 2.63×10^-3^ ± 1.2×10^-4^ | 0.8 ± 1.3 | 0.8 ± 0.3 | 0.4 ± 0.4 | 2.44×10^-3^ ± 8×10^-5^ | 0.09 ± 0.15 |  |
| Nickel (Ni) | 1.0 ± 1.6 | 0.2 ± 0.2 | 0.7 ± 0.5 | 0.3 ± 0.5 | 1.3 ± 1.6 | 1.06 ± 0.19 | 1.0 ± 0.8 | 0.186 ± 0.006 | 0.2 ± 0.2 |  |
| Niobium (Nb) | 0.9 ± 1.4 | 7.50×10^-2^ ± 7×10^-4^ | 0.5 ± 0.5 | 7.0×10^-2^ ± 4×10^-3^ | 1.1 ± 1.7 | 7.99×10^-2^ ± 8×10^-4^ | 0.3 ± 0.3 | 0.8 ± 0.5 | 47.3 ± 1.4 |  |
| Rhenium (Re) | 0.8 ± 1.3 | 6.795×10^-5^ ± 1.8×10^-7^ | 2.14×10^-5^ ± 6×10^-7^ | 3.04×10^-5^ ± 1.5×10^-6^ | 0.5 ± 0.9 | 0.10 ± 0.17 | 7.03×10^-5^ ± 5×10^-7^ | 7.55×10^-5^ ± 1.5×10^-6^ | 0.7 ± 0.9 |  |
| Rubidium (Rb) | 7.7×10^-3^ ± 4×10^-4^ | 1.44×10^-3^ ± 3×10^-5^ | 0.2 ± 0.4 | 1.44×10^-3^ ± 9×10^-5^ | 0.9 ± 1.3 | 0.2 ± 0.4 | 0.4 ± 0.6 | 1.725×10^-3^ ± 1.7×10^-5^ | 0.2 ± 0.4 |  |
| Scandium (Sc) | 0.2 ± 0.4 | 1.808×10^-2^ ± 9×10^-5^ | 0.15 ± 0.19 | 1.94×10^-2^ ± 1.2×10^-3^ | 0.6 ± 0.9 | 0.3 ± 0.5 | 1.791×10^-2^ ± 1.9×10^-4^ | 1.72×10^-2^ ± 3×10^-4^ | 1.77×10^-2^ ± 9×10^-4^ |  |
| Silver (Ag) | 8×10^-2^ ± 4×10^-2^ | 2.3 ± 0.6 | 0.2 ± 0.2 | 1.22×10^-2^ ± 5×10^-4^ | 0.5 ± 0.8 | 0.9 ± 0.4 | 0.2 ± 0.4 | 4.76×10^-3^ ± 9×10^-5^ | 0.07 ± 0.10 |  |
| Strontium (Sr) | 1.9×10^-2^ ± 4×10^-3^ | 1.17×10^-2^ ± 7×10^-4^ | 0.11 ± 0.17 | 1.7×10^-2^ ± 1.0×10^-2^ | 0.9 ± 1.6 | 1.2 ± 0.5 | 1.145×10^-2^ ± 1.9×10^-4^ | 1.20×10^-2^ ± 4×10^-4^ | 1.26×10^-2^ ± 5×10^-4^ |  |
| Thallium (Tl) | 0.4 ± 0.6 | 1.5 ± 0.3 | 18.7 ± 1.2 | 44 ± 7 | 49 ± 4 | 53.8 ± 1.1 | 15.7 ± 0.9 | 27 ± 2 | 258 ± 18 |  |
| Thorium (Th) | 0.2 ± 0.3 | 2.266×10^-2^ ± 1.6×10^-4^ | 6×10^-2^ ± 7×10^-2^ | 2.31×10^-2^ ± 1.3×10^-3^ | 0.3 ± 0.4 | 6×10^-2^ ± 6×10^-2^ | 2.401×10^-2^ ± 1.9×10^-4^ | 1.70×10^-2^ ± 7×10^-4^ | 1.87×10^-2^ ± 1.0×10^-3^ |  |
| Tin (Sn) | 0.4 ± 0.6 | 6.60×10^-2^ ± 6×10^-4^ | 7.42×10^-2^ ± 9×10^-4^ | 6.5×10^-2^ ± 3×10^-3^ | 0.4 ± 0.6 | 0.6 ± 0.3 | 0.5 ± 0.8 | 5.02×10^-2^ ± 7×10^-4^ | 2.5 ± 0.4 |  |
| Tin (^117m^Sn) |  | 1.3 ± 1.7 | 1.0 ± 1.4 | 9 ± 6 | 7 ± 2 | 3.0 ± 0.9 | 9 ± 6 | 2.4 ± 1.8 | 5 ± 7 | 8.4 ± 0.7 |
| Uranium (U) | 0.4 ± 0.7 | 9.4×10^-4^ ± 3×10^-5^ | 0.08 ± 0.14 | 3.47×10^-4^ ± 1.8×10^-5^ | 0.4 ± 0.7 | 0.2 ± 0.3 | 5.58×10^-4^ ± 5×10^-6^ | 5.12×10^-4^ ± 1.7×10^-5^ | 5.1×10^-4^ ± 2×10^-5^ |  |
| Ytterbium (Yb) | 0.17 ± 0.03 | 2.8×10^-2^ ± 3×10^-3^ | 3.54×10^-2^ ± 4×10^-4^ | 5.8×10^-2^ ± 3×10^-3^ | 0.6 ± 1.0 | 0.8 ± 0.8 | 0.3 ± 0.4 | 1.238×10^-2^ ± 1.2×10^-4^ | 8.4×10^-2^ ± 1.1×10^-2^ |  |
| Yttrium (Y) | 0.3 ± 0.5 | 4.12×10^-4^ ± 7×10^-6^ | 2.77×10^-4^ ± 7×10^-6^ | 2.75×10^-4^ ± 1.1×10^-5^ | 0.8 ± 1.3 | 0.4 ± 0.6 | 0.06 ± 0.10 | 5.33×10^-4^ ± 1.2×10^-5^ | 7.19×10^-4^ ± 1.0×10^-5^ |  |
| Zinc (Zn) | 0.7 ± 1.0 | 0.31 ± 0.10 | 0.2 ± 0.3 | 0.2 ± 0.4 | 1.3 ± 1.8 | 1.0 ± 0.7 | 0.21 ± 0.19 | 1.648 ± 0.006 | 0.3 ± 0.3 |  |
| Zirconium (Zr) | 0.4 ± 0.7 | 2.66×10^-2^ ± 4×10^-4^ | 2.93×10^-2^ ± 4×10^-4^ | 3.11×10^-2^ ± 1.7×10^-3^ | 0.6 ± 0.9 | 7×10^-2^ ± 4×10^-2^ | 9×10^-2^ ± 7×10^-2^ | 3.66×10^-2^ ± 3×10^-4^ | 3.91×10^-2^ ± 7×10^-4^ |  |

^[a]^Unless noted otherwise, all K_D_ values were acquired using stable elements from a multi-element standard.

**Table S7**. Distribution coefficient (K_D_) values for TK401-71 resin.

| Element^[a]^ | Distribution coefficients (K_D_) in hydrochloric acid (K_D_ ± SD, n = 3) | | | | | | | | | |
| --- | --- | --- | --- | --- | --- | --- | --- | --- | --- | --- |
|  | 0.01 M | 0.1 M | 0.5 M | 1 M | 2 M | 3 M | 6 M | 8 M | 10 M | 12 M |
| Aluminium (Al) | 13 ± 3 | 1.85 ± 0.17 | 4.1 ± 1.7 | 3 ± 2 | 0.962 ± 0.016 | 1.7 ± 1.3 | 1.81 ± 0.02 | 2.2 ± 0.3 | 1.1 ± 0.9 |  |
| Antimony (Sb) | 0.3 ± 0.4 | 0.5 ± 0.4 | 0.9 ± 0.8 | 1 ± 1 | 0.3 ± 0.2 | 1 ± 1 | 15 ± 2 | 132 ± 4 | 6200 ± 700 |  |
| Antimony (Sb, single-element standard) |  |  |  |  |  |  | 23.7 ± 1.4 | 105 ± 5 | 650 ± 20 | 700 ± 100 |
| Antimony (^120m^Sb) |  | 16.9 ± 1.2 | 24.5 ± 0.8 | 27.4 ± 0.4 | 29.1 ± 1.4 | 34 ± 2 | 55.9 ± 1.7 | 305 ± 12 | 870 ± 30 | 2200 ± 130 |
| Barium (Ba) | 0.3 ± 0.2 | 0.4 ± 0.4 | 1.0 ± 0.9 | 1.9 ± 0.9 | 0.3 ± 0.3 | 1.2 ± 1.0 | 1.8 ± 1.0 | 5.05×10^-3^ ± 1.2×10^-4^ | 3.7 ± 1.0 |  |
| Bismuth (Bi) | 27.7 ± 1.9 | 0.71 ± 0.08 | 0.84 ± 0.05 | 1.1 ± 0.3 | 0.86 ± 0.06 | 1.0 ± 0.6 | 2.3 ± 1.1 | 0.49 ± 0.12 | 3.2 ± 1.1 |  |
| Cerium (Ce) | 0.4 ± 0.6 | 4 ± 3 | 0.8 ± 0.9 | 1.1 ± 0.9 | 4×10^-2^ ± 3×10^-2^ | 1.3 ± 1.5 | 2.1 ± 1.3 | 3.26×10^-2^ ± 5×10^-4^ | 2.1 ± 0.9 |  |
| Cesium (Cs) | 0.6 ± 0.5 | 0.4 ± 0.5 | 1.2 ± 1.1 | 1.4 ± 1.3 | 0.2 ± 0.3 | 0.8 ± 1.3 | 1.7 ± 1.1 | 3.23×10^-3^ ± 6×10^-5^ | 2.9 ± 0.9 |  |
| Cobalt (Co) | 8.8×10^-2^ ± 4×10^-3^ | 0.7 ± 0.6 | 1.5 ± 0.4 | 2.1 ± 0.8 | 0.4 ± 0.7 | 2.3 ± 1.1 | 1.4 ± 1.3 | 2.71×10^-2^ ± 5×10^-4^ | 3.4 ± 1.1 |  |
| Copper (Cu) | 0.279 ± 0.017 | 1.1 ± 0.8 | 1.4 ± 0.8 | 1.9 ± 1.1 | 0.4 ± 0.3 | 1.4 ± 1.3 | 1.2 ± 0.8 | 0.23 ± 0.02 | 3.9 ± 1.1 |  |
| Erbium (Er) | 3.6×10^-2^ ± 2×10^-3^ | 0.8 ± 0.7 | 1.2 ± 1.0 | 1.6 ± 1.1 | 0.4 ± 0.4 | 0.7 ± 0.9 | 2.0 ± 0.8 | 7×10^-3^ ± 1.1×10^-2^ | 2.9 ± 0.4 |  |
| Europium (Eu) | 4.0×10^-2^ ± 3×10^-3^ | 0.8 ± 0.5 | 1.3 ± 1.1 | 2.0 ± 1.2 | 0.3 ± 0.3 | 1.3 ± 1.8 | 1.2 ± 1.1 | 1.13×10^-3^ ± 4×10^-5^ | 2.5 ± 1.4 |  |
| Gallium (Ga) | 0.4 ± 0.4 | 0.5 ± 0.4 | 0.9 ± 0.8 | 1.2 ± 1.0 | 2.03×10^-3^ ± 6×10^-5^ | 1.6 ± 1.1 | 202 ± 12 | 495 ± 19 | 766 ± 17 |  |
| Hafnium (Hf) | 5.3×10^-2^ ± 3×10^-3^ | 0.3 ± 0.6 | 0.5 ± 0.6 | 1.8 ± 1.4 | 0.7 ± 0.6 | 2.0 ± 1.4 | 1.5 ± 1.1 | 1.64×10^-2^ ± 5×10^-4^ | 3.4 ± 0.9 |  |
| Lanthanum (La) | 6×10^-2^ ± 3×10^-2^ | 0.7 ± 0.6 | 1.0 ± 1.1 | 1.6 ± 1.1 | 0.6 ± 0.6 | 1.3 ± 1.4 | 1.4 ± 1.0 | 2.93×10^-3^ ± 1.1×10^-4^ | 2.6 ± 0.8 |  |
| Lead (Pb) | 0.379 ± 0.006 | 0.8 ± 0.9 | 1.2 ± 0.9 | 1.2 ± 1.6 | 0.7 ± 0.6 | 1.3 ± 0.9 | 2 ± 2 | 0.293 ± 0.016 | 3.6 ± 0.4 |  |
| Lutetium (Lu) | 5.9×10^-2^ ± 1.9×10^-2^ | 0.4 ± 0.4 | 1.0 ± 1.0 | 1.5 ± 1.5 | 0.6 ± 0.7 | 2.1 ± 1.6 | 1.4 ± 0.7 | 0.2 ± 0.4 | 3.8 ± 1.0 |  |
| Molybdenum (Mo) | 0.142 ± 0.008 | 0.8 ± 0.7 | 0.9 ± 0.8 | 2.1 ± 0.9 | 7×10^-2^ ± 6×10^-2^ | 2.2 ± 1.3 | 6.6 ± 1.3 | 5.4 ± 0.9 | 13.6 ± 0.9 |  |
| Neodymium (Nd) | 0.6 ± 0.5 | 1.2 ± 1.1 | 1.3 ± 0.9 | 1.6 ± 1.5 | 6×10^-2^ ± 6×10^-2^ | 1.4 ± 1.4 | 1.7 ± 0.8 | 0.136 ± 0.005 | 2.0 ± 0.5 |  |
| Nickel (Ni) | 0.6 ± 0.3 | 0.7 ± 0.5 | 1.9 ± 1.1 | 1.4 ± 0.2 | 0.37 ± 0.09 | 1.4 ± 1.3 | 1.3 ± 1.0 | 0.33 ± 0.09 | 2.4 ± 1.4 |  |
| Niobium (Nb) | 0.19 ± 0.13 | 0.4 ± 0.5 | 1.6 ± 1.2 | 1.4 ± 1.2 | 6.83×10^-2^ ± 3×10^-4^ | 1.3 ± 0.7 | 1.0 ± 1.0 | 2.5 ± 0.5 | 14.4 ± 0.9 |  |
| Rhenium (Re) | 0.5 ± 0.4 | 1.6 ± 0.6 | 1.7 ± 1.0 | 2.8 ± 1.0 | 1.1 ± 0.9 | 2.6 ± 1.2 | 1.9 ± 1.0 | 0.9 ± 0.4 | 4.5 ± 1.2 |  |
| Rubidium (Rb) | 0.2 ± 0.2 | 0.4 ± 0.3 | 1.9 ± 1.4 | 2.1 ± 1.4 | 0.2 ± 0.2 | 2.0 ± 0.7 | 1.5 ± 0.8 | 0.3 ± 0.5 | 3.6 ± 0.7 |  |
| Scandium (Sc) | 6.2×10^-2^ ± 5×10^-3^ | 0.4 ± 0.3 | 2.0 ± 0.9 | 1.5 ± 1.0 | 0.2 ± 0.3 | 1.3 ± 1.0 | 1.5 ± 1.2 | 2.50×10^-2^ ± 1.5×10^-3^ | 3.0 ± 1.3 |  |
| Silver (Ag) | 0.5 ± 0.3 | 0.8 ± 0.7 | 2.2 ± 0.7 | 2.7 ± 0.9 | 0.8 ± 0.4 | 1.3 ± 1.3 | 0.8 ± 0.7 | 7.08×10^-3^ ± 1.3×10^-4^ | 3.8 ± 0.2 |  |
| Strontium (Sr) | 0.112 ± 0.006 | 0.6 ± 0.8 | 1.5 ± 1.3 | 0.8 ± 0.8 | 0.4 ± 0.2 | 1.1 ± 1.1 | 1.2 ± 0.7 | 4.7×10^-2^ ± 2×10^-3^ | 2.6 ± 1.4 |  |
| Thallium (Tl) | 7.2×10^-2^ ± 3×10^-3^ | 2.7 ± 0.7 | 26.2 ± 1.4 | 58 ± 4 | 54 ± 4 | 58 ± 9 | 45 ± 2 | 34.4 ± 0.3 | 52 ± 6 |  |
| Thorium (Th) | 7.1×10^-2^ ± 3×10^-3^ | 0.3 ± 0.3 | 1.2 ± 0.9 | 1.6 ± 1.1 | 0.7 ± 0.3 | 2.3 ± 1.0 | 1.7 ± 1.0 | 3.21×10^-2^ ± 1.1×10^-3^ | 3.4 ± 1.3 |  |
| Tin (Sn) | 9.3×10^-2^ ± 6×10^-3^ | 0.7 ± 0.8 | 0.8 ± 0.6 | 1.9 ± 0.9 | 0.7 ± 0.6 | 1.6 ± 1.1 | 1.7 ± 0.9 | 0.3 ± 0.4 | 5.1 ± 1.5 |  |
| Tin (^117m^Sn) |  | 2.4 ± 1.7 | 2.8 ± 0.4 | 2.8 ± 1.6 | 1.3 ± 1.1 | 2.0 ± 0.4 | 2 ± 3 | 10 ± 8 | 2.7 ± 1.9 | 6.3 ± 1.7 |
| Uranium (U) | 3.1×10^-2^ ± 1.1×10^-2^ | 0.8 ± 0.3 | 1.0 ± 0.8 | 1.3 ± 1.2 | 0.5 ± 0.6 | 1.6 ± 1.6 | 1.0 ± 0.7 | 0.07 ± 0.11 | 3.8 ± 1.1 |  |
| Ytterbium (Yb) | 1.3 ± 0.9 | 0.9 ± 1.0 | 0.9 ± 0.8 | 1.1 ± 1.1 | 0.2 ± 0.2 | 1.0 ± 1.6 | 1.2 ± 1.2 | 0.07 ± 0.06 | 3.16 ± 0.08 |  |
| Yttrium (Y) | 0.2 ± 0.3 | 1.1 ± 0.8 | 2.5 ± 0.8 | 0.7 ± 0.5 | 1×10^-2^ ± 2×10^-2^ | 0.9 ± 0.9 | 1.1 ± 0.8 | 1.25×10^-3^ ± 5×10^-5^ | 2.7 ± 0.7 |  |
| Zinc (Zn) | 0.23 ± 0.10 | 0.6 ± 0.4 | 0.7 ± 0.7 | 1.2 ± 1.2 | 6.94×10^-2^ ± 1.1×10^-3^ | 1.6 ± 1.4 | 0.9 ± 1.0 | 7.0×10^-2^ ± 2×10^-3^ | 3.4 ± 1.0 |  |
| Zirconium (Zr) | 6.7×10^-2^ ± 4×10^-3^ | 1.0 ± 0.9 | 1.8 ± 1.1 | 1.0 ± 0.9 | 4.10×10^-2^ ± 2×10^-4^ | 1.6 ± 0.6 | 0.6 ± 0.8 | 4.5×10^-2^ ± 1.0×10^-2^ | 2.7 ± 0.6 |  |

^[a]^Unless noted otherwise, all K_D_ values were acquired using stable elements from a multi-element standard.

**Table S8**. Distribution coefficient (K_D_) values for CG71 resin support.

| Element^[a]^ | Distribution coefficients (K_D_) in hydrochloric acid (K_D_ ± SD, n = 3) | | | | | | | | | |
| --- | --- | --- | --- | --- | --- | --- | --- | --- | --- | --- |
|  | 0.01 M | 0.1 M | 0.5 M | 1 M | 2 M | 3 M | 6 M | 8 M | 10 M | 12 M |
| Aluminium (Al) | 0.4 ± 0.5 | 1.14 ± 0.06 | 0.11 ± 0.05 | 0.70 ± 0.09 | 1.00 ± 0.03 | 1.06 ± 0.03 | 0.9 ± 0.4 | 19.0 ± 0.9 | 1.09 ± 0.07 |  |
| Antimony (Sb) | 6.6×10^-2^ ± 3×10^-3^ | 5.03×10^-3^ ± 1.1×10^-4^ | 0.04 ± 0.03 | 4.75×10^-3^ ± 4×10^-5^ | 7.6×10^-3^ ± 3×10^-4^ | 0.8 ± 0.3 | 1580 ± 60 | 8000 ± 6000 | 24000 ± 4000 |  |
| Antimony (Sb, single-element standard) |  |  |  |  |  |  | 4100 ± 600 | 2300 ± 300 | 4000 ± 800 | 107 ± 4 |
| Barium (Ba) | 0.84 ± 0.06 | 0.2 ± 0.4 | 0.7 ± 0.6 | 2.67×10^-3^ ± 2×10^-5^ | 0.3 ± 0.6 | 4.73×10^-3^ ± 4×10^-5^ | 3.92×10^-3^ ± 1.4×10^-4^ | 4.76×10^-3^ ± 1.6×10^-4^ | 0.8 ± 0.7 |  |
| Bismuth (Bi) | 2.56 ± 0.08 | 2.9 ± 1.5 | 4 ± 2 | 2.1 ± 1.2 | 0.63 ± 0.07 | 0.9 ± 0.3 | 0.435 ± 0.011 | 0.34 ± 0.07 | 1.2 ± 0.5 |  |
| Cerium (Ce) | 0.3 ± 0.3 | 5.82×10^-3^ ± 1.7×10^-4^ | 0.15 ± 0.17 | 6.56×10^-3^ ± 4×10^-5^ | 6.2×10^-3^ ± 2×10^-4^ | 2.23×10^-2^ ± 3×10^-4^ | 6.2×10^-3^ ± 2×10^-4^ | 4.78×10^-3^ ± 1.1×10^-4^ | 0.5 ± 0.6 |  |
| Cesium (Cs) | 0.65 ± 0.19 | 0.4 ± 0.3 | 0.2 ± 0.3 | 0.07 ± 0.11 | 0.3 ± 0.4 | 1.05×10^-2^ ± 3×10^-4^ | 2.93×10^-3^ ± 1.0×10^-4^ | 3.70×10^-3^ ± 1.0×10^-4^ | 1.1 ± 0.5 |  |
| Cobalt (Co) | 0.6 ± 0.3 | 0.11 ± 0.19 | 0.2 ± 0.3 | 3.14×10^-3^ ± 9×10^-5^ | 0.6 ± 0.6 | 3.94×10^-3^ ± 5×10^-5^ | 2.91×10^-3^ ± 1.0×10^-4^ | 0.3 ± 0.3 | 1.0 ± 1.0 |  |
| Copper (Cu) | 0.7 ± 0.6 | 5.73×10^-2^ ± 1.0×10^-3^ | 0.17 ± 0.07 | 3.34×10^-2^ ± 1.0×10^-3^ | 0.4 ± 0.3 | 6.1×10^-2^ ± 2×10^-3^ | 0.203 ± 0.005 | 0.6 ± 0.4 | 0.8 ± 0.2 |  |
| Erbium (Er) | 4×10^-2^ ± 4×10^-2^ | 3.79×10^-4^ ± 9×10^-6^ | 0.1 ± 0.2 | 4.76×10^-4^ ± 6×10^-6^ | 1.14×10^-3^ ± 3×10^-5^ | 5.91×10^-4^ ± 1.5×10^-5^ | 5.8×10^-4^ ± 2×10^-5^ | 4.06×10^-4^ ± 1.4×10^-5^ | 0.9 ± 0.9 |  |
| Europium (Eu) | 2.44×10^-3^ ± 9×10^-5^ | 5.48×10^-4^ ± 9×10^-6^ | 7.37×10^-4^ ± 9×10^-6^ | 6.79×10^-4^ ± 4×10^-6^ | 1.32×10^-3^ ± 6×10^-5^ | 6.81×10^-4^ ± 3×10^-6^ | 9.6×10^-4^ ± 3×10^-5^ | 7.8×10^-4^ ± 3×10^-5^ | 0.5 ± 0.5 |  |
| Gallium (Ga) | 1.63×10^-3^ ± 2×10^-5^ | 1.59×10^-3^ ± 4×10^-5^ | 6×10^-2^ ± 7×10^-2^ | 1.95×10^-3^ ± 4×10^-5^ | 2.7 ± 0.5 | 20.1 ± 0.8 | 580 ± 30 | 640 ± 40 | 700 ± 30 |  |
| Hafnium (Hf) | 6×10^-2^ ± 4×10^-2^ | 1.80×10^-2^ ± 4×10^-4^ | 0.1 ± 0.2 | 1.74×10^-2^ ± 2×10^-4^ | 3×10^-2^ ± 3×10^-2^ | 1.597×10^-2^ ± 1.3×10^-4^ | 1.52×10^-2^ ± 5×10^-4^ | 1.55×10^-2^ ± 5×10^-4^ | 0.5 ± 0.5 |  |
| Lanthanum (La) | 0.63 ± 0.08 | 0.2 ± 0.3 | 0.3 ± 0.5 | 2.310×10^-3^ ± 7×10^-6^ | 2×10^-2^ ± 2×10^-2^ | 2.29×10^-3^ ± 3×10^-5^ | 4×10^-2^ ± 6×10^-2^ | 2.27×10^-3^ ± 6×10^-5^ | 0.4 ± 0.4 |  |
| Lead (Pb) | 0.41 ± 0.02 | 0.40 ± 0.02 | 0.391 ± 0.018 | 0.36 ± 0.03 | 0.314 ± 0.012 | 0.33 ± 0.06 | 0.278 ± 0.019 | 0.25 ± 0.08 | 0.5 ± 0.2 |  |
| Lutetium (Lu) | 0.10 ± 0.16 | 4.62×10^-4^ ± 1.1×10^-5^ | 0.1 ± 0.3 | 2.91×10^-4^ ± 3×10^-6^ | 0.2 ± 0.3 | 5.15 ×10^-4^ ± 1.3×10^-5^ | 1.37×10^-3^ ± 4×10^-5^ | 1.01×10^-3^ ± 3×10^-5^ | 0.9 ± 0.7 |  |
| Molybdenum (Mo) | 7.07×10^-2^ ± 1.7×10^-3^ | 7.59×10^-2^ ± 1.9×10^-3^ | 8.13×10^-2^ ± 1.8×10^-3^ | 7.16×10^-2^ ± 1.1×10^-3^ | 6.7 ± 0.3 | 34.4 ± 1.3 | 140 ± 7 | 144 ± 8 | 99 ± 2 |  |
| Neodymium (Nd) | 2.28×10^-2^ ± 3×10^-4^ | 3.02×10^-3^ ± 9×10^-5^ | 1.16×10^-2^ ± 5×10^-4^ | 3.24×10^-3^ ± 7×10^-5^ | 3.01×10^-3^ ± 1.3×10^-4^ | 8.0×10^-3^ ± 2×10^-4^ | 3.66×10^-3^ ± 9×10^-5^ | 2.15×10^-3^ ± 3×10^-5^ | 0.5 ± 0.6 |  |
| Nickel (Ni) | 0.17 ± 0.14 | 0.7 ± 0.5 | 0.20 ± 0.17 | 5.23×10^-3^ ± 8×10^-5^ | 0.4 ± 0.5 | 0.614 ± 0.009 | 7.1×10^-3^ ± 2×10^-4^ | 8.89×10^-3^ ± 1.6×10^-4^ | 1.2 ± 0.7 |  |
| Niobium (Nb) | 7.7×10^-2^ ± 3×10^-3^ | 9.5×10^-2^ ± 3×10^-3^ | 7.18×10^-2^ ± 9×10^-4^ | 6.30×10^-2^ ± 3×10^-4^ | 0.11 ± 0.07 | 7.04×10^-2^ ± 1.2×10^-3^ | 10.8 ± 1.0 | 150 ± 4 | 263 ± 11 |  |
| Rhenium (Re) | 54 ± 2 | 65.5 ± 0.3 | 88 ± 2 | 95.9 ± 1.6 | 99 ± 2 | 82 ± 3 | 33.7 ± 1.4 | 21.9 ± 0.7 | 10.8 ± 1.1 |  |
| Rubidium (Rb) | 2.80×10^-3^ ± 6×10^-5^ | 2.11×10^-3^ ± 3×10^-5^ | 1.79×10^-2^ ± 7×10^-4^ | 0.11 ± 0.19 | 0.13 ± 0.17 | 2.16×10^-3^ ± 3×10^-5^ | 2.82×10^-3^ ± 9×10^-5^ | 0.08 ± 0.14 | 1.2 ± 0.4 |  |
| Scandium (Sc) | 0.2 ± 0.3 | 0.4 ± 0.4 | 0.2 ± 0.2 | 2.2×10^-2^ ± 9×10^-3^ | 0.4 ± 0.5 | 1.577×10^-2^ ± 1.1×10^-4^ | 1.52×10^-2^ ± 3×10^-4^ | 1.73×10^-2^ ± 7×10^-4^ | 0.5 ± 0.5 |  |
| Silver (Ag) | 8 ± 6 | 5.0 ± 1.1 | 7.6 ± 1.1 | 5.2 ± 0.4 | 1.9 ± 0.5 | 1.023×10^-2^ ± 8×10^-5^ | 8.3×10^-3^ ± 5×10^-4^ | 8.5×10^-3^ ± 3×10^-4^ | 0.9 ± 0.5 |  |
| Strontium (Sr) | 1.26×10^-2^ ± 4×10^-4^ | 2.44×10^-2^ ± 6×10^-4^ | 2.8 ± 0.3 | 1.052×10^-2^ ± 9×10^-5^ | 0.21 ± 0.18 | 1.46×10^-2^ ± 5×10^-4^ | 1.24×10^-2^ ± 4×10^-4^ | 0.9 ± 0.5 | 3.6 ± 0.5 |  |
| Thallium (Tl) | 2 ± 3 | 2.4 ± 0.4 | 46 ± 3 | 2980 ± 190 | 19000 ± 14000 | 2430 ± 50 | 8400 ± 1300 | 5300 ± 400 | 1680 ± 160 |  |
| Thorium (Th) | 3.14×10^-2^ ± 6×10^-4^ | 3.22×10^-2^ ± 5×10^-4^ | 3.56×10^-2^ ± 8×10^-4^ | 2.98×10^-2^ ± 4×10^-4^ | 2.65×10^-2^ ± 7×10^-4^ | 2.49×10^-2^ ± 4×10^-4^ | 2.50×10^-2^ ± 4×10^-4^ | 2.73×10^-2^ ± 1.0×10^-3^ | 0.7 ± 0.4 |  |
| Tin (Sn) | 0.10 ± 0.02 | 9.75×10^-2^ ± 1.1×10^-3^ | 6.3 ± 0.4 | 24.0 ± 0.7 | 78.6 ± 1.7 | 102 ± 3 | 78 ± 4 | 45.9 ± 1.6 | 25.0 ± 0.7 |  |
| Uranium (U) | 3×10^-2^ ± 6×10^-2^ | 7.6×10^-4^ ± 2×10^-5^ | 2.14×10^-3^ ± 7×10^-5^ | 4.86×10^-4^ ± 4×10^-6^ | 0.2 ± 0.4 | 7.77×10^-4^ ± 1.4×10^-6^ | 0.7 ± 0.4 | 4.0 ± 0.6 | 6.9 ± 0.7 |  |
| Ytterbium (Yb) | 0.146 ± 0.004 | 1.11×10^-2^ ± 2×10^-4^ | 3.75×10^-3^ ± 1.7×10^-4^ | 1.276×10^-2^ ± 1.5×10^-4^ | 0.2 ± 0.2 | 1.074×10^-2^ ± 1.5×10^-4^ | 1.19×10^-2^ ± 5×10^-4^ | 5.5×10^-2^ ± 1.4×10^-2^ | 1.1 ± 0.9 |  |
| Yttrium (Y) | 8.8×10^-4^ ± 4×10^-5^ | 7.60×10^-4^ ± 6×10^-6^ | 7.3×10^-3^ ± 5×10^-4^ | 2.17×10^-4^ ± 6×10^-6^ | 0.16 ± 0.10 | 9.66×10^-4^ ± 6×10^-6^ | 1.21×10^-3^ ± 4×10^-5^ | 6.6×10^-4^ ± 4×10^-5^ | 0.4 ± 0.5 |  |
| Zinc (Zn) | 0.86 ± 0.05 | 0.95 ± 0.04 | 1.41 ± 0.06 | 0.91 ± 0.02 | 0.8 ± 0.4 | 0.97 ± 0.03 | 0.99 ± 0.04 | 1.20 ± 0.03 | 0.94 ± 0.15 |  |
| Zirconium (Zr) | 2.85×10^-2^ ± 1.3×10^-3^ | 3.03×10^-2^ ± 1.7×10^-3^ | 3.12×10^-2^ ± 8×10^-4^ | 2.936×10^-2^ ± 1.0×10^-4^ | 0.29 ± 0.03 | 3.25×10^-2^ ± 6×10^-4^ | 3.8×10^-2^ ± 2×10^-3^ | 4.00×10^-2^ ± 1.6×10^-3^ | 0.3 ± 0.5 |  |

^[a]^Unless noted otherwise, all K_D_ values were acquired using stable elements from a multi-element standard.

**Table S9**. Distribution coefficient (K_D_) values for DBE-300 resin.

| Element^[a]^ | Distribution coefficients (K_D_) in hydrochloric acid (K_D_ ± SD, n = 3) | | | | | | | | | |
| --- | --- | --- | --- | --- | --- | --- | --- | --- | --- | --- |
|  | 0.01 M | 0.1 M^[b]^ | 0.5 M | 1 M | 2 M | 3 M | 6 M | 8 M | 10 M | 12 M |
| Aluminium (Al) | 3 ± 2 | < 2 | 1.7 ± 1.4 | 0.6 ± 0.7 | 1.1 ± 0.9 | 1.08 ± 0.04 | 5 ± 7 | 1.56 ± 0.05 | 1.32 ± 0.17 |  |
| Antimony (Sb) | 0.6 ± 1.1 | < 2 | 0.8 ± 0.2 | 1.3 ± 0.6 | 2.7 ± 0.9 | 0.6 ± 0.4 | 1.1 ± 0.6 | 31 ± 2 | 1730 ± 110 |  |
| Antimony (Sb, single-element standard) |  |  |  |  |  |  | 52 ± 8 | 102 ± 12 | 510 ± 120 | 87 ± 14 |
| Antimony (^120m^Sb) |  | 13.0 ± 1.1 | 25.6 ± 1.4 | 26.0 ± 1.1 | 27 ± 4 | 31.0 ± 1.5 | 28 ± 2 | 61 ± 2 | 260 ± 20 | 760 ± 90 |
| Barium (Ba) | 0.8 ± 1.2 | < 2 | 1.90 ± 0.07 | 1.7 ± 0.9 | 2.9 ± 1.0 | 1.0 ± 0.2 | 4.72×10^-3^ ± 1.9×10^-4^ | 0.1 ± 0.2 | 0.3 ± 0.3 |  |
| Bismuth (Bi) | 1.42 ± 0.04 | < 2 | 0.65 ± 0.04 | 0.68 ± 0.02 | 2 ± 2 | 1.1 ± 0.4 | 0.53 ± 0.15 | 0.7 ± 0.6 | 1.7 ± 0.6 |  |
| Cerium (Ce) | 1.1 ± 1.5 | < 2 | 1.70 ± 0.15 | 2.3 ± 0.6 | 2.9 ± 1.1 | 1.1 ± 0.5 | 6.3×10^-3^ ± 2×10^-4^ | 0.4 ± 0.7 | 0.9 ± 0.5 |  |
| Cesium (Cs) | 0.8 ± 1.3 | < 2 | 1.7 ± 0.4 | 1.5 ± 0.8 | 6 ± 6 | 1.3 ± 0.5 | 0.4 ± 0.4 | 0.6 ± 1.1 | 0.6 ± 0.5 |  |
| Cobalt (Co) | 1.6 ± 1.5 | < 2 | 1.6 ± 0.2 | 1.6 ± 0.4 | 4.3 ± 1.3 | 2.0 ± 0.9 | 6×10^-2^ ± 9×10^-2^ | 0.9 ± 1.1 | 1.4 ± 0.9 |  |
| Copper (Cu) | 1.5 ± 1.2 | < 2 | 2.14 ± 0.19 | 1.3 ± 0.7 | 4 ± 3 | 1.4 ± 0.6 | 3.94×10^-2^ ± 6×10^-4^ | 0.5 ± 0.9 | 0.3 ± 0.4 |  |
| Erbium (Er) | 0.6 ± 1.0 | < 2 | 1.31 ± 0.04 | 1.5 ± 0.5 | 2.9 ± 1.4 | 1.2 ± 0.5 | 3.08×10^-2^ ± 1.2×10^-3^ | 0.6 ± 0.9 | 0.9 ± 0.2 |  |
| Europium (Eu) | 0.5 ± 0.9 | < 2 | 1.3 ± 0.5 | 1.3 ± 0.8 | 3.4 ± 1.0 | 1.1 ± 0.6 | 2×10^-2^ ± 3×10^-2^ | 0.3 ± 0.5 | 0.7 ± 0.4 |  |
| Gallium (Ga) | 1.3 ± 1.4 | < 2 | 0.7 ± 0.4 | 2.5 ± 0.4 | 2.3 ± 0.8 | 0.7 ± 1.0 | 8.9 ± 1.1 | 31 ± 4 | 54 ± 6 |  |
| Hafnium (Hf) | 0.7 ± 1.1 | < 2 | 1.04 ± 0.12 | 1.4 ± 0.7 | 2.9 ± 1.2 | 1.05 ± 0.13 | 4×10^-2^ ± 4×10^-2^ | 0.5 ± 0.8 | 0.9 ± 0.3 |  |
| Lanthanum (La) | 1.5 ± 1.5 | < 2 | 1.89 ± 0.04 | 1.9 ± 0.7 | 3.3 ± 0.9 | 1.3 ± 0.5 | 2×10^-2^ ± 3×10^-2^ | 0.2 ± 0.4 | 1.5 ± 0.4 |  |
| Lead (Pb) | 0.52 ± 0.05 | < 2 | 0.53 ± 0.04 | 0.48 ± 0.03 | 2 ± 2 | 0.49 ± 0.05 | 0.521 ± 0.009 | 0.45 ± 0.08 | 1.0 ± 1.2 |  |
| Lutetium (Lu) | 0.9 ± 1.3 | < 2 | 1.5 ± 0.4 | 1.9 ± 0.7 | 2.7 ± 1.3 | 1.3 ± 0.3 | 0.07 ± 0.12 | 0.2 ± 0.4 | 0.9 ± 0.7 |  |
| Molybdenum (Mo) | 9.7×10^-2^ ± 4×10^-3^ | < 2 | 0.53 ± 0.06 | 0.5 ± 0.6 | 2.4 ± 1.4 | 0.94 ± 0.11 | 0.103 ± 0.003 | 0.3 ± 0.4 | 0.9 ± 0.7 |  |
| Neodymium (Nd) | 0.8 ± 1.2 | < 2 | 1.2344 ± 0.0017 | 1.9 ± 0.7 | 3.0 ± 1.3 | 0.5 ± 0.4 | 3.96×10^-3^ ± 1.5×10^-4^ | 0.3 ± 0.6 | 0.6 ± 0.6 |  |
| Nickel (Ni) | 1 ± 2 | < 2 | 1.7 ± 0.6 | 2.2 ± 0.4 | 3.0 ± 0.2 | 1.3 ± 1.0 | 8.41×10^-3^ ± 1.2×10^-4^ | 0.5 ± 0.8 | 0.11 ± 0.17 |  |
| Niobium (Nb) | 0.4 ± 0.5 | < 2 | 0.17 ± 0.10 | 9.2×10^-2^ ± 1.1×10^-2^ | 1.4 ± 1.6 | 7×10^-2^ ± 4×10^-2^ | 0.102 ± 0.002 | 0.9 ± 0.7 | 0.16 ± 0.08 |  |
| Rhenium (Re) | 1.1 ± 1.3 | < 2 | 1.1 ± 0.2 | 0.8 ± 0.7 | 2.8 ± 0.8 | 1.03 ± 0.15 | 0.2 ± 0.3 | 0.4 ± 0.7 | 1.6 ± 0.3 |  |
| Rubidium (Rb) | 0.4 ± 0.7 | < 2 | 1.1 ± 0.6 | 1.4 ± 1.1 | 3 ± 3 | 1.0 ± 0.9 | 1.92×10^-3^ ± 6×10^-5^ | 0.7 ± 1.1 | 0.3 ± 0.4 |  |
| Scandium (Sc) | 1.5 ± 1.2 | < 2 | 0.8 ± 0.7 | 2.2 ± 0.8 | 4.3 ± 1.0 | 1.2 ± 0.4 | 1.85×10^-2^ ± 7×10^-4^ | 0.7 ± 1.2 | 1.6 ± 0.6 |  |
| Silver (Ag) | 4 ± 5 | < 2 | 3.978×10^-2^ ± 1.2×10^-4^ | 1.5 ± 1.1 | 1.9 ± 1.1 | 0.17 ± 0.14 | 1.37×10^-2^ ± 3×10^-4^ | 0.3 ± 0.6 | 0.3 ± 0.5 |  |
| Strontium (Sr) | 0.5 ± 0.8 | < 2 | 1.4 ± 0.7 | 0.7 ± 0.6 | 2.1 ± 1.2 | 1.1 ± 0.7 | 1.9×10^-2^ ± 4×10^-3^ | 0.8 ± 1.3 | 0.5 ± 0.5 |  |
| Thallium (Tl) | 0.9 ± 1.0 | < 2 | 5.9 ± 0.9 | 9.5 ± 0.8 | 13.1 ± 1.3 | 9.6 ± 1.7 | 1.0 ± 0.6 | 1.1 ± 0.8 | 2.6 ± 0.8 |  |
| Thorium (Th) | 0.5 ± 0.9 | < 2 | 0.2 ± 0.3 | 0.5 ± 0.5 | 2.7 ± 1.3 | 1.1 ± 0.4 | 4.42×10^-2^ ± 1.1×10^-3^ | 0.2 ± 0.2 | 1.3 ± 0.6 |  |
| Tin (Sn) | 0.3 ± 0.4 | < 2 | 1.08 ± 0.14 | 1.6 ± 0.6 | 2.3 ± 1.2 | 0.85 ± 0.12 | 7.6×10^-2^ ± 3×10^-3^ | 0.3 ± 0.5 | 0.7 ± 0.5 |  |
| Tin (^117m^Sn) |  | 1.6 ± 1.2 | 3 ± 2 | 4.0 ± 0.7 | 3 ± 2 | 4 ± 3 | 2 ± 3 | 5 ± 4 | 10 ± 4 | 1.2 ± 1.3 |
| Uranium (U) | 0.7 ± 1.0 | < 2 | 0.71 ± 0.19 | 1.3 ± 0.5 | 2.9 ± 1.1 | 1.0 ± 0.7 | 2×10^-2^ ± 4×10^-2^ | 0.2 ± 0.2 | 1.2 ± 0.3 |  |
| Ytterbium (Yb) | 0.8 ± 1.2 | < 2 | 0.82 ± 0.10 | 2.0 ± 0.9 | 2.9 ± 1.4 | 1.4 ± 0.3 | 1.21×10^-2^ ± 4×10^-4^ | 0.7 ± 1.0 | 0.8 ± 0.3 |  |
| Yttrium (Y) | 0.3 ± 0.5 | < 2 | 0.23 ± 0.09 | 1.3 ± 0.6 | 2.1 ± 1.5 | 1.6 ± 1.0 | 1.4×10^-3^ ± 1.7×10^-3^ | 0.6 ± 0.8 | 0.4 ± 0.4 |  |
| Zinc (Zn) | 0.76 ± 0.06 | < 2 | 0.8705 ± 0.0005 | 0.82 ± 0.04 | 1.4 ± 0.7 | 0.88 ± 0.03 | 0.96 ± 0.05 | 0.95 ± 0.02 | 1.129 ± 0.018 |  |
| Zirconium (Zr) | 0.3 ± 0.4 | < 2 | 0.9 ± 0.5 | 1.0 ± 0.8 | 0.9 ± 1.1 | 1.2 ± 0.5 | 5.58×10^-2^ ± 1.3×10^-3^ | 0.8 ± 1.1 | 0.2 ± 0.3 |  |

^[a]^Unless noted otherwise, all K_D_ values were acquired using stable elements from a multi-element standard. ^[b]^For K_D_ determined using the multi-element standard the total procedural blank gave higher readings than the samples themselves. Hence a maximum K_D_ value was established based on the highest readings and is denoted as “< n”.

**Table S10**. Distribution coefficient (K_D_) values for DPE-300 resin.

| Element^[a]^ | Distribution coefficients (K_D_) in hydrochloric acid (K_D_ ± SD, n = 3) | | | | | | | | | |
| --- | --- | --- | --- | --- | --- | --- | --- | --- | --- | --- |
|  | 0.01 M | 0.1 M | 0.5 M | 1 M | 2 M | 3 M | 6 M | 8 M | 10 M | 12 M |
| Aluminium (Al) | 0.4 ± 0.2 | 0.6 ± 0.2 | 9 ± 14 | 1.081 ± 0.010 | 0.11 ± 0.04 | 0.99 ± 0.04 | 0.85 ± 0.04 | 1.067 ± 0.019 | 1.32 ± 0.04 |  |
| Antimony (Sb) | 4.24×10^-3^ ± 1.1×10^-4^ | 3.82×10^-3^ ± 1.3×10^-4^ | 4.41×10^-3^ ± 1.6×10^-4^ | 4.92×10^-3^ ± 1.7×10^-4^ | 1.52×10^-2^ ± 6×10^-4^ | 2.1×10^-2^ ± 6×10^-3^ | 2.9 ± 0.3 | 42 ± 2 | 1800 ± 300 |  |
| Antimony (Sb, single-element standard) |  |  |  |  |  |  | 32 ± 3 | 108 ± 19 | 1430 ± 150 | 290 ± 80 |
| Antimony (^120m^Sb) |  | 10.1 ± 1.5 | 25 ± 4 | 33 ± 6 | 33.5 ± 0.6 | 33 ± 2 | 31 ± 3 | 63 ± 2 | 290 ± 20 | 800 ± 20 |
| Barium (Ba) | 5.29×10^-3^ ± 1.9×10^-4^ | 3.41×10^-3^ ± 9×10^-5^ | 3.64×10^-3^ ± 8×10^-5^ | 4.71×10^-3^ ± 1.1×10^-4^ | 1 ± 2 | 0.4 ± 0.3 | 0.4 ± 0.2 | 4.81×10^-3^ ± 1.4×10^-4^ | 0.2 ± 0.2 |  |
| Bismuth (Bi) | 1.43 ± 0.16 | 0.50 ± 0.06 | 0.65 ± 0.06 | 0.72 ± 0.07 | 1.6 ± 1.4 | 0.6 ± 0.2 | 1.4 ± 0.7 | 1.2 ± 0.3 | 0.7 ± 0.8 |  |
| Cerium (Ce) | 7.07×10^-3^ ± 1.5×10^-4^ | 5.48×10^-3^ ± 1.3×10^-4^ | 5.31×10^-3^ ± 1.2×10^-4^ | 6.37×10^-3^ ± 1.6×10^-4^ | 1.1 ± 1.9 | 6.9×10^-3^ ± 3×10^-4^ | 0.5 ± 0.3 | 0.4 ± 0.3 | 0.2 ± 0.3 |  |
| Cesium (Cs) | 4.76×10^-3^ ± 1.7×10^-4^ | 0.08 ± 0.14 | 4.79×10^-3^ ± 1.8×10^-4^ | 5.07×10^-3^ ± 1.9×10^-4^ | 1.38×10^-2^ ± 4×10^-4^ | 7×10^-2^ ± 6×10^-2^ | 0.4 ± 0.4 | 0.4 ± 0.3 | 0.1 ± 0.2 |  |
| Cobalt (Co) | 4.90×10^-3^ ± 1.9×10^-4^ | 3.78×10^-3^ ± 8×10^-5^ | 0.3 ± 0.3 | 4.38×10^-3^ ± 1.6×10^-4^ | 1.1 ± 1.5 | 4.60×10^-3^ ± 1.8×10^-4^ | 0.7 ± 0.4 | 0.5 ± 0.3 | 0.07 ± 0.12 |  |
| Copper (Cu) | 4.34×10^-2^ ± 3×10^-4^ | 0.7 ± 0.3 | 3.9×10^-2^ ± 6×10^-3^ | 3.20×10^-2^ ± 5×10^-4^ | 2 ± 2 | 0.10 ± 0.04 | 3.6×10^-2^ ± 1.3×10^-2^ | 0.6 ± 0.6 | 5.23×10^-2^ ± 3×10^-4^ |  |
| Erbium (Er) | 1.38×10^-3^ ± 5×10^-5^ | 1.65×10^-3^ ± 3×10^-5^ | 1.47×10^-3^ ± 4×10^-5^ | 1.33×10^-3^ ± 3×10^-5^ | 0.8 ± 1.4 | 2.07×10^-3^ ± 9×10^-5^ | 0.80 ± 0.06 | 0.27 ± 0.06 | 0.4 ± 0.3 |  |
| Europium (Eu) | 1.01×10^-3^ ± 3×10^-5^ | 6.7×10^-4^ ± 2×10^-5^ | 8.3×10^-4^ ± 4×10^-5^ | 1.01×10^-3^ ± 3×10^-5^ | 0.9 ± 1.6 | 8.4×10^-4^ ± 2×10^-5^ | 0.6 ± 0.5 | 0.7 ± 0.5 | 0.7 ± 0.5 |  |
| Gallium (Ga) | 0.2 ± 0.4 | 0.2 ± 0.3 | 0.08 ± 0.11 | 3.53×10^-3^ ± 1.9×10^-4^ | 1.5 ± 1.8 | 0.3 ± 0.4 | 22.9 ± 1.2 | 51.4 ± 1.8 | 41.2 ± 1.4 |  |
| Hafnium (Hf) | 2.43×10^-2^ ± 1.1×10^-3^ | 2.18×10^-2^ ± 9×10^-4^ | 2.12×10^-2^ ± 3×10^-4^ | 2.09×10^-2^ ± 7×10^-4^ | 0.7 ± 1.2 | 2.44×10^-2^ ± 9×10^-4^ | 1.1 ± 0.3 | 0.13 ± 0.19 | 0.4 ± 0.4 |  |
| Lanthanum (La) | 3.35×10^-3^ ± 8×10^-5^ | 2.71×10^-3^ ± 6×10^-5^ | 5×10^-2^ ± 8×10^-2^ | 3.28×10^-3^ ± 4×10^-5^ | 1 ± 2 | 2.76×10^-3^ ± 8×10^-5^ | 0.5 ± 0.3 | 0.5 ± 0.5 | 0.6 ± 0.3 |  |
| Lead (Pb) | 0.55 ± 0.06 | 0.50 ± 0.05 | 0.53 ± 0.05 | 0.50 ± 0.04 | 1.4 ± 1.6 | 0.54 ± 0.03 | 1.2 ± 1.2 | 0.56 ± 0.12 | 0.67 ± 0.19 |  |
| Lutetium (Lu) | 1.73×10^-4^ ± 6×10^-6^ | 3.97×10^-4^ ± 1.8×10^-5^ | 2.93×10^-4^ ± 3×10^-6^ | 4.88×10^-4^ ± 1.8×10^-5^ | 0.9 ± 1.6 | 6.59×10^-4^ ± 1.8×10^-5^ | 0.88 ± 0.18 | 0.2 ± 0.2 | 0.8 ± 0.5 |  |
| Molybdenum (Mo) | 0.126 ± 0.003 | 8×10^-2^ ± 6×10^-2^ | 0.3 ± 0.3 | 0.115 ± 0.005 | 0.7 ± 1.0 | 0.17 ± 0.09 | 0.4 ± 0.4 | 0.8 ± 0.9 | 0.5 ± 0.7 |  |
| Neodymium (Nd) | 3.32×10^-3^ ± 1.1×10^-4^ | 2.59×10^-3^ ± 8×10^-5^ | 3.19×10^-3^ ± 6×10^-5^ | 3.06×10^-3^ ± 1.0×10^-4^ | 1 ± 2 | 2.57×10^-3^ ± 9×10^-5^ | 0.3 ± 0.3 | 0.2 ± 0.3 | 0.2 ± 0.3 |  |
| Nickel (Ni) | 0.09 ± 0.15 | 4.18×10^-3^ ± 1.1×10^-4^ | 0.2 ± 0.3 | 5.89×10^-3^ ± 1.3×10^-4^ | 1.1 ± 0.8 | 0.72 ± 0.02 | 0.5 ± 0.7 | 0.15 ± 0.15 | 1.43×10^-2^ ± 2×10^-4^ |  |
| Niobium (Nb) | 4.5×10^-2^ ± 2×10^-3^ | 4.10×10^-2^ ± 1.6×10^-3^ | 2.84×10^-2^ ± 9×10^-4^ | 1.63×10^-2^ ± 3×10^-4^ | 1.63×10^-2^ ± 1.0×10^-3^ | 1.59×10^-2^ ± 9×10^-4^ | 1.27×10^-2^ ± 5×10^-4^ | 8×10^-2^ ± 4×10^-2^ | 0.4 ± 0.5 |  |
| Rhenium (Re) | 1.28×10^-4^ ± 3×10^-6^ | 4.45×10^-5^ ± 1.4×10^-6^ | 6.30×10^-5^ ± 5×10^-7^ | 1.76×10^-4^ ± 6×10^-6^ | 0.7 ± 1.2 | 0.11 ± 0.19 | 1.0 ± 0.6 | 0.6 ± 0.5 | 0.7 ± 0.3 |  |
| Rubidium (Rb) | 0.3 ± 0.3 | 0.1 ± 0.2 | 2.96×10^-3^ ± 1.2×10^-4^ | 3.07×10^-3^ ± 1.0×10^-4^ | 0.7 ± 1.1 | 0.4 ± 0.6 | 0.2 ± 0.3 | 3.08×10^-3^ ± 1.0×10^-4^ | 4.18×10^-3^ ± 1.0×10^-4^ |  |
| Scandium (Sc) | 7.85×10^-2^ ± 1.1×10^-3^ | 6.9×10^-2^ ± 2×10^-3^ | 7.0×10^-2^ ± 2×10^-3^ | 6.31×10^-2^ ± 5×10^-4^ | 1.0 ± 1.7 | 6.6×10^-2^ ± 2×10^-3^ | 0.2 ± 0.3 | 0.3 ± 0.3 | 6.23×10^-2^ ± 8×10^-4^ |  |
| Silver (Ag) | 1.7 ± 1.6 | 8.8×10^-2^ ± 4×10^-3^ | 0.2 ± 0.2 | 2.56×10^-2^ ± 1.1×10^-3^ | 0.6 ± 1.0 | 1.70×10^-2^ ± 7×10^-4^ | 0.1 ± 0.2 | 0.1 ± 0.2 | 0.07 ± 0.11 |  |
| Strontium (Sr) | 1.78×10^-2^ ± 5×10^-4^ | 9.6×10^-3^ ± 3×10^-4^ | 1.206×10^-2^ ± 1.0×10^-4^ | 1.69×10^-2^ ± 6×10^-4^ | 1.7 ± 0.8 | 1.0 ± 0.8 | 0.9 ± 0.5 | 1.68×10^-2^ ± 6×10^-4^ | 4×10^-2^ ± 4×10^-2^ |  |
| Thallium (Tl) | 3.30×10^-2^ ± 3×10^-4^ | 0.8 ± 0.4 | 7.7 ± 0.6 | 8.8 ± 1.0 | 13 ± 3 | 10.9 ± 1.2 | 4.3 ± 0.4 | 2.5 ± 0.3 | 1.4 ± 0.5 |  |
| Thorium (Th) | 3.97×10^-2^ ± 1.0×10^-3^ | 3.65×10^-2^ ± 1.4×10^-3^ | 4.01×10^-2^ ± 1.5×10^-3^ | 3.96×10^-2^ ± 1.6×10^-3^ | 1.1 ± 1.9 | 0.2 ± 0.3 | 0.7 ± 0.5 | 0.16 ± 0.13 | 4.71×10^-2^ ± 8×10^-4^ |  |
| Tin (Sn) | 0.125 ± 0.002 | 0.116 ± 0.005 | 0.123 ± 0.005 | 0.1098 ± 0.0017 | 0.8 ± 1.2 | 0.106 ± 0.004 | 0.2 ± 0.2 | 0.14 ± 0.11 | 0.15 ± 0.12 |  |
| Tin (^117m^Sn) |  | 0.8 ± 1.2 | 1.0 ± 1.5 | 1.2 ± 1.0 | 0.9 ± 1.3 | 3.7 ± 0.6 | 5.1 ± 1.7 | 4.7 ± 1.3 | 2 ± 2 | 10 ± 10 |
| Uranium (U) | 9.0×10^-4^ ± 4×10^-5^ | 3×10^-2^ ± 5×10^-2^ | 4×10^-2^ ± 7×10^-2^ | 7.26×10^-4^ ± 1.9×10^-5^ | 0.9 ± 1.5 | 7.5×10^-4^ ± 2×10^-5^ | 0.07 ± 0.13 | 0.2 ± 0.2 | 0.15 ± 0.14 |  |
| Ytterbium (Yb) | 1.83×10^-3^ ± 5×10^-5^ | 1.14×10^-3^ ± 4×10^-5^ | 3.56×10^-3^ ± 5×10^-5^ | 1.742×10^-3^ ± 8×10^-6^ | 0.7 ± 1.3 | 3.39×10^-3^ ± 1.7×10^-4^ | 1.1 ± 0.3 | 0.6 ± 0.6 | 0.7 ± 0.3 |  |
| Yttrium (Y) | 9.8×10^-4^ ± 2×10^-5^ | 0.3 ± 0.6 | 0.1 ± 0.2 | 6.6×10^-4^ ± 3×10^-5^ | 1.2 ± 1.7 | 0.5 ± 0.5 | 4×10^-2^ ± 7×10^-2^ | 0.07 ± 0.12 | 0.5 ± 0.7 |  |
| Zinc (Zn) | 0.80 ± 0.04 | 0.87 ± 0.03 | 0.962 ± 0.017 | 0.97 ± 0.04 | 1.14 ± 0.11 | 0.90 ± 0.05 | 1.087 ± 0.016 | 1.04 ± 0.05 | 1.06 ± 0.06 |  |
| Zirconium (Zr) | 3.56×10^-2^ ± 1.2×10^-3^ | 0.2 ± 0.3 | 3.48×10^-2^ ± 1.3×10^-3^ | 3.24×10^-2^ ± 1.8×10^-3^ | 1.0 ± 1.4 | 0.8 ± 0.8 | 0.14 ± 0.14 | 5.88×10^-2^ ± 1.5×10^-3^ | 0.3 ± 0.5 |  |

^[a]^Unless noted otherwise, all K_D_ values were acquired using stable elements from a multi-element standard.

**Table S11**. Distribution coefficient (K_D_) values for DOE-300 resin.

| Element^[a]^ | Distribution coefficients (K_D_) in hydrochloric acid (K_D_ ± SD, n = 3) | | | | | | | | | |
| --- | --- | --- | --- | --- | --- | --- | --- | --- | --- | --- |
|  | 0.01 M | 0.1 M | 0.5 M | 1 M | 2 M | 3 M | 6 M | 8 M | 10 M | 12 M |
| Aluminium (Al) | 1.0 ± 0.8 | 0.9 ± 0.3 | 2.7 ± 1.7 | 1.1 ± 0.6 | 1.28 ± 0.02 | 0.9 ± 0.4 | 0.7 ± 0.6 | 1.29 ± 0.06 | 1.07 ± 0.07 |  |
| Antimony (Sb) | 6.54×10^-2^ ± 1.3×10^-3^ | 6.04×10^-3^ ± 1.5×10^-4^ | 0.3 ± 0.3 | 0.5 ± 0.9 | 8.07×10^-3^ ± 1.4×10^-4^ | 0.27 ± 0.18 | 1.1 ± 0.9 | 27.6 ± 0.5 | 970 ± 20 |  |
| Antimony (Sb, single-element standard) |  |  |  |  |  |  | 13 ± 11 | 50 ± 4 | 230 ± 160 | 290 ± 60 |
| Antimony (^120m^Sb) |  | 14 ± 4 | 22 ± 2 | 50 ± 16 | 32 ± 12 | 26.8 ± 1.0 | 100 ± 30 | 73 ± 10 | 1500 ± 200 | 1030 ± 50 |
| Barium (Ba) | 3.92×10^-3^ ± 6×10^-5^ | 4.96×10^-3^ ± 7×10^-5^ | 0.5 ± 0.5 | 1.0 ± 1.7 | 0.7 ± 0.5 | 0.2 ± 0.3 | 0.3 ± 0.3 | 5×10^-2^ ± 4×10^-2^ | 0.3 ± 0.2 |  |
| Bismuth (Bi) | 2.56 ± 0.09 | 1.08 ± 0.13 | 1.26 ± 0.04 | 2.2 ± 1.8 | 0.4 ± 0.6 | 0.7 ± 0.6 | 0.6 ± 0.3 | 1.2 ± 0.3 | 0.6 ± 0.2 |  |
| Cerium (Ce) | 8.2×10^-3^ ± 3×10^-4^ | 8.28×10^-3^ ± 6×10^-5^ | 0.21 ± 0.18 | 1 ± 2 | 0.07 ± 0.10 | 0.2 ± 0.4 | 0.2 ± 0.3 | 0.11 ± 0.18 | 0.4 ± 0.2 |  |
| Cesium (Cs) | 4.56×10^-3^ ± 1.2×10^-4^ | 5.77×10^-3^ ± 1.8×10^-4^ | 0.4 ± 0.3 | 1 ± 2 | 0.1 ± 0.2 | 0.5 ± 0.6 | 0.4 ± 0.4 | 5.26×10^-3^ ± 1.8×10^-4^ | 0.2 ± 0.4 |  |
| Cobalt (Co) | 4.09×10^-3^ ± 1.5×10^-4^ | 5.25×10^-3^ ± 1.1×10^-4^ | 1.3 ± 0.8 | 1.0 ± 1.8 | 0.5 ± 0.7 | 0.5 ± 0.9 | 0.5 ± 0.5 | 3.86×10^-3^ ± 1.1×10^-4^ | 1.1 ± 0.6 |  |
| Copper (Cu) | 3.10×10^-2^ ± 3×10^-4^ | 3.34×10^-2^ ± 6×10^-4^ | 0.6 ± 0.5 | 1 ± 2 | 2.74×10^-2^ ± 5×10^-4^ | 0.2 ± 0.3 | 1.0 ± 1.0 | 3.34×10^-2^ ± 1.2×10^-3^ | 0.5 ± 0.5 |  |
| Erbium (Er) | 9.38×10^-3^ ± 1.0×10^-4^ | 3.55×10^-3^ ± 1.0×10^-4^ | 0.2 ± 0.3 | 2 ± 3 | 0.2 ± 0.4 | 0.9 ± 0.4 | 0.6 ± 0.4 | 0.3 ± 0.4 | 0.6 ± 0.7 |  |
| Europium (Eu) | 1.55×10^-3^ ± 4×10^-5^ | 1.74×10^-3^ ± 4×10^-5^ | 5×10^-2^ ± 6×10^-2^ | 0.7 ± 1.2 | 2.10×10^-3^ ± 3×10^-5^ | 0.4 ± 0.6 | 0.5 ± 0.5 | 1.33×10^-3^ ± 4×10^-5^ | 0.2 ± 0.2 |  |
| Gallium (Ga) | 4.31×10^-3^ ± 1.4×10^-4^ | 0.2 ± 0.3 | 4.74×10^-3^ ± 3×10^-5^ | 1.0 ± 1.7 | 4.93×10^-3^ ± 1.6×10^-4^ | 5.08×10^-3^ ± 1.7×10^-4^ | 11.6 ± 1.2 | 27.7 ± 1.0 | 26.6 ± 0.7 |  |
| Hafnium (Hf) | 4.82×10^-2^ ± 4×10^-4^ | 5.30×10^-2^ ± 1.4×10^-3^ | 0.3 ± 0.2 | 1 ± 2 | 4.2×10^-2^ ± 9×10^-3^ | 0.5 ± 0.5 | 0.7 ± 0.5 | 4.68×10^-2^ ± 1.5×10^-3^ | 0.5 ± 0.5 |  |
| Lanthanum (La) | 3.91×10^-3^ ± 5×10^-5^ | 4.095×10^-3^ ± 1.9×10^-5^ | 0.8 ± 0.8 | 0.8 ± 1.5 | 4×10^-2^ ± 6×10^-2^ | 0.5 ± 0.4 | 0.3 ± 0.5 | 4×10^-2^ ± 7×10^-2^ | 0.2 ± 0.2 |  |
| Lead (Pb) | 0.99 ± 0.07 | 0.97 ± 0.08 | 0.82 ± 0.15 | 1.8 ± 1.6 | 0.91 ± 0.06 | 1.1 ± 0.4 | 0.5 ± 0.4 | 1.1 ± 0.7 | 1.3 ± 1.2 |  |
| Lutetium (Lu) | 5.09×10^-4^ ± 6×10^-6^ | 1.134×10^-3^ ± 1.7×10^-5^ | 0.9 ± 0.3 | 0.9 ± 1.6 | 4×10^-2^ ± 6×10^-2^ | 0.5 ± 0.5 | 0.4 ± 0.7 | 0.08 ± 0.13 | 2.0 ± 0.3 |  |
| Molybdenum (Mo) | 0.115 ± 0.005 | 0.147 ± 0.009 | 0.8 ± 0.7 | 0.8 ± 1.2 | 0.15 ± 0.04 | 0.7 ± 0.9 | 0.13 ± 0.04 | 0.104 ± 0.004 | 1.8 ± 0.6 |  |
| Neodymium (Nd) | 3.85×10^-3^ ± 3×10^-5^ | 3.63×10^-3^ ± 7×10^-5^ | 0.7 ± 0.4 | 0.8 ± 1.4 | 3.301×10^-3^ ± 1.6×10^-5^ | 0.5 ± 0.3 | 0.2 ± 0.3 | 5×10^-2^ ± 8×10^-2^ | 0.2 ± 0.3 |  |
| Nickel (Ni) | 6.8×10^-3^ ± 3×10^-4^ | 8.71×10^-3^ ± 5×10^-5^ | 1.1 ± 1.0 | 1 ± 3 | 8.5×10^-3^ ± 3×10^-4^ | 0.61 ± 0.13 | 0.9 ± 0.7 | 1.245×10^-2^ ± 1.6×10^-4^ | 0.3 ± 0.5 |  |
| Niobium (Nb) | 8.93×10^-2^ ± 1.3×10^-3^ | 0.115 ± 0.004 | 0.16 ± 0.13 | 0.8 ± 1.2 | 9.1×10^-2^ ± 3×10^-3^ | 0.4 ± 0.3 | 9.9×10^-2^ ± 2×10^-3^ | 9.5×10^-2^ ± 3×10^-3^ | 1.1 ± 0.7 |  |
| Rhenium (Re) | 1.36×10^-4^ ± 3×10^-6^ | 4.80×10^-4^ ± 5×10^-6^ | 0.9 ± 0.6 | 1 ± 2 | 0.16 ± 0.14 | 0.6 ± 0.6 | 0.2 ± 0.3 | 0.1 ± 0.2 | 1.0 ± 0.3 |  |
| Rubidium (Rb) | 6.39×10^-2^ ± 7×10^-4^ | 7.2×10^-2^ ± 4×10^-3^ | 0.8 ± 0.4 | 0.9 ± 1.5 | 7.24×10^-2^ ± 1.6×10^-3^ | 0.3 ± 0.4 | 0.8 ± 0.3 | 0.15 ± 0.14 | 7.4×10^-2^ ± 3×10^-3^ |  |
| Scandium (Sc) | 0.3 ± 0.3 | 0.22 ± 0.13 | 0.5 ± 0.3 | 1.1 ± 1.6 | 0.4 ± 0.3 | 0.6 ± 0.8 | 0.12 ± 0.03 | 0.23 ± 0.18 | 0.17 ± 0.07 |  |
| Silver (Ag) | 5.8 ± 0.3 | 0.127 ± 0.009 | 0.5 ± 0.6 | 1.0 ± 1.6 | 2.54×10^-2^ ± 5×10^-4^ | 0.8 ± 0.8 | 0.5 ± 0.8 | 1.42×10^-2^ ± 5×10^-4^ | 0.3 ± 0.5 |  |
| Strontium (Sr) | 2.302×10^-2^ ± 1.1×10^-4^ | 1.92×10^-2^ ± 7×10^-4^ | 1.4 ± 1.1 | 1.0 ± 1.7 | 0.2 ± 0.2 | 0.2 ± 0.2 | 5×10^-2^ ± 4×10^-2^ | 1.28×10^-2^ ± 6×10^-4^ | 0.3 ± 0.3 |  |
| Thallium (Tl) | 7.00×10^-2^ ± 1.2×10^-3^ | 0.2 ± 0.2 | 4.3 ± 1.1 | 6 ± 4 | 4.45 ± 0.18 | 7.0 ± 0.7 | 2.3 ± 0.5 | 0.7 ± 0.9 | 0.5 ± 0.7 |  |
| Thorium (Th) | 0.149 ± 0.002 | 0.154 ± 0.003 | 0.6 ± 0.5 | 1.1 ± 1.7 | 0.1431 ± 0.0017 | 0.5 ± 0.4 | 0.144 ± 0.015 | 0.125 ± 0.006 | 0.134 ± 0.003 |  |
| Tin (Sn) | 0.1199 ± 0.0011 | 0.133 ± 0.004 | 0.9 ± 0.5 | 0.6 ± 0.8 | 0.16 ± 0.09 | 0.6 ± 0.7 | 0.3 ± 0.4 | 7.5×10^-2^ ± 4×10^-3^ | 9.5×10^-2^ ± 4×10^-3^ |  |
| Tin (^117m^Sn) |  | 10 ± 20 | 17 ± 2 | 9 ± 5 | 16 ± 8 | 20.4 ± 1.0 | 20.5 ± 1.4 | 13 ± 6 | 17 ± 4 | 21 ± 8 |
| Uranium (U) | 1.67×10^-3^ ± 5×10^-5^ | 1.74×10^-3^ ± 2×10^-5^ | 0.15 ± 0.15 | 0.6 ± 1.0 | 9.40×10^-4^ ± 1.3×10^-5^ | 0.3 ± 0.4 | 8.0×10^-4^ ± 3×10^-5^ | 1.01×10^-3^ ± 4×10^-5^ | 0.3 ± 0.3 |  |
| Ytterbium (Yb) | 0.31 ± 0.13 | 0.108 ± 0.003 | 0.6 ± 0.5 | 1.2 ± 1.7 | 0.1138 ± 0.0009 | 0.3 ± 0.3 | 0.38 ± 0.16 | 6.3×10^-2^ ± 2×10^-3^ | 1.1 ± 0.2 |  |
| Yttrium (Y) | 9.8×10^-4^ ± 3×10^-5^ | 1.41×10^-3^ ± 6×10^-5^ | 1.2 ± 1.1 | 0.9 ± 1.5 | 2.30×10^-3^ ± 3×10^-5^ | 0.3 ± 0.5 | 1.463×10^-3^ ± 1.2×10^-5^ | 1.01×10^-3^ ± 3×10^-5^ | 0.10 ± 0.13 |  |
| Zinc (Zn) | 0.794 ± 0.003 | 0.87 ± 0.05 | 0.94 ± 0.06 | 0.79 ± 0.08 | 0.93 ± 0.04 | 0.91 ± 0.04 | 0.990 ± 0.019 | 0.96 ± 0.04 | 0.97 ± 0.04 |  |
| Zirconium (Zr) | 6.10×10^-2^ ± 1.2×10^-3^ | 5.81×10^-2^ ± 7×10^-4^ | 0.9 ± 0.8 | 1.2 ± 1.9 | 7.12×10^-2^ ± 1.1×10^-3^ | 1.0 ± 0.3 | 0.6 ± 0.4 | 9.3×10^-2^ ± 2×10^-3^ | 0.105 ± 0.005 |  |

^[a]^Unless noted otherwise, all K_D_ values were acquired using stable elements from a multi-element standard.

**Table S12**. Distribution coefficient (K_D_) values for TK401-300 resin.

| Element^[a]^ | Distribution coefficients (K_D_) in hydrochloric acid (K_D_ ± SD, n = 3) | | | | | | | | | |
| --- | --- | --- | --- | --- | --- | --- | --- | --- | --- | --- |
|  | 0.01 M | 0.1 M | 0.5 M | 1 M | 2 M | 3 M | 6 M | 8 M | 10 M | 12 M |
| Aluminium (Al) | 1.2 ± 0.7 | 0.9 ± 0.2 | 0.78 ± 0.03 | 106 ± 3 | 1.9 ± 0.3 | 3.33 ± 0.04 | 0.176 ± 0.014 | 1.42 ± 0.08 | 1.14 ± 0.12 |  |
| Antimony (Sb) | 3.4 ± 0.2 | 5.7×10^-2^ ± 2×10^-3^ | 5.43×10^-2^ ± 1.1×10^-3^ | 1.4 ± 1.0 | 0.740 ± 0.012 | 1.063×10^-2^ ± 1.9×10^-4^ | 1.40×10^-2^ ± 3×10^-4^ | 3.6 ± 0.6 | 10 ± 9 |  |
| Antimony (Sb, single-element standard) |  |  |  |  |  |  | 1.99 ± 0.04 | 10 ± 2 | 5 ± 9 | 1.99 ± 0.02 |
| Antimony (^120m^Sb) |  | 0.4 ± 0.3 | 7 ± 3 | 5.7 ± 0.2 | 8 ± 2 | 10 ± 4 | 13.4 ± 1.9 | 13.4 ± 0.2 | 21 ± 5 | 36 ± 5 |
| Barium (Ba) | 7.39×10^-2^ ± 1.5×10^-3^ | 3.57×10^-3^ ± 7×10^-5^ | 7.3 ± 0.9 | 0.166 ± 0.003 | 0.12 ± 0.08 | 4.35×10^-3^ ± 6×10^-5^ | 0.5 ± 0.4 | 1.1 ± 1.1 | 0.4 ± 0.4 |  |
| Bismuth (Bi) | 7.1 ± 1.2 | 0.22 ± 0.02 | 0.27 ± 0.02 | 0.7 ± 0.7 | 0.4 ± 0.2 | 0.397 ± 0.018 | 0.6 ± 0.4 | 0.9 ± 0.6 | 0.9 ± 0.6 |  |
| Cerium (Ce) | 2.91×10^-2^ ± 3×10^-4^ | 5.20×10^-3^ ± 7×10^-5^ | 0.2 ± 0.3 | 0.8 ± 1.0 | 0.08 ± 0.11 | 1.159×10^-2^ ± 1.7×10^-4^ | 0.3 ± 0.4 | 1.2 ± 0.7 | 0.7 ± 0.6 |  |
| Cesium (Cs) | 1.97 ± 0.11 | 4.56×10^-3^ ± 1.4×10^-4^ | 0.2 ± 0.3 | 0.9 ± 0.5 | 0.7 ± 0.6 | 1.115×10^-2^ ± 1.1×10^-4^ | 6.32×10^-3^ ± 1.1×10^-4^ | 0.7 ± 0.8 | 0.2 ± 0.4 |  |
| Cobalt (Co) | 2.35×10^-2^ ± 5×10^-4^ | 2.88×10^-3^ ± 8×10^-5^ | 2.67×10^-3^ ± 9×10^-5^ | 7.0 ± 1.7 | 0.3 ± 0.3 | 4.53×10^-3^ ± 7×10^-5^ | 0.08 ± 0.14 | 0.7 ± 0.8 | 0.7 ± 0.8 |  |
| Copper (Cu) | 4.99×10^-2^ ± 6×10^-4^ | 2.05×10^-2^ ± 9×10^-4^ | 4.1 ± 1.8 | 0.108 ± 0.006 | 0.2 ± 0.3 | 6.04×10^-2^ ± 6×10^-4^ | 0.33 ± 0.23 | 1.0 ± 1.1 | 0.2 ± 0.4 |  |
| Erbium (Er) | 3.04×10^-2^ ± 7×10^-4^ | 2.45×10^-3^ ± 6×10^-5^ | 2.407×10^-3^ ± 1.5×10^-5^ | 0.7 ± 1.0 | 2.55×10^-2^ ± 3×10^-4^ | 2.34×10^-3^ ± 3×10^-5^ | 0.11 ± 0.18 | 0.6 ± 0.7 | 0.4 ± 0.3 |  |
| Europium (Eu) | 2.64×10^-2^ ± 6×10^-4^ | 8.51×10^-4^ ± 1.7×10^-5^ | 9.6×10^-4^ ± 3×10^-5^ | 0.3 ± 0.5 | 1.54×10^-2^ ± 3×10^-4^ | 1.104×10^-3^ ± 1.7×10^-5^ | 0.1 ± 0.3 | 0.6 ± 0.6 | 0.2 ± 0.2 |  |
| Gallium (Ga) | 2.37×10^-2^ ± 6×10^-4^ | 1.97×10^-3^ ± 5×10^-5^ | 0.5 ± 0.7 | 0.5 ± 0.8 | 0.13 ± 0.16 | 2.42×10^-3^ ± 5×10^-5^ | 1.294×10^-2^ ± 1.5×10^-4^ | 1.6 ± 1.2 | 0.5 ± 0.5 |  |
| Hafnium (Hf) | 1.96×10^-2^ ± 4×10^-4^ | 1.14×10^-2^ ± 4×10^-4^ | 1.166×10^-2^ ± 1.8×10^-4^ | 0.8 ± 1.1 | 6×10^-2^ ± 6×10^-2^ | 1.241×10^-2^ ± 1.9×10^-4^ | 0.12 ± 0.18 | 1.0 ± 0.6 | 0.4 ± 0.3 |  |
| Lanthanum (La) | 1.97×10^-2^ ± 4×10^-4^ | 2.43×10^-3^ ± 6×10^-5^ | 0.2 ± 0.3 | 1.0 ± 1.0 | 0.19 ± 0.19 | 0.11 ± 0.19 | 0.7 ± 0.3 | 1.4 ± 1.0 | 0.5 ± 0.4 |  |
| Lead (Pb) | 0.34 ± 0.03 | 0.32 ± 0.04 | 0.33 ± 0.04 | 0.457 ± 0.019 | 0.3 ± 0.3 | 0.34 ± 0.03 | 0.501 ± 0.010 | 0.44 ± 0.10 | 0.380 ± 0.017 |  |
| Lutetium (Lu) | 2.76×10^-2^ ± 6×10^-4^ | 3.06×10^-4^ ± 8×10^-6^ | 7.79×10^-4^ ± 9×10^-6^ | 0.6 ± 0.9 | 4.44×10^-2^ ± 7×10^-4^ | 6.146×10^-4^ ± 1.7×10^-6^ | 0.2 ± 0.4 | 0.8 ± 0.5 | 0.2 ± 0.3 |  |
| Molybdenum (Mo) | 4.58×10^-2^ ± 5×10^-4^ | 2.82×10^-2^ ± 1.0×10^-3^ | 5×10^-2^ ± 5×10^-2^ | 0.6 ± 0.9 | 8×10^-2^ ± 8×10^-2^ | 2.73×10^-2^ ± 5×10^-4^ | 2.85×10^-2^ ± 7×10^-4^ | 0.5 ± 0.5 | 2.71×10^-2^ ± 1.6×10^-3^ |  |
| Neodymium (Nd) | 3.788×10^-2^ ± 1.8×10^-4^ | 3.08×10^-3^ ± 1.0×10^-4^ | 2.92×10^-3^ ± 8×10^-5^ | 9.79×10^-2^ ± 1.0×10^-3^ | 0.14 ± 0.19 | 3.74×10^-3^ ± 7×10^-5^ | 5.84×10^-3^ ± 6×10^-5^ | 0.9 ± 0.7 | 0.2 ± 0.4 |  |
| Nickel (Ni) | 3.24×10^-2^ ± 8×10^-4^ | 5.42×10^-3^ ± 1.2×10^-4^ | 5.18×10^-3^ ± 1.7×10^-4^ | 0.8 ± 1.2 | 0.7 ± 0.3 | 0.4 ± 0.3 | 0.2 ± 0.3 | 0.6 ± 0.7 | 0.3 ± 0.3 |  |
| Niobium (Nb) | 5.49×10^-2^ ± 4×10^-4^ | 1.2 ± 0.7 | 6.5 ± 1.2 | 7.9×10^-2^ ± 1.3×10^-2^ | 0.11 ± 0.13 | 3.73×10^-2^ ± 4×10^-4^ | 4.33×10^-2^ ± 1.4×10^-3^ | 0.3 ± 0.4 | 0.5 ± 0.7 |  |
| Rhenium (Re) | 2.81×10^-2^ ± 8×10^-4^ | 1.77×10^-4^ ± 6×10^-6^ | 0.1 ± 0.2 | 0.5 ± 0.8 | 0.14 ± 0.13 | 9×10^-3^ ± 1.6×10^-2^ | 0.2 ± 0.3 | 0.8 ± 0.8 | 0.6 ± 0.6 |  |
| Rubidium (Rb) | 5.33×10^-2^ ± 1.6×10^-3^ | 6.6×10^-3^ ± 2×10^-4^ | 8.1×10^-3^ ± 2×10^-4^ | 0.6 ± 0.8 | 0.12 ± 0.16 | 8.14×10^-3^ ± 7×10^-5^ | 0.3 ± 0.6 | 0.8 ± 1.3 | 0.12 ± 0.19 |  |
| Scandium (Sc) | 9.1×10^-2^ ± 3×10^-3^ | 8.1×10^-2^ ± 3×10^-3^ | 7.9×10^-2^ ± 3×10^-3^ | 1.2 ± 0.9 | 0.14 ± 0.10 | 7.84×10^-2^ ± 1.1×10^-3^ | 0.2 ± 0.2 | 1.1 ± 1.1 | 1.1 ± 1.0 |  |
| Silver (Ag) | 2 ± 2 | 7.0 ± 0.9 | 0.5 ± 0.8 | 0.135 ± 0.014 | 3.37×10^-2^ ± 3×10^-4^ | 7.19×10^-3^ ± 9×10^-5^ | 3.5×10^-3^ ± 3×10^-4^ | 0.5 ± 0.8 | 1.1×10^-2^ ± 1.0×10^-2^ |  |
| Strontium (Sr) | 0.319 ± 0.011 | 1.00×10^-2^ ± 5×10^-4^ | 1.35×10^-2^ ± 4×10^-4^ | 0.44 ± 0.10 | 0.145 ± 0.002 | 1.38×10^-2^ ± 2×10^-4^ | 6.53×10^-2^ ± 8×10^-4^ | 0.5 ± 0.4 | 0.2 ± 0.3 |  |
| Thallium (Tl) | 4.96×10^-2^ ± 4×10^-4^ | 2.18×10^-2^ ± 5×10^-4^ | 1.4×10^-2^ ± 8×10^-3^ | 0.7 ± 1.1 | 5×10^-2^ ± 6×10^-2^ | 2.00×10^-2^ ± 5×10^-4^ | 6.44×10^-3^ ± 8×10^-5^ | 0.6 ± 0.4 | 0.3 ± 0.2 |  |
| Thorium (Th) | 2.97×10^-2^ ± 9×10^-4^ | 1.95×10^-2^ ± 8×10^-4^ | 2.06×10^-2^ ± 4×10^-4^ | 0.6 ± 0.9 | 3.03×10^-2^ ± 5×10^-4^ | 2.31×10^-2^ ± 5×10^-4^ | 0.17 ± 0.13 | 1.0 ± 0.8 | 0.3 ± 0.3 |  |
| Tin (Sn) | 0.1052 ± 0.0016 | 8.5×10^-2^ ± 4×10^-3^ | 8.7×10^-2^ ± 5×10^-3^ | 0.132 ± 0.005 | 8.9×10^-2^ ± 3×10^-3^ | 7.22×10^-2^ ± 3×10^-4^ | 6.64×10^-2^ ± 7×10^-4^ | 0.3 ± 0.3 | 6.2×10^-2^ ± 5×10^-3^ |  |
| Tin (^117m^Sn) |  | 0 | 0.8 ± 1.1 | 2.0 ± 1.8 | 0 | 0.4 ± 0.6 | 0 | 1 ± 2 | 0.8 ± 0.9 | 0.6 ± 0.9 |
| Uranium (U) | 1.62×10^-2^ ± 4×10^-4^ | 5.31×10^-4^ ± 1.2×10^-5^ | 7.32×10^-4^ ± 1.3×10^-5^ | 0.5 ± 0.7 | 9.2×10^-3^ ± 2×10^-4^ | 4.23×10^-4^ ± 9×10^-6^ | 0.1 ± 0.2 | 0.4 ± 0.4 | 0.2 ± 0.3 |  |
| Ytterbium (Yb) | 0.173 ± 0.003 | 1.41×10^-2^ ± 2×10^-4^ | 9.2×10^-3^ ± 2×10^-4^ | 0.148 ± 0.010 | 0.175 ± 0.002 | 1.78×10^-2^ ± 4×10^-4^ | 8.8×10^-2^ ± 4×10^-3^ | 1.0 ± 0.6 | 0.3 ± 0.4 |  |
| Yttrium (Y) | 3.23×10^-2^ ± 9×10^-4^ | 6.5×10^-4^ ± 3×10^-5^ | 0.2 ± 0.3 | 0.2 ± 0.3 | 1.74×10^-2^ ± 5×10^-4^ | 9.74×10^-4^ ± 7×10^-6^ | 3.33×10^-3^ ± 4×10^-5^ | 0.7 ± 0.5 | 1.31×10^-3^ ± 8×10^-5^ |  |
| Zinc (Zn) | 0.89 ± 0.03 | 1.00 ± 0.02 | 0.96 ± 0.02 | 1.25 ± 0.08 | 2.18 ± 0.06 | 0.92 ± 0.03 | 4.34 ± 0.08 | 1.11 ± 0.12 | 0.93 ± 0.17 |  |
| Zirconium (Zr) | 2.18×10^-2^ ± 4×10^-4^ | 1.39×10^-2^ ± 3×10^-4^ | 1.44×10^-2^ ± 5×10^-4^ | 1.0 ± 1.5 | 7×10^-2^ ± 7×10^-2^ | 1.90×10^-2^ ± 2×10^-4^ | 0.2 ± 0.4 | 1.0 ± 0.7 | 0.5 ± 0.4 |  |

^[a]^Unless noted otherwise, all K_D_ values were acquired using stable elements from a multi-element standard.

**Table S13**. Distribution coefficient (K_D_) values for CG300 resin support.

| Element^[a]^ | Distribution coefficients (K_D_) in hydrochloric acid (K_D_ ± SD, n = 3) | | | | | | | | |
| --- | --- | --- | --- | --- | --- | --- | --- | --- | --- |
|  | 0.01 M | 0.1 M | 0.5 M | 1 M | 2 M | 3 M | 6 M^[b]^ | 8 M | 10 M |
| Aluminium (Al) | 0.4 ± 0.3 | 0.67 ± 0.05 | 6 ± 2 | 0.96 ± 0.18 | 0.752 ± 0.016 | 1.22 ± 0.08 | < 2 | 1.30 ± 0.03 | 2.08 ± 0.17 |
| Antimony (Sb) | 9.69×10^-3^ ± 7×10^-5^ | 0.2 ± 0.4 | 2.3×10^-2^ ± 1.7×10^-2^ | 0.09 ± 0.13 | 1.101×10^-2^ ± 1.5×10^-4^ | 9×10^-3^ ± 2×10^-3^ | 1.2 ± 0.8 | 14.1 ± 0.7 | 2.02 ± 0.02 |
| Barium (Ba) | 4.068×10^-3^ ± 1.7×10^-5^ | 0.2 ± 0.4 | 6.16×10^-3^ ± 1.3×10^-4^ | 0.08 ± 0.13 | 0.06 ± 0.10 | 4.30×10^-3^ ± 6×10^-5^ | <2 | 4.59×10^-3^ ± 1.0×10^-4^ | 0.4 ± 0.7 |
| Bismuth (Bi) | 1.9 ± 0.3 | 0.70 ± 0.08 | 0.67 ± 0.08 | 0.86 ± 0.06 | 0.6 ± 0.3 | 0.78 ± 0.04 | < 2 | 1.4 ± 0.7 | 0.9 ± 1.0 |
| Cerium (Ce) | 7.29×10^-3^ ± 4×10^-5^ | 1.2×10^-2^ ± 7×10^-3^ | 1.07×10^-2^ ± 2×10^-4^ | 7.16×10^-3^ ± 1.9×10^-4^ | 9.27×10^-3^ ± 1.2×10^-4^ | 6×10^-2^ ± 9×10^-2^ | < 2 | 6.15×10^-3^ ± 1.5×10^-4^ | 0.6 ± 1.0 |
| Cesium (Cs) | 3.10×10^-3^ ± 3×10^-5^ | 0.1 ± 0.2 | 4.5×10^-3^ ± 1.8×10^-3^ | 8×10^-3^ ± 8×10^-3^ | 1.5×10^-2^ ± 1.8×10^-2^ | 9.6×10^-3^ ± 1.5×10^-3^ | < 2 | 0.1 ± 0.2 | 2.02 ± 0.02 |
| Cobalt (Co) | 3.59×10^-3^ ± 3×10^-5^ | 3×10^-2^ ± 5×10^-2^ | 4.67×10^-3^ ± 5×10^-5^ | 3.64×10^-3^ ± 9×10^-5^ | 4×10^-2^ ± 7×10^-2^ | 4.14×10^-3^ ± 5×10^-5^ | < 2 | 4.48×10^-3^ ± 1.5×10^-4^ | 0.5 ± 0.8 |
| Copper (Cu) | 3.27×10^-2^ ± 9×10^-4^ | 0.2 ± 0.3 | 4.55×10^-2^ ± 1.0×10^-3^ | 5.10×10^-2^ ± 1.1×10^-3^ | 4.51×10^-2^ ± 1.1×10^-3^ | 5.5×10^-2^ ± 5×10^-3^ | < 2 | 0.214 ± 0.009 | 5.4×10^-2^ ± 2×10^-3^ |
| Erbium (Er) | 1.003×10^-2^ ± 1.7×10^-4^ | 0.2 ± 0.4 | 3.83×10^-2^ ± 5×10^-4^ | 1.19×10^-2^ ± 2×10^-4^ | 4.019×10^-3^ ± 1.6×10^-5^ | 7.48×10^-3^ ± 9×10^-5^ | < 2 | 2.00×10^-3^ ± 7×10^-5^ | 0.5 ± 0.9 |
| Europium (Eu) | 7.75×10^-4^ ± 1.3×10^-5^ | 0.07 ± 0.11 | 9.8×10^-4^ ± 2×10^-5^ | 1.37×10^-3^ ± 4×10^-5^ | 1.263×10^-3^ ± 1.1×10^-5^ | 6.14×10^-4^ ± 2×10^-6^ | < 2 | 6.0×10^-4^ ± 3×10^-5^ | 0.4 ± 0.7 |
| Gallium (Ga) | 0.2 ± 0.3 | 0.4 ± 0.6 | 2.077×10^-3^ ± 3×10^-6^ | 2.11×10^-3^ ± 8×10^-5^ | 2.619×10^-3^ ± 1.5×10^-5^ | 0.09 ± 0.15 | < 2 | 3.34 ± 0.11 | 2.8 ± 1.4 |
| Hafnium (Hf) | 3.23×10^-2^ ± 2×10^-4^ | 0.2 ± 0.2 | 3.24×10^-2^ ± 6×10^-4^ | 3.44×10^-2^ ± 4×10^-4^ | 3.17×10^-2^ ± 2×10^-4^ | 3.45×10^-2^ ± 4×10^-4^ | < 2 | 2.90×10^-2^ ± 9×10^-4^ | 0.4 ± 0.6 |
| Lanthanum (La) | 2.243×10^-3^ ± 1.9×10^-5^ | 0.3 ± 0.5 | 2.33×10^-3^ ± 3×10^-5^ | 4×10^-2^ ± 3×10^-2^ | 2.40×10^-3^ ± 4×10^-5^ | 2.60×10^-3^ ± 4×10^-5^ | < 2 | 2.53×10^-3^ ± 7×10^-5^ | 0.6 ± 1.0 |
| Lead (Pb) | 0.35 ± 0.02 | 0.359 ± 0.019 | 0.33 ± 0.04 | 0.334 ± 0.017 | 0.39 ± 0.10 | 0.36 ± 0.02 | < 2 | 0.29 ± 0.09 | 0.8 ± 0.8 |
| Lutetium (Lu) | 2×10^-2^ ± 3×10^-2^ | 0.2 ± 0.3 | 4.28×10^-4^ ± 8×10^-6^ | 3.57×10^-4^ ± 8×10^-6^ | 5.91×10^-4^ ± 4×10^-4^ | 5.89×10^-4^ ± 9×10^-6^ | < 2 | 0.07 ± 0.10 | 0.4 ± 0.7 |
| Molybdenum (Mo) | 0.195 ± 0.005 | 0.198 ± 0.007 | 0.188 ± 0.005 | 0.184 ± 0.003 | 0.181 ± 0.005 | 0.171 ± 0.004 | < 2 | 0.160 ± 0.004 | 0.155 ± 0.013 |
| Neodymium (Nd) | 4.46×10^-3^ ± 5×10^-5^ | 0.09 ± 0.15 | 4.85×10^-3^ ± 1.9×10^-4^ | 3.01×10^-3^ ± 3×10^-5^ | 2.94×10^-3^ ± 8×10^-5^ | 4.26×10^-3^ ± 8×10^-5^ | < 2 | 2.49×10^-3^ ± 1.1×10^-4^ | 0.7 ± 1.1 |
| Nickel (Ni) | 0.10 ± 0.17 | 1.296×10^-2^ ± 9×10^-5^ | 7.16×10^-3^ ± 1.1×10^-4^ | 6.75×10^-3^ ± 1.8×10^-4^ | 9.37×10^-3^ ± 6×10^-5^ | 0.64 ± 0.02 | < 2 | 2.14×10^-2^ ± 1.1×10^-3^ | 0.3 ± 0.5 |
| Niobium (Nb) | 0.1078 ± 0.0014 | 0.146 ± 0.004 | 0.108 ± 0.003 | 0.102 ± 0.003 | 0.1127 ± 0.0018 | 0.1044 ± 0.0008 | < 2 | 0.116 ± 0.003 | 0.128 ± 0.010 |
| Rhenium (Re) | 3×10^-2^ ± 5×10^-2^ | 0.3 ± 0.5 | 1.07×10^-4^ ± 2×10^-6^ | 0.07 ± 0.12 | 8.36×10^-3^ ± 8×10^-5^ | 3×10^-2^ ± 6×10^-2^ | < 2 | 0.12 ± 0.17 | 0.6 ± 1.0 |
| Rubidium (Rb) | 2.66×10^-3^ ± 7×10^-5^ | 2.0×10^-3^ ± 6×10^-4^ | 3.10×10^-3^ ± 1.0×10^-4^ | 2.85×10^-3^ ± 1.3×10^-4^ | 2.47×10^-3^ ± 6×10^-5^ | 2.75×10^-3^ ± 1.1×10^-4^ | < 2 | 3.1×10^-3^ ± 2×10^-4^ | 0.2 ± 0.2 |
| Scandium (Sc) | 2.49×10^-2^ ± 4×10^-4^ | 0.2 ± 0.3 | 2.42×10^-2^ ± 4×10^-4^ | 6×10^-2^ ± 7×10^-2^ | 0.2 ± 0.3 | 2.31×10^-2^ ± 8×10^-4^ | < 2 | 0.3 ± 0.4 | 0.2 ± 0.4 |
| Silver (Ag) | 6 ± 2 | 0.4 ± 0.5 | 2.19×10^-2^ ± 1.7×10^-3^ | 2.553×10^-2^ ± 7×10^-5^ | 1.95×10^-2^ ± 3×10^-4^ | 1.79×10^-2^ ± 6×10^-4^ | < 2 | 1.36×10^-2^ ± 7×10^-4^ | 0.21 ± 0.14 |
| Strontium (Sr) | 1.34×10^-2^ ± 5×10^-4^ | 0.8 ± 0.7 | 5.8×10^-2^ ± 7×10^-3^ | 4.54×10^-2^ ± 1.4×10^-3^ | 0.11 ± 0.16 | 1.63×10^-2^ ± 5×10^-4^ | < 2 | 1.66×10^-2^ ± 1.0×10^-3^ | 0.74 ± 0.04 |
| Thallium (Tl) | 3.98×10^-2^ ± 4×10^-4^ | 0.6 ± 0.6 | 6.0 ± 1.2 | 13.6 ± 0.2 | 12.3 ± 1.4 | 11.0 ± 0.6 | < 2 | 5.1×10^-2^ ± 9×10^-3^ | 0.8 ± 1.2 |
| Thorium (Th) | 6.26×10^-2^ ± 1.1×10^-3^ | 6.55×10^-2^ ± 1.3×10^-3^ | 6.25×10^-2^ ± 1.6×10^-3^ | 8×10^-2^ ± 3×10^-2^ | 6.26×10^-2^ ± 1.8×10^-3^ | 6.45×10^-2^ ± 1.4×10^-3^ | < 2 | 0.3 ± 0.3 | 0.4 ± 0.6 |
| Tin (Sn) | 0.127 ± 0.002 | 0.142 ± 0.006 | 0.134 ± 0.003 | 0.132 ± 0.004 | 0.1122 ± 0.0019 | 0.115 ± 0.003 | < 2 | 8.5×10^-2^ ± 4×10^-3^ | 9.2×10^-2^ ± 6×10^-3^ |
| Uranium (U) | 2×10^-2^ ± 3×10^-2^ | 5×10^-2^ ± 8×10^-2^ | 6.86×10^-4^ ± 1.1×10^-5^ | 9.59×10^-4^ ± 1.8×10^-5^ | 6.15×10^-4^ ± 4×10^-6^ | 6.49×10^-4^ ± 1.0×10^-5^ | < 2 | 5.65×10^-4^ ± 1.9×10^-5^ | 0.2 ± 0.3 |
| Ytterbium (Yb) | 5.43×10^-3^ ± 5×10^-5^ | 0.3 ± 0.6 | 4.5×10^-3^ ± 2×10^-4^ | 9.15×10^-2^ ± 1.4×10^-3^ | 4.29×10^-3^ ± 4×10^-5^ | 6.99×10^-3^ ± 1.1×10^-4^ | < 2 | 6.41×10^-3^ ± 9×10^-5^ | 0.7 ± 1.0 |
| Yttrium (Y) | 4.58×10^-4^ ± 6×10^-6^ | 0.5 ± 0.7 | 8.8×10^-4^ ± 3×10^-5^ | 1.33×10^-3^ ± 4×10^-5^ | 8.75×10^-4^ ± 6×10^-6^ | 1.34×10^-3^ ± 3×10^-5^ | < 2 | 1.1798×10^-3^ ± 1.3×10^-6^ | 0.3 ± 0.4 |
| Zinc (Zn) | 0.71 ± 0.02 | 0.69 ± 0.03 | 0.7446 ± 0.0019 | 0.86 ± 0.06 | 0.77 ± 0.03 | 0.849 ± 0.006 | < 2 | 1.08 ± 0.07 | 1.08 ± 0.09 |
| Zirconium (Zr) | 4.47×10^-2^ ± 1.5×10^-3^ | 0.3 ± 0.4 | 4.73×10^-2^ ± 1.4×10^-3^ | 4.79×10^-2^ ± 1.9×10^-3^ | 5.06×10^-2^ ± 1.4×10^-3^ | 6.03×10^-2^ ± 1.7×10^-3^ | < 2 | 7.3×10^-2^ ± 2×10^-3^ | 0.4 ± 0.5 |

^[a]^All K_D_ values were acquired using stable elements from a multi-element standard. ^[b]^ The total procedural blank gave higher readings than the samples themselves. Hence a maximum K_D_ value was established based on the highest readings and is denoted as “< n”.

**
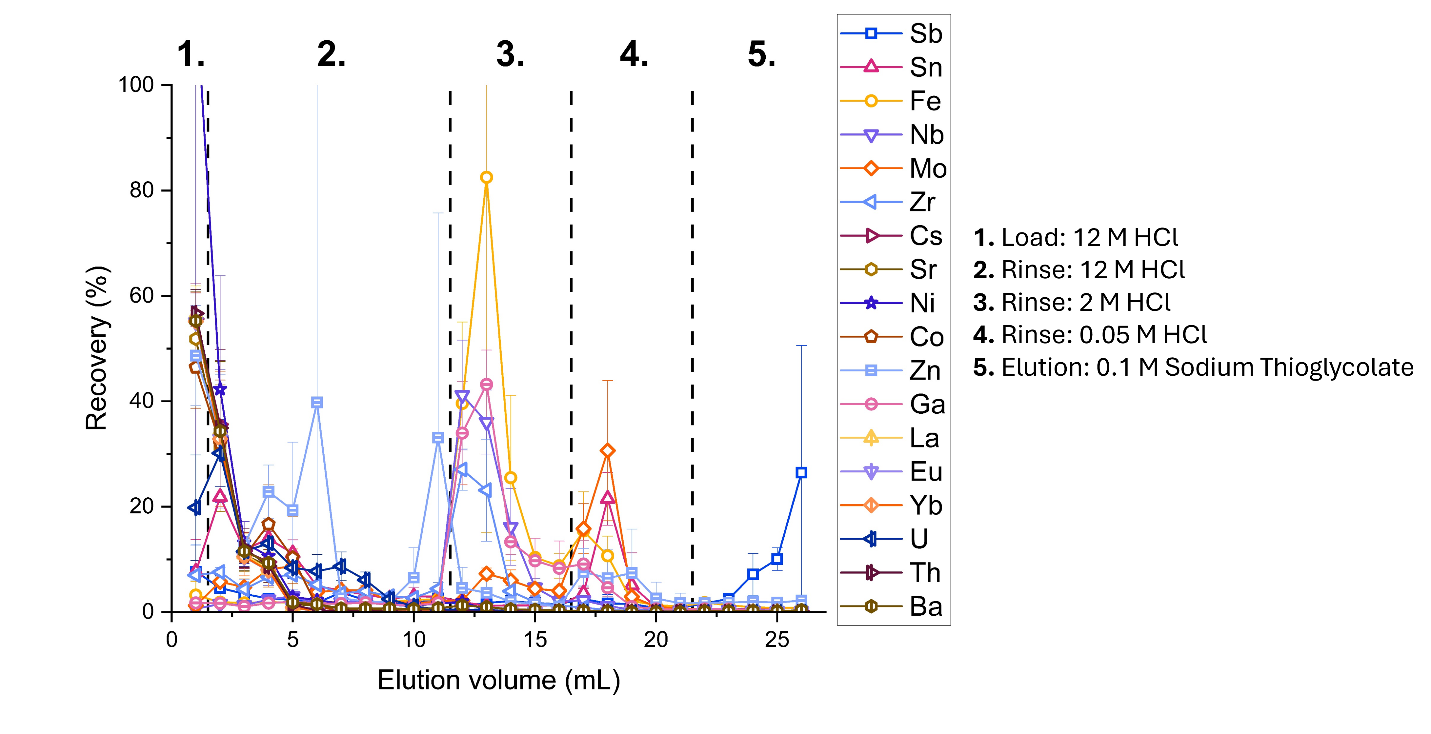
**

**Figure S1.** Elution profile for a range of stable elements on the CG71 resin support (~1 μg of each element, 1 mL column volume, 300 mg resin support, ICP-MS. n = 3).


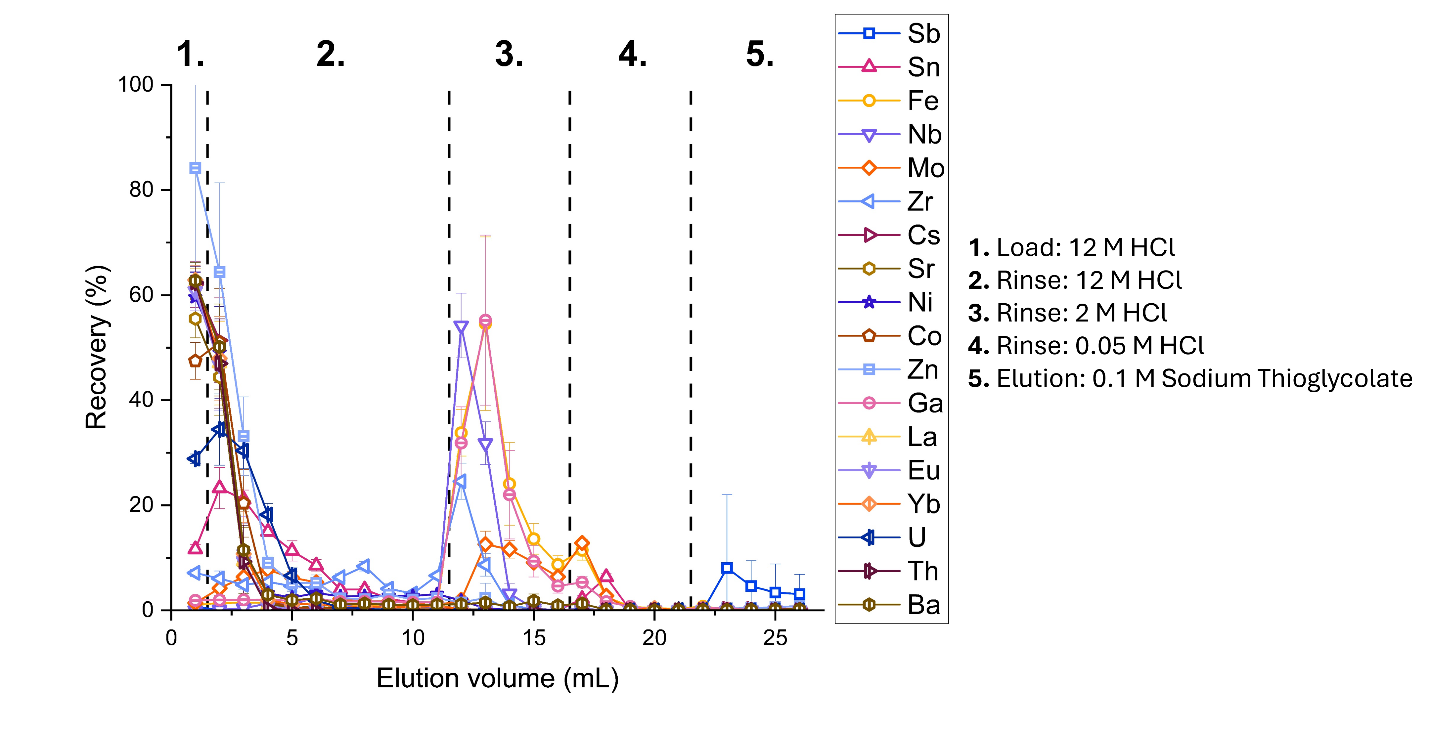


**Figure S2.** Elution profile for a range of stable elements on the DBE-71 resin (~1 μg of each element, 1 mL column volume, 300 mg resin, ICP-MS. n = 3).


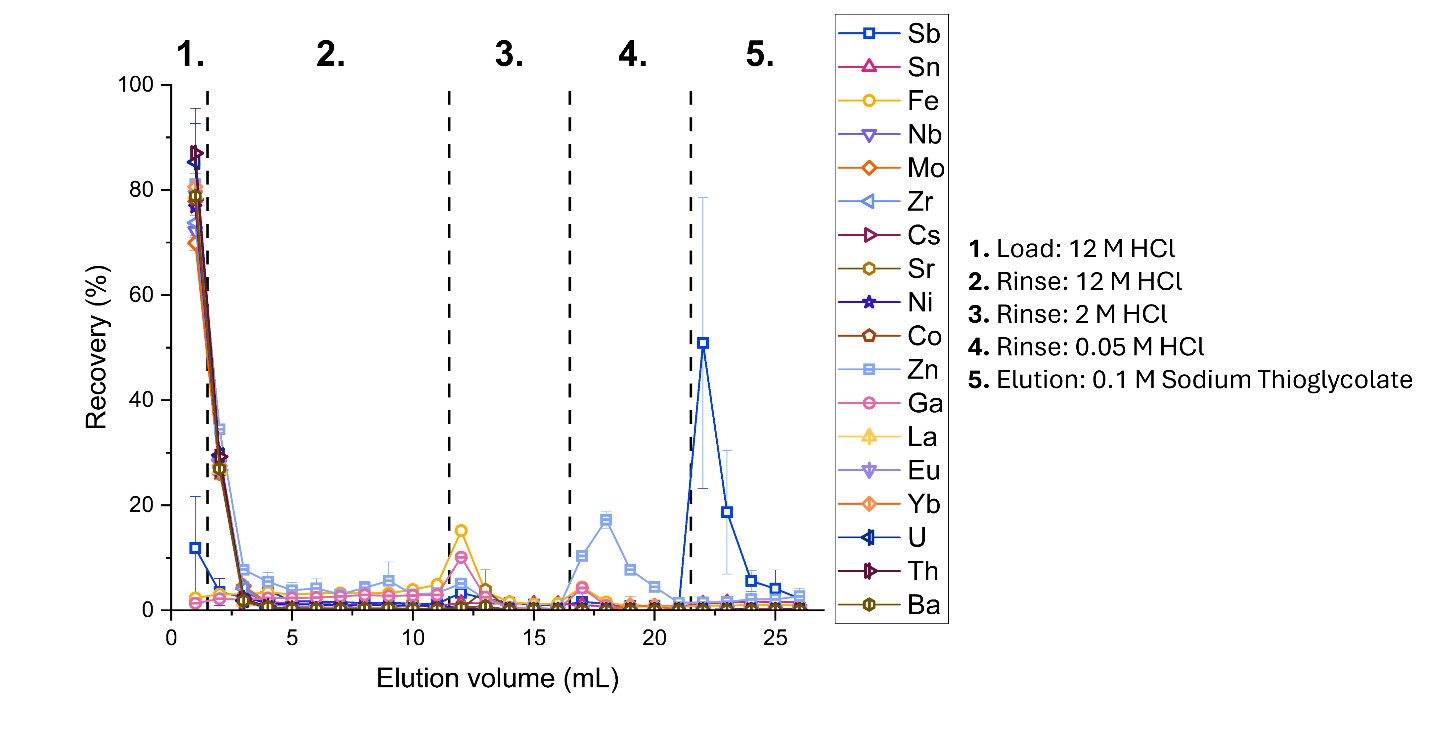


**Figure S3.** Elution profile for a range of stable elements on the DBE-300 resin (~1 μg of each element, 1 mL column volume, 200 mg resin, ICP-MS. n = 3).


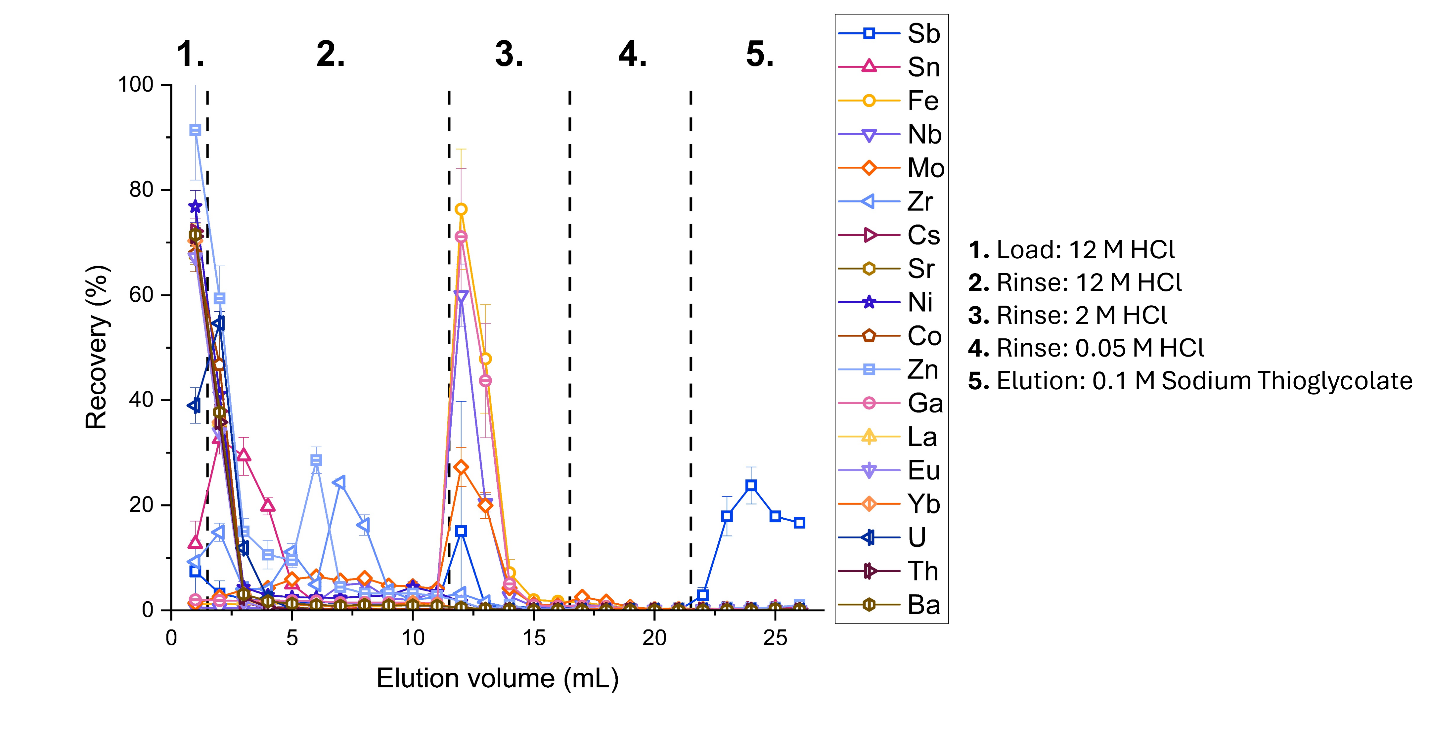


**Figure S4.** Elution profile for a range of stable elements on the DPE-71 resin (~1 μg of each element, 1 mL column volume, 300 mg resin, ICP-MS. n = 3).


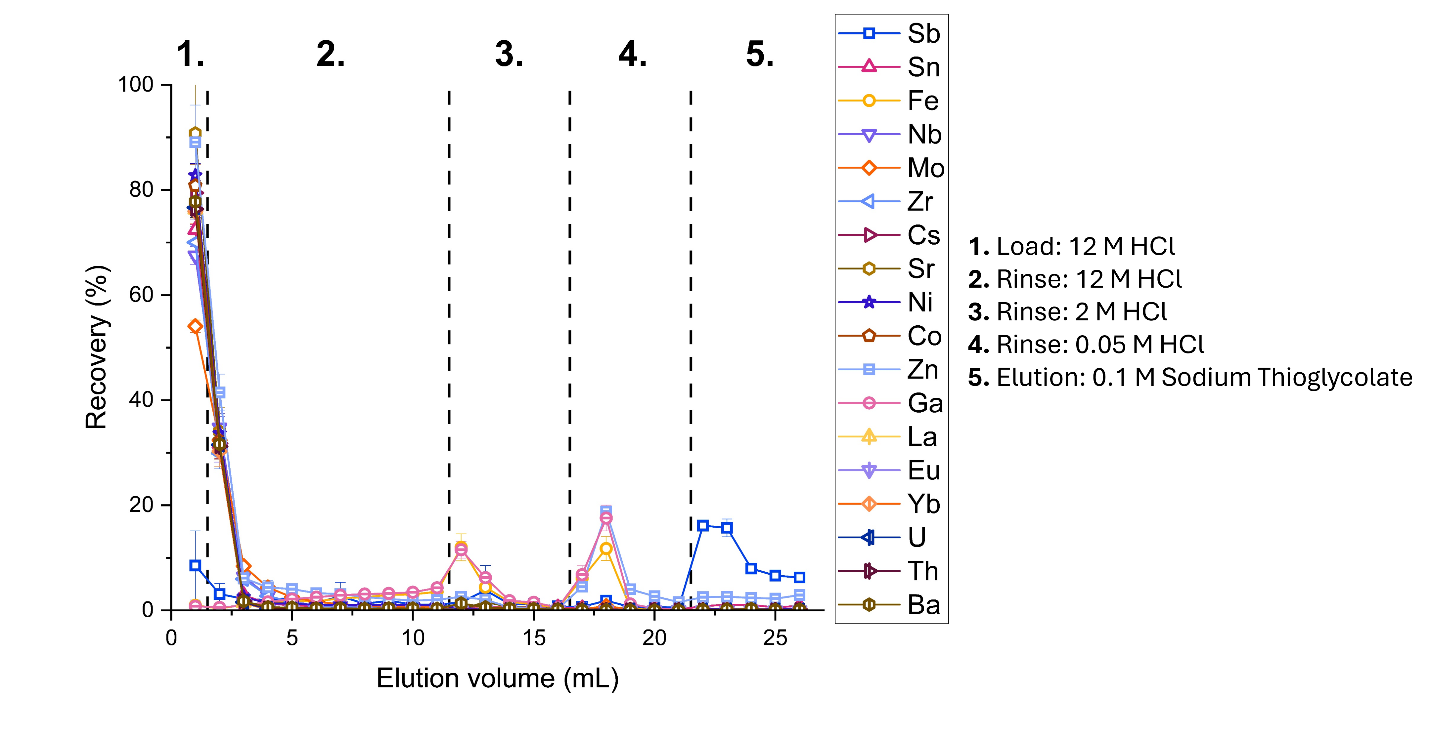


**Figure S5.** Elution profile for a range of stable elements on the DPE-300 resin (~1 μg of each element, 1 mL column volume, 250 mg resin, ICP-MS. n = 3).

**
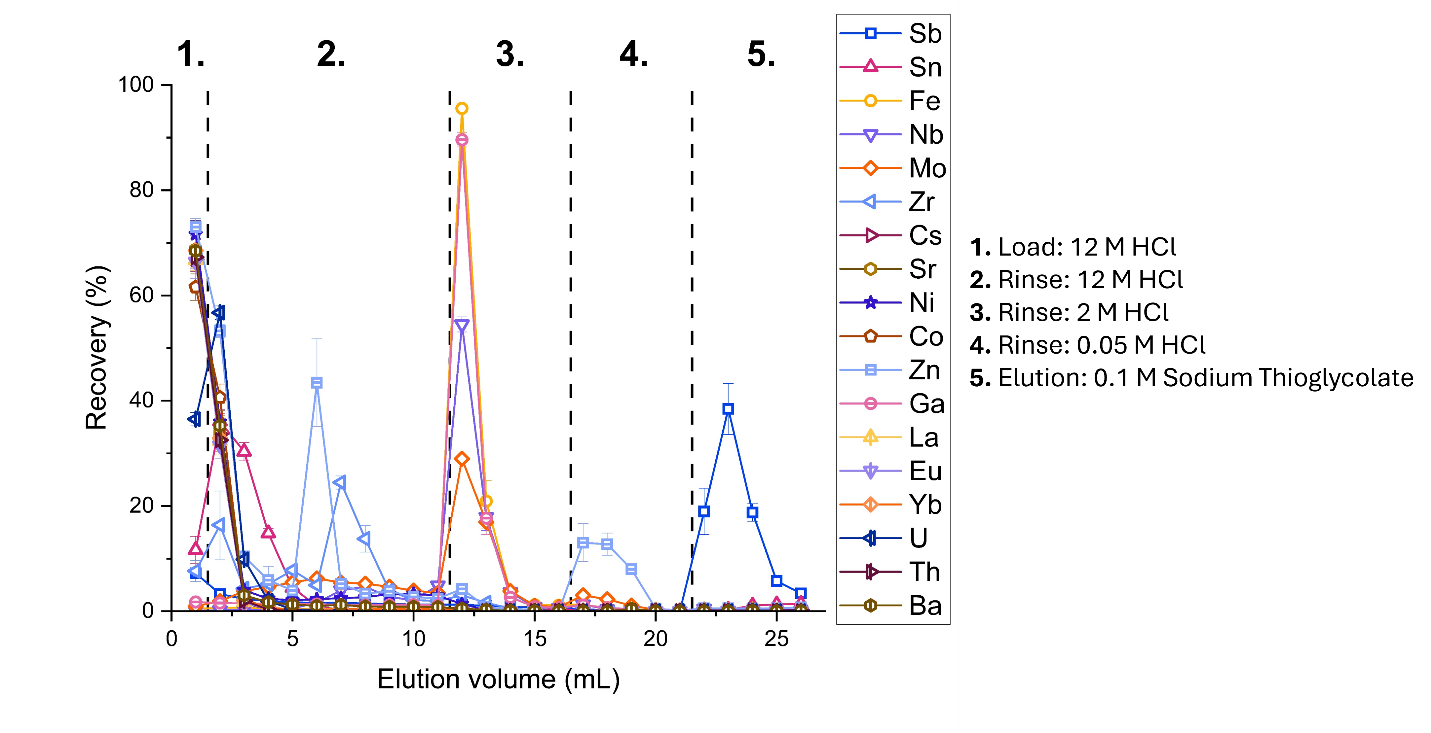
**

**Figure S6.** Elution profile for a range of stable elements on the DOE-71 resin (~1 μg of each element, 1 mL column volume, 300 mg resin, ICP-MS. n = 3).


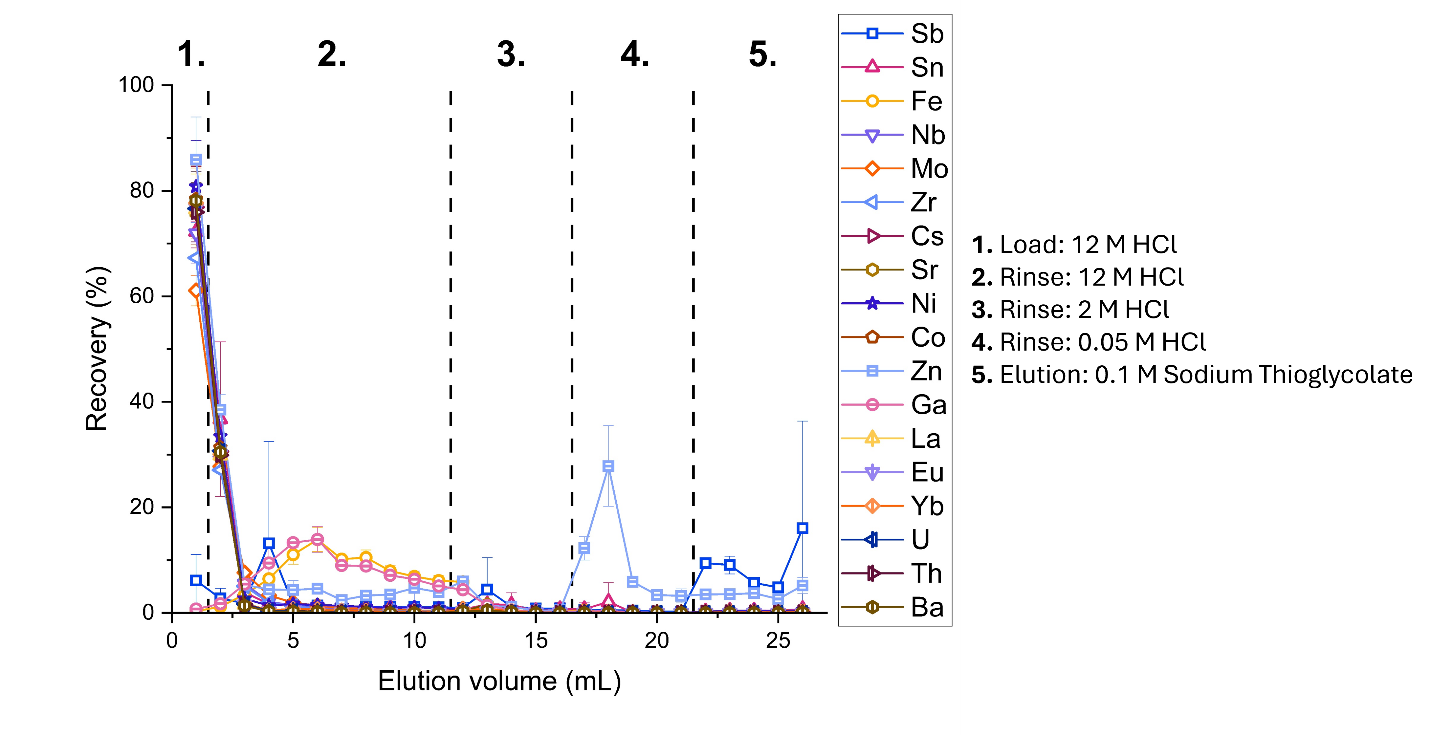


**Figure S7.** Elution profile for a range of stable elements on the DOE-300 resin (~1 μg of each element, 1 mL column volume, 250 mg resin, ICP-MS. n = 3).


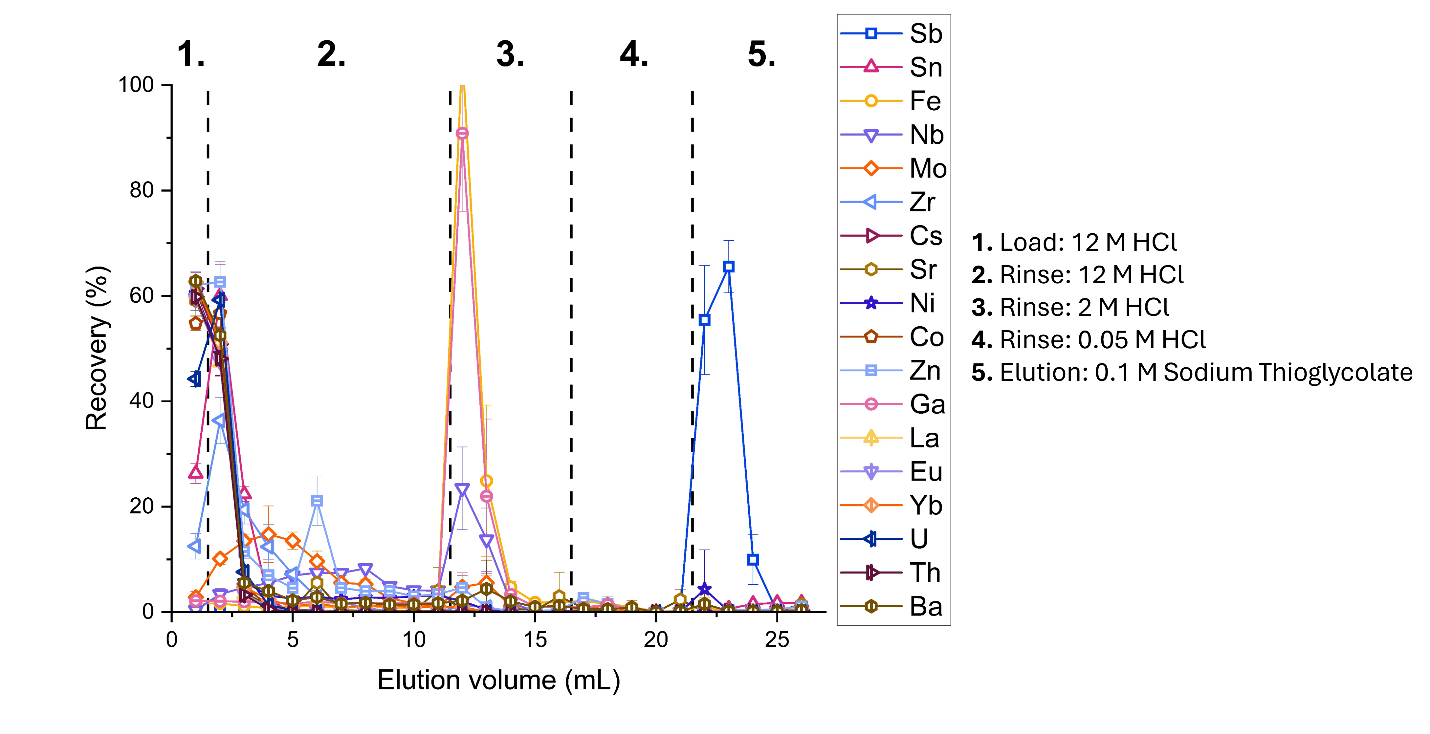


**Figure S8.** Elution profile for a range of stable elements on the TK401-71 resin (~1 μg of each element, 1 mL column volume, 350 mg resin, ICP-MS. n = 3).


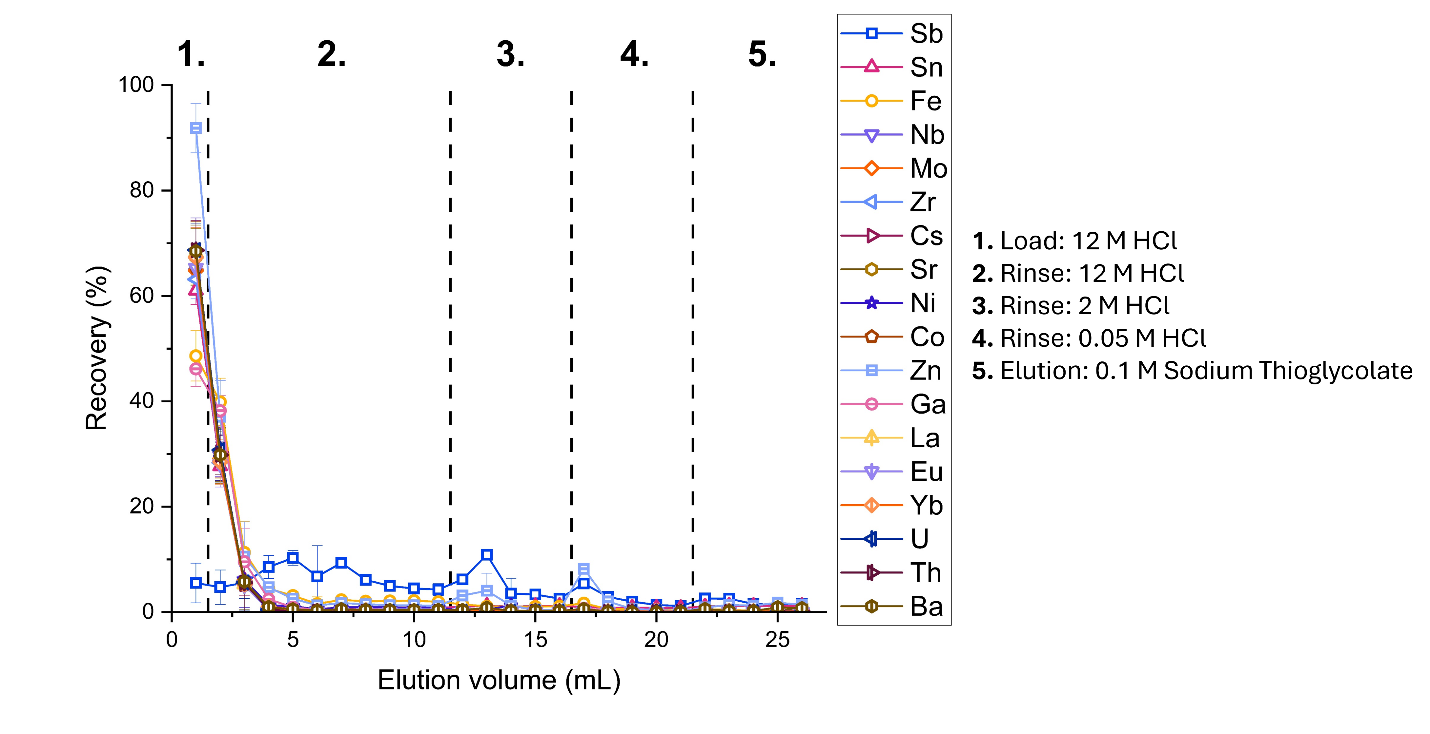


**Figure S9.** Elution profile for a range of stable elements on the TK401-300 resin (~1 μg of each element, 1 mL column volume, 300 mg resin, ICP-MS. n = 3).
